# Metathesis Reactions of a NHC‐Stabilized Phosphaborene

**DOI:** 10.1002/anie.202203345

**Published:** 2022-06-23

**Authors:** Abhishek Koner, Bernd Morgenstern, Diego M. Andrada

**Affiliations:** ^1^ Faculty of Natural Sciences and Technology Department of Chemistry Saarland University Campus C4.1 66123 Saarbrücken Germany

**Keywords:** Carbenes, Donor–Acceptor systems, Phosphaborene, Density Functional Calculations, Transfer Reactions

## Abstract

The BP unsaturated unit is a very attractive functional group as it provides novel reactivity and unique physical properties. Nonetheless, applications remain limited so far due to the bulky nature of B/P‐protecting groups, required to prevent oligomerization. Herein, we report the synthesis and isolation of a *N*‐heterocyclic carbene (NHC)‐stabilized phosphaborene, bearing a trimethylsilyl (TMS) functionality at the P‐terminal, as a room‐temperature‐stable crystalline solid accessible via facile NHC‐induced trimethylsilyl chloride (TMSCl) elimination from its phosphinoborane precursor. This phosphaborene compound, bearing a genuine B=P bond, exhibits a remarkable ability for undergoing P‐centre metathesis reactions, which allows the isolation of a series of unprecedented phosphaborenes. X‐ray crystallographic analysis, UV/Vis spectroscopy, and DFT calculations provide insights into the B=P bonding situation.

## Graphical abstract

Stable compounds with a genuine B=P double bond are rare and have limited applications given their protecting groups′ bulky nature. The preparation of a Lewis base‐stabilized phosphaborene with a B=P double bond and a trimethylsilyl (TMS) functionality at the P‐terminal is reported, which exhibits an unprecedented ability for B=P‐unit transfer reactions, allowing the isolation of B=P‐containing compounds.

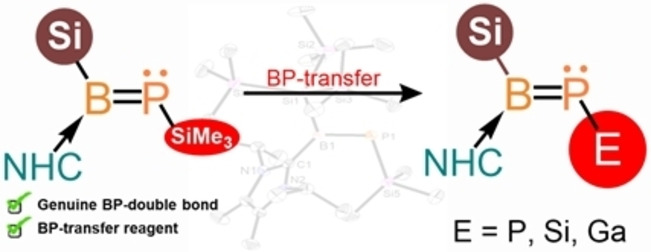

The synthesis of compounds containing multiple bonding between heavy main group elements has been a long‐standing preparative challenge.^[1]^ Introducing unsaturated units into molecular structures to build highly conjugated π‐system brings about interesting optical and electronic properties for semiconducting material.^[2]^ However, their high reactivity is an intrinsic difficulty due to relatively weak π‐bonds,^[3]^ which has eventually been circumvented by placing sterically crowded substituents to impart kinetic and thermodynamic stability.^[4]^ The ability to transfer these units adds significant synthetic value, but also, a higher level of difficulty appears by having at least one additional reactive site.

In Group 14 chemistry, transfer reagents for the introduction of multiply bonded motifs are well‐established, particularly in the case of carbon (Scheme 1). For instance, vinyl (**I**) and acetylide anions (**II**) are unique reagents to build highly π‐conjugated organic frameworks.^[5]^ Although heavier analogues remain exotic, compounds with transferable homo (**IIIa**) and heteronuclear (**IIIb** and **IIIc**) double bonds have been experimentally accomplished and applications have been showcased.^[6]^ Very recently even a Group 14/15 CP cyaphide transfer reagent has been reported by Goicoechea.^[7]^ Isoelectronic Group 13/Group 15 multiple bonds contain a significantly higher ionic contribution leading to an inherent trend to oligomerization.^[8]^ The isosteric replacement of CC by BN units are extensively applied in creating unsaturated organic/inorganic hybrid architectures.^[9]^ The BN unit features a polarized bond, but it is particularly stable due to the strong π‐bond.^[10]^ Iminoboranes, despite being well‐known as monomeric alkyne analogues,^[11]^ only recently Liu and Kong have reported BN transfer reagent (**IX**), the first‐ever example of a Group 13/15 unit transfer reagent.^[12]^ In contrast, the heavier BP‐analogues (phosphaborenes) possess a considerably weaker π‐bond,^[10]^ and, consequently, a strong tendency to dimerize.^[13]^ For sterically enriched systems, transient monomeric phosphaborene species can be thermally generated in solution and subsequently reacted with unsaturated organic compounds, including phenylacetylene, aldehydes, ketones, esters, or amides, to produce [2+2] cycloaddition or phosphaalkenes products, respectively.[^13d^, ^14^] Also, the monomeric species can be trapped by suppressing the dimerization via coordination to Lewis acids at P or Lewis bases on B terminal.^[15]^

**Scheme 1:**
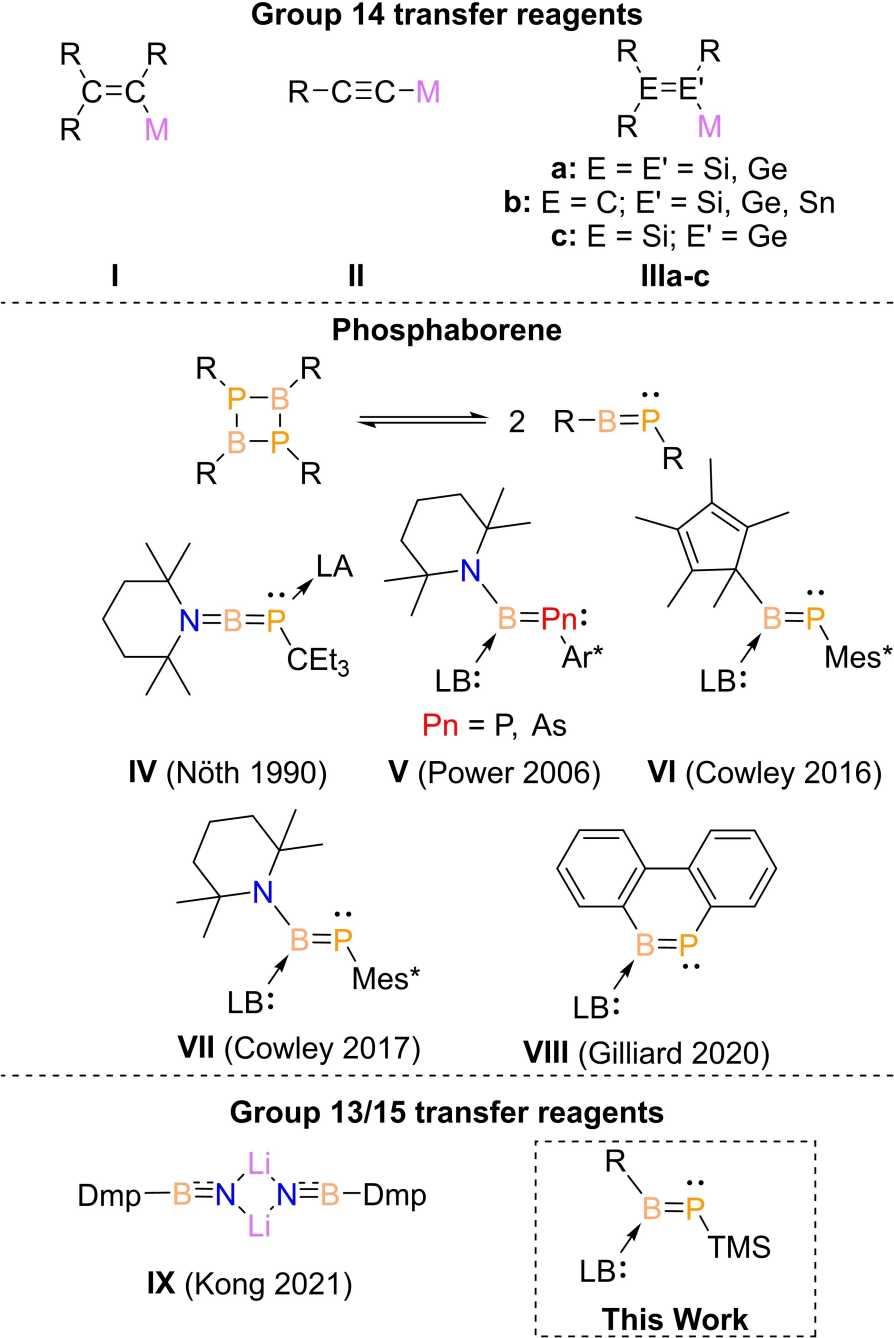
Group 14 transfer reagents: vinyl anion (**I**), acetylide anions (**II**), homo (**IIIa**) and heterodinuclear analogues (**IIIb** and **IIIc**). Previously reported examples of phosphaborene species (**IV**–**VIII**). Group 13/15 transfer reagent (**IX**). [R=organic substituents, M=Alkali metal, LA=Lewis acid, LB=Lewis base, Mes*=2,4,6‐tri‐*tert*‐butylphenyl, Ar*=2,6‐bis(2,4,6‐tri‐*iso*‐propylphenyl)phenyl, Dmp=2,6‐bis(2,4,6‐trimethyl phenyl)phenyl, TMS=trimethylsilyl].

Although a handful of phosphaborenes examples have been reported from the pioneering works of Nöth (**IV**),^[15a]^ Power(**V**),[^15b^, ^15c^] and more recently Cowley (**VI** and **VII**),[^13d^, ^14^, ^16^] and Gilliard (**VIII**),^[17]^ their chemistry remains rather unexplored. In particular, it has not yet been possible to observe a process where the BP‐unit is transferred. In the present work, we show that the use of the Lewis base‐coordination strategy can furnish phosphaborene with significantly lower steric protection and transferable B=P functionality. This new reagent undergoes unprecedented phosphaborene transfer reaction towards a triel (**Ga**), tetrel (**Si**), and pnictogen (**P**) center.

By analogy with the phosphaalkene chemistry,^[18]^ we envisaged a pendant trimethylsilyl group on the P atom as a key structural component for a transfer reagent. Phophinoborane precursors of the type R(X)B−P(TMS)R′ (X=Cl, Br) undergo 1,2‐elimination of trimethylsilyl halide both thermally^[13a]^ or by coordination with strong σ‐donor Lewis base such as DMAP or NHC.^[16]^ In this context, we chose a phosphinoborane **1** (Scheme 2) featuring a hypersilyl (TMS_3_Si) group on B‐center for stereoelectronic stabilization combined with two TMS groups on the P‐center. Thus, the reaction with a Lewis base would lead to our targeted NHC‐phosphaborene complex with remaining P‐TMS functionality.

**Scheme 2:**
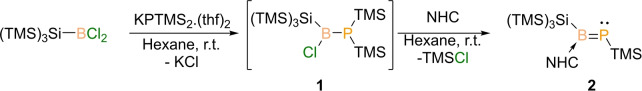
Synthesis of compound **2**. (NHC=1,3‐di*iso*propyl‐4,5‐dimethylimidazol‐2‐ylidene).

To prepare **1**, dichlorohypersilylborane (TMS_3_SiBCl_2_) was treated with an equimolar amount of KP(SiMe_3_)_2_⋅2 THF at room temperature (Scheme 2). The compound **1** (*δ*(^31^P)=−131.7 ppm, *δ*(^11^B)=108.1 ppm) formed cleanly in reaction media, but it could not be isolated in pure form due to decomposition. Hence, **1** was made in situ and used for further chemistry. Thus, when a freshly synthesized hexane solution of compound **1** was treated with an equimolar amount of 1,3‐di*iso*propyl‐4,5‐dimethylimidazol‐2‐ylidene (NHC) at room temperature, a 1,2‐elimination of TMSCl across the B−P bond was observed, resulting in the formation of the corresponding NHC‐phosphaborene adduct **2**. Compound **2** was isolated as a light‐yellow solid (80 % yield with respect to TMS_3_SiBCl_2_). The ^31^P NMR spectrum of compound **2** shows a low‐field shift (*δ*(^31^P)=163.9 ppm, in hexane) compared to the starting phosphinoborane **1**, which seconds the change of hybridization and geometry on the P‐center. The ^11^B NMR spectrum of compound **2** shows a strongly high‐field shifted resonance signal at *δ*(^11^B)=57.6 ppm, compared to **1**. When compared to the analogous adduct from **VI** (*δ*(^31^P)=192.9 ppm),^[16]^ the compound **2** shows a significant high‐field shift in ^31^P NMR spectrum (*δ*(^31^P)=163.9 ppm), indicating a highly electron‐rich P‐center. The ^1^H NMR spectrum shows a characteristic doublet for the P−SiMe_3_ protons at 0.19 ppm (^4^ *J*_P,H_=2.7 Hz). The UV/Vis spectrum exhibits the main absorption band at *λ*_max_=336 nm (*ϵ*=837 cm^−1^ M^−1^, Figures S31 and S32), which based on TD‐DFT analysis is assigned to B=P π→π* transitions (Table S3).

The solid‐state structure of **2** was determined by single‐crystal X‐ray diffraction (SC‐XRD, Figure 1).^[19]^ The molecular structure reveals a trigonal planar geometry at the B‐center with a sum of bond angles of 360°. The NHC ring plane is perpendicular to the P1−B1−Si1 plane (P1−B1−C1−N1 dihedral angle=87.3(1)°), reflecting reduced participation of the π‐acidity of the carbene. The B1−C1 bond is shorter (1.577(4) Å) than a conventional B−C single bond (1.60 Å),^[20]^ but still in a similar range as that of **VI** (1.582(2) Å),^[16]^ suggesting a strong interaction. The B−P bond length for compound **2** (1.817(3) Å) is slightly longer than in **VI** (1.807(2) Å), and falls close to the typical lengths of B=P double bonds (1.80 Å).^[20]^ The B1−Si1 (2.040(3) Å) and P1−Si5 (2.242(1) Å) distances are within the range of conventional single bonds and are connected through a rather planar central BP central moiety (twist angle=2.5(1)°).

**Figure 1:**
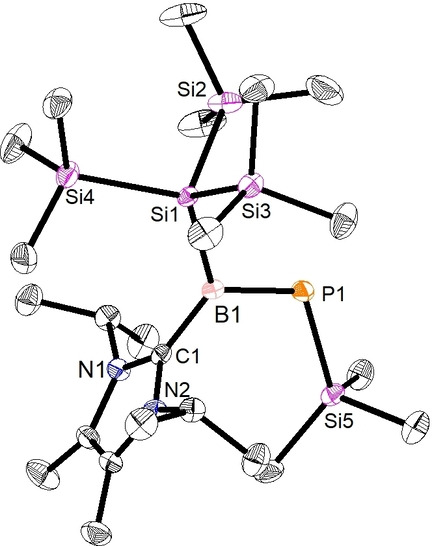
Solid‐state structure of phosphaborene **2**. Thermal ellipsoids at 50 %. Hydrogen atoms were omitted for clarity. Selected experimental and calculated [BP86‐D3(BJ)/def2‐SVP] bond lengths [Å] and angles [°]: B1–P1 1.817(3) [1.825], P1–Si5 2.242(1) [2.271], B1–C1 1.577(4) [1.563]; C1‐B1‐P1 122.2(2) [123.9], Si1‐B1‐P1 114.0(1) [115.8], B1‐P1‐Si5 114.2(1) [111.7], C1‐B1‐P1‐Si5 2.7(2) [0.3].

To gain further insight into the electronic structures of **2**, we performed DFT calculations (BP86‐D3(BJ)/def2‐SVP, see the ESI for details). The optimized geometry of **2** is in good agreement with the structure determined by SC‐XRD. The frontier Kohn–Sham (KS) molecular orbitals reveal a lone‐pair at phosphorus atom HOMO−1, while the HOMO and LUMO are the B=P π and π* orbitals (Figure 2a). A natural bond orbital (NBO) analysis confirms the presence of a lone‐pair on P atom with *sp*‐character, whilst two further NBOs correspond to B−P σ‐bonding orbitals (46.2 % (B) and 53.8 %(P)), and a polar B=P π‐orbital (38.5 %(B) and 61.5 %(P)). Consistently, Wiberg bond indices (WBIs) suggest a significant B=P double bond character (1.78) and B−C_carb_ with single bond character (0.88). Natural population analysis (NPA) indicates an electron‐rich BP unit with −0.36e and −0.28e on B and P atoms, respectively, mainly donated by the coordinated NHC fragment (+0.44e, Table S1). Natural Resonance Theory (NRT) also supports the description above (Figure 2b), where the main resonance structures **2A** and **2B**, weigh 86.9 % and 13.1 %, respectively. This is in line with the intriguing experimental observation about the dependence of the ^31^P NMR chemical shift of compound **2** on the solvent polarity. We observed a progressing high‐field shift of the *δ*(^31^P) value with the increased solvent polarity. In non‐polar solvents, like hexane, the *δ*(^31^P) is 163.9 ppm, while in C_6_H_6_, THF and DCM the *δ*(^31^P) appears at 158.4, 155.7 and 148.7 ppm, respectively (see Supporting Information, Figure S7). The more polar the solvent the more contribution of **2B** is expected, shielding the phosphorus atom and hence leading to high‐field shifted resonance.

**Figure 2:**
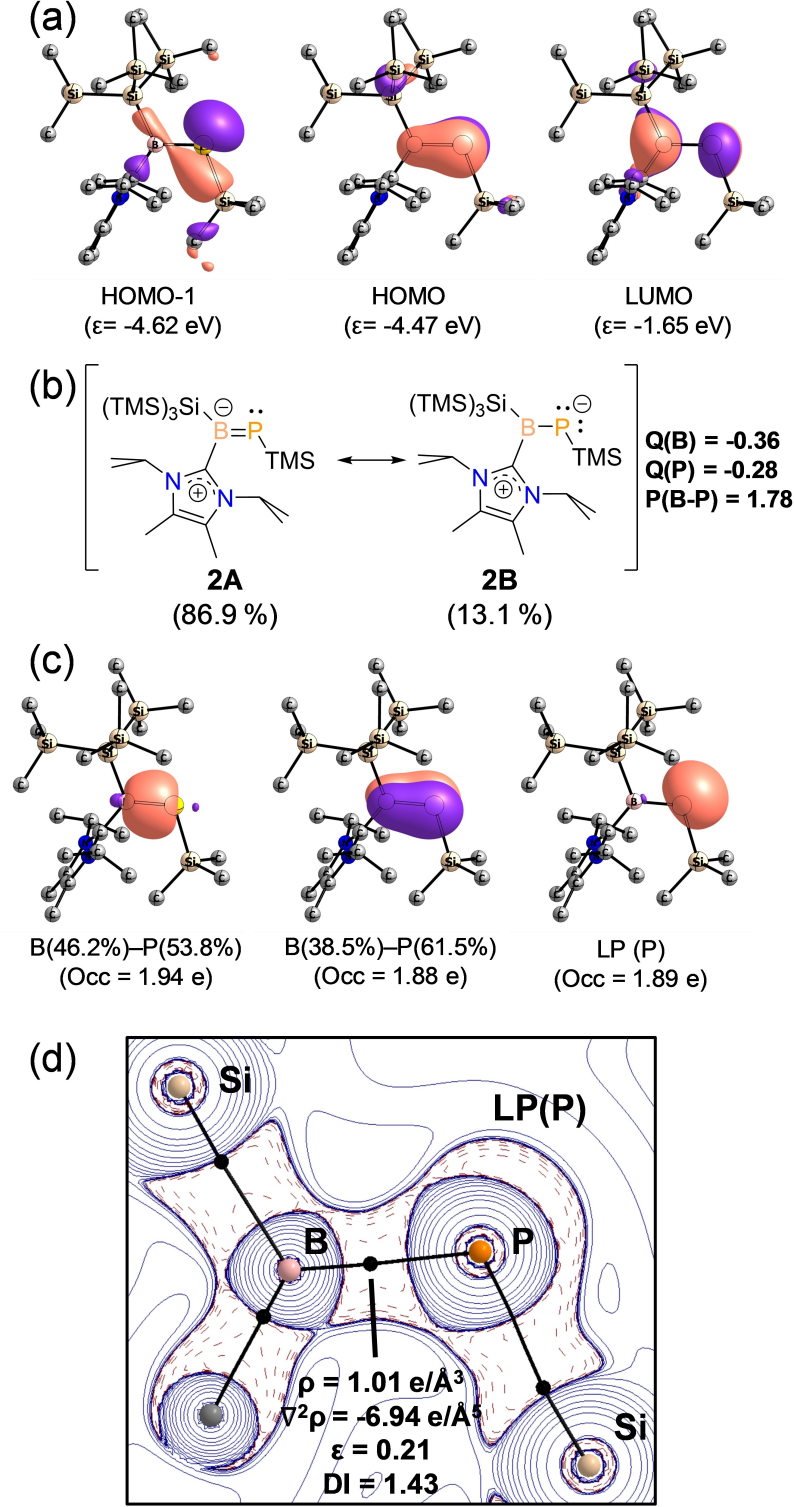
Bonding analysis of **2** (BP86‐D3(BJ)/def2‐TZVPP). a) KS‐Molecular Orbitals. b) Resonance structures (weight by NRT), partial atomic charges (NPA) and B=P Wiberg bond order. c) NBOs. d) 2D Laplacian distribution ∇^2^ *ρ*(*r*) in the N1−B1−N2 plane. Dashed red lines indicate areas of charge concentration (∇^2^ *ρ*(*r*)<0) while solid blue lines show areas of charge depletion (∇^2^ *ρ*(*r*)>0), bond paths (black lines), and bcps (black dots).

We also analyzed the electron density distribution with atoms in molecules (QTAIM).^[21]^ The Laplacian distribution ∇^2^ *ρ*(*r*) in the C1−B1−P1 plane is depicted in Figure 2d. The Laplacian plot shows an electron accumulation on the phosphorus atom localized on the σ‐system and the electron density of the B−P bond critical point (*ρ* ^BCP^=1.01 e Å^−3^), which is shifted towards the B. The corresponding delocalization index (DI=1.43) and the ellipticity (*ϵ*=0.21) support a strong B=P double bond character.

We have evaluated the stability of the chemical bond between the NHC and phosphaborene by using Energy Decomposition Analysis (EDA, see Supporting Information Table S2).^[22]^ The dissociation energy is 65.0 kcal mol^−1^ which is comparable to former phosphaborene adducts 64.7 kcal mol^−1^ (**VI**) and 51.7 kcal mol^−1^ (**VII**). The preparation energy (Δ*E*_prep_) is relatively small for NHC (3.1 kcal mol^−1^), since the coordination does not carry significant geometry deformation. For the monomeric phosphaborene, the energy is higher given the bending of Si1−B1−P1 bonding angle requests higher energy penalties (29.4 kcal mol^−1^). The interaction energy (Δ*E*_int_) follows the same trend as the dissociation energy. According to EDA dissection of the interaction energy, the bonding consists of 7.8 % dispersion, 46.0 % electrostatic and 46.2 % orbital interactions. The absolute values indicate a strong Pauli repulsion between NHC and substituents on the B atom.

The new Lewis base‐stabilized phosphaborene adduct **2** is indefinitely stable at ambient temperature, in the solid state and in solution, under argon atmosphere. The TMS group on the phosphorus atom is particularly attractive for further functionalization via TMSCl elimination under mild conditions. In this vein, we have explored the reactivity towards with main group 13–15 electrophiles carrying E−Cl functionality. Within the pnictogen series, we chose TipPCl_2_ (Tip=2,4,6‐*i*Pr_3_C_6_H_2_) as the electrophile. In equimolar ratio, the reaction with **2** shows clean conversion to compound **3**, containing a newly formed B=P−P motif (Scheme 3). The formation of the P−P bond can be monitored by ^31^P{^1^H} NMR spectroscopy, where two new doublet (^1^ *J*_P,P_=331 Hz) resonance signals appear at 123.5 ppm and 191.3 ppm for the sp^3^−P and sp^2^−P, respectively. The ^11^B NMR chemical shift occurs at 64.7 ppm, slightly low‐field shifted with respect to **2**, and agrees with a tricoordinated boron center. Additionally, the ^1^H NMR spectrum shows the disappearance of the P‐TMS doublet at 0.19 ppm, which endorses a P−P bond formation via TMSCl elimination. Compound **3** was isolated as a bright yellow powder in 75 % yield by washing the crude reaction residue with hexane at −20 °C. UV/Vis spectrum show a small bathochromic shift for the main absorption band at *λ*_max_=361 nm (*ϵ*=1024 cm^−1^ M^−1^). SC‐XRD analysis unambiguously confirms the nature of compound **3** (Figure 3a). Notably, the molecular structure exhibits an intact B=P chemical bond and the formation of a new P−P bond. The B−P bond length of compound **3** (1.817(2) Å) is the same as in compound **2**, and the new functional group has a planar B=P−P unit (C1−B1−P1−P2 dihedral angle of 2.3(1)°). All attempts to introduce a second B=P unit on the pnictogen (P) center by either reacting **2** with TipPCl_2_ in 2 : 1 ratio or reacting **2** and **3** in 1 : 1 ratio were unsuccessful, given the steric constraints at the P‐center caused by the Tip group.

**Scheme 3:**
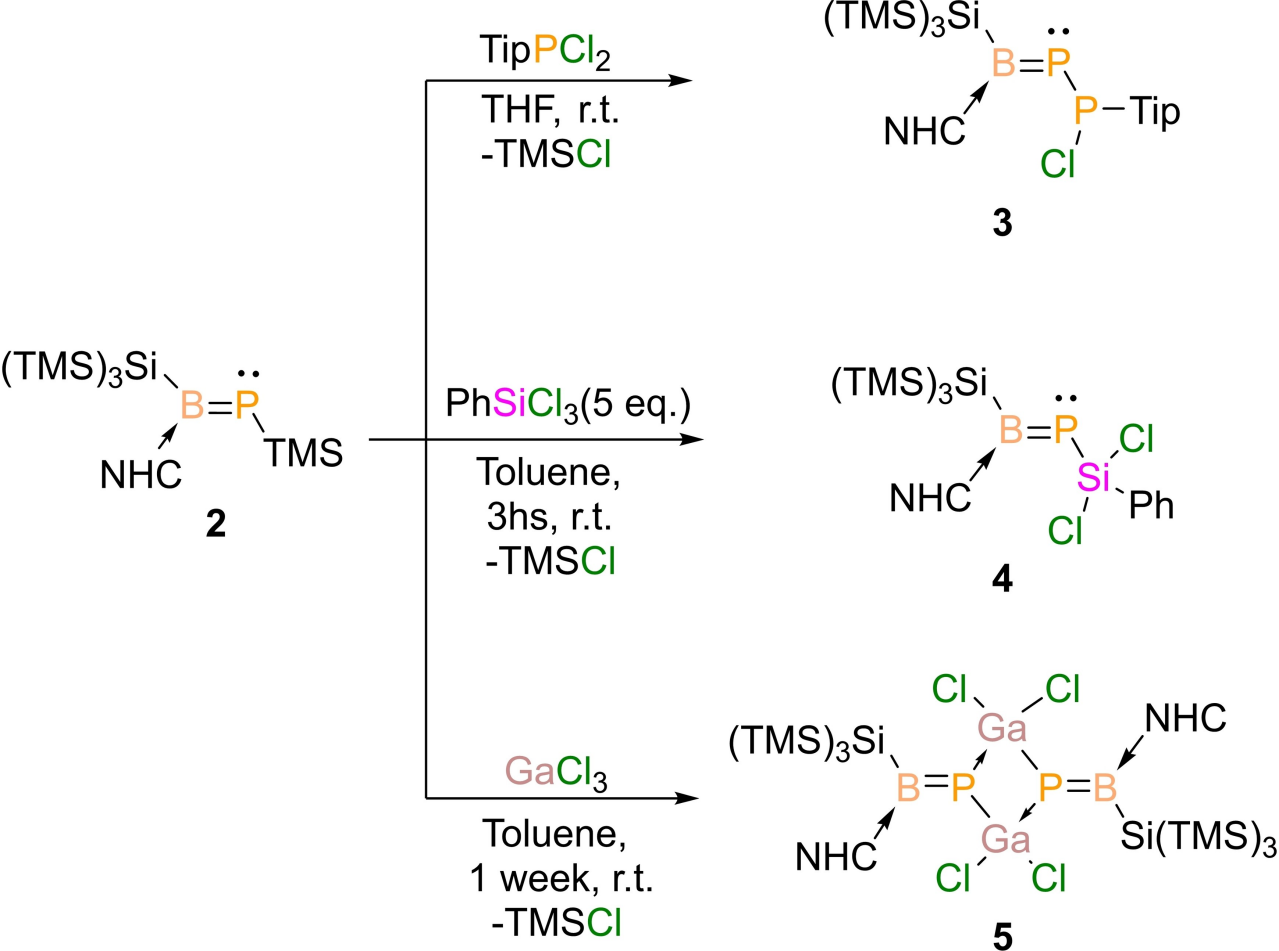
Metathesis reactions of phosphaborene unit to pnictogen (**3**), tetrel (**4**) and triel (**5**) centres (Tip=2,4,6‐*i*Pr_3_C_6_H_2;_ NHC=1,3‐di*iso*propyl‐4,5‐dimethylimidazol‐2‐ylidene).

**Figure 3:**
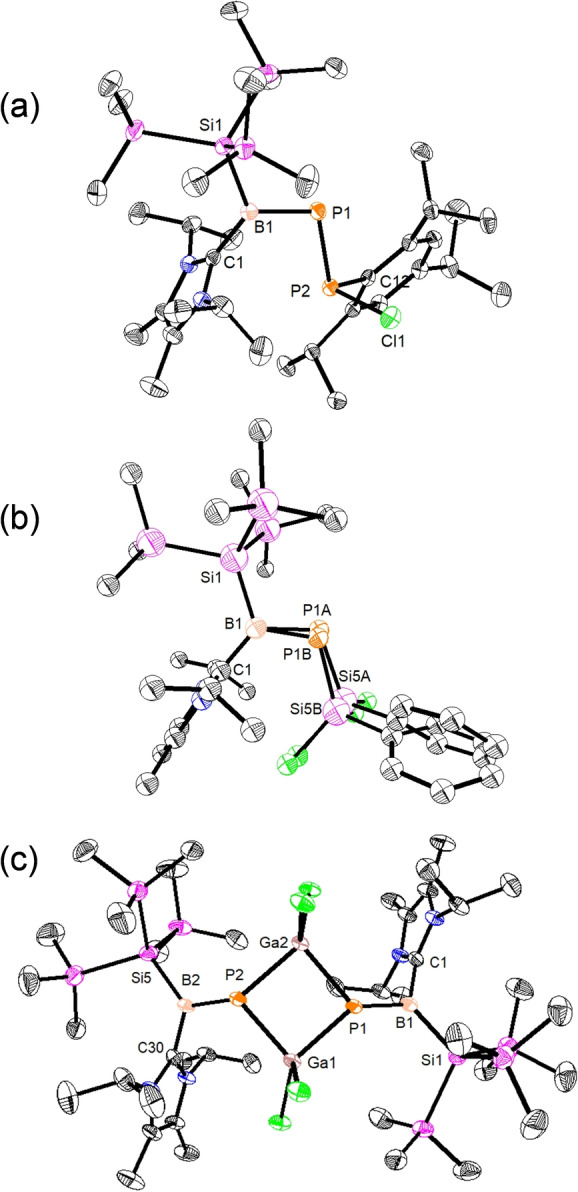
Solid‐state structure of compounds **3** (a), **4** (b), **5** (c). Thermal ellipsoids at 50 %. Hydrogen atoms were omitted for clarity. Selected experimental and calculated [BP86‐D3(BJ)/def2‐SVP] bond lengths [Å] and angles [°]: **3**: B1–P1 1.817(2) [1.832], C1–B1 1.578(2) [1.567]; C1‐B1‐P1‐P2 2.3(1) [4.1]. **4**: B1–P1A/B 1.824(7)/1.809(9) [1.827], B1–C1 1.579(2) [1.565]; C1‐B1‐P1A/B‐Si5A/B 1.2(7)/4.1(8) [6.3]. **5**: B1–P1 1.814(4) [1.822], B1–C1 1.587(4) [1.575]; C1‐B1‐P1‐Ga1 8.9(3) [11.0].

Similarly, the reaction of **2** with PhSiCl_3_ afforded compound **4**, where the B=P‐unit is now transferred to a tetrel (Si)‐center. An excess of PhSiCl_3_ reagent is necessary to complete the reaction, as the stoichiometric reaction was kinetically slow. Attempts to increase the reaction rate using polar solvents, such as THF or DCM, led to side reactions. Once more, a second B=P‐unit transfer was not possible due to steric limitations. Compound **4** was purified by crystallizing the crude residue, as a pale yellow solid in 50 % yield, from hexane at −20 °C. In this case, the UV/Vis absorption band is similar to compound **2**, i.e. 338 nm (*ϵ*=770 cm^−1^ M^−1^). The ^31^P and ^11^B NMR spectra of compound **4** show signals at 92.2 and 66.8 ppm, respectively. The solid‐state molecular structure of compound **4** (Figure 3b) shows the presence of uncompromised B=P bond with bond lengths of 1.824(7) Å (for B1−P1 A) and 1.809(9) Å (for B1−P1B).

Finally, we chose GaCl_3_ as the electrophilic reagent to prove transferability to triel centers. Compound **2** slowly reacts with GaCl_3_ in toluene to form compound **5**, with a Ga_2_P_2_ four‐membered ring and two exocyclic B=P double bonds (Scheme 2). Compound **5** was isolated as bright yellow powder via crystallization from toluene in 70 % yield (*λ*_max_=382 nm, *ϵ*=1024 cm^−1^ M^−1^). The ^31^P NMR spectrum of **4** (in CDCl_3_) shows a signal at 88.9 ppm, but no signal was observed in the ^11^B NMR spectrum. Nonetheless, in THF solvent, the ^11^B NMR spectrum shows a clear signal at 66.9 ppm, agreeing with the expected tricoordinated boron center chemical shift. The ^31^P NMR spectrum in THF exhibits low‐field chemical shift to 115.1 ppm, due to conversion of the dimeric four‐membered Ga_2_P_2_‐ring into monomeric B=P−GaCl_2_(THF). The solid‐state structure of **5** underlines the presence of two exocyclic BP‐double bonds with a length of 1.818(4) Å (Figure 3c). The middle four‐membered Ga_2_P_2_‐ring comprises GaP single bonds of 2.3349(9) Å. The sum of bond angles of 360° shows a trigonal planar geometry on the B‐centers, whereas the sum of bond angles on P1 (357.6°) shows a slight deviation from planarity.

Our observation on the *δ*(^31^P) NMR of compound **5** in different solvents prompted us to explore the possibility of synthesizing the monomeric species by breaking the Ga_2_P_2_‐ring with a strong σ‐donor Lewis base like a NHC. And indeed the reaction of dimeric **5** with two equivalents of NHC in THF solution furnishes the monomer **6**, where the Ga atom is coordinated to NHC (Figure 4). The ^31^P NMR spectrum of compound **6** (*δ*(^31^P)=153.4 ppm) shows a strong low‐field chemical shift compared to the dimer, and the ^11^B NMR spectrum (*δ*(^11^B)=60.2 ppm) supports the presence of tricoordinated B‐center. Figure 4 displays the solid‐state molecular structure of **6**, where B−P bond length (1.807(2) Å) falls into the double bond range, and the B1−P1−Ga1−Cl2 (5.34(5)°) dihedral angle indicates a co‐planarity of the B=P and Ga−Cl bonds.

**Figure 4:**
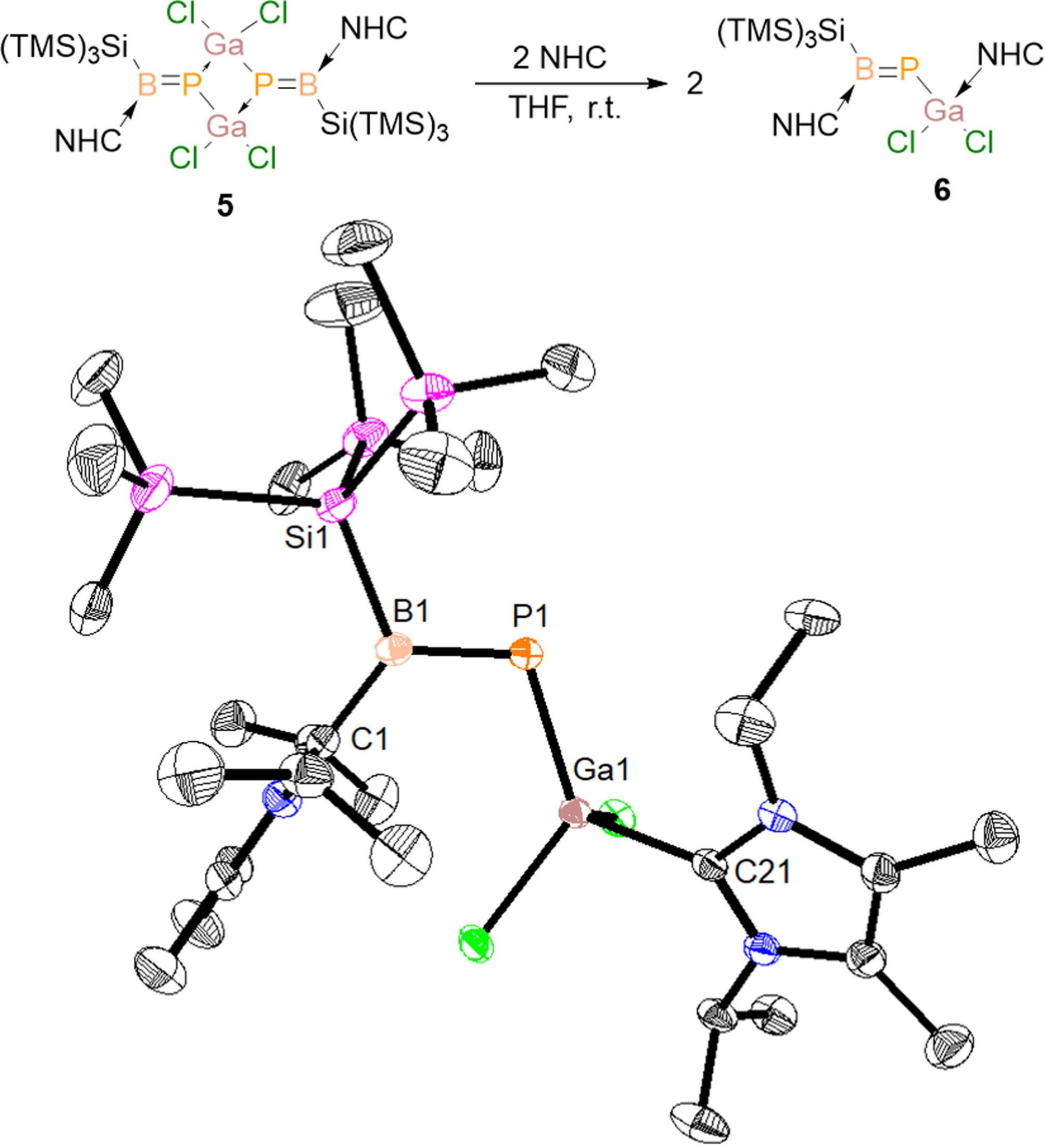
Synthesis of compound **6** and solid‐state structure. Hydrogen atoms were omitted for clarity. Thermal ellipsoids at 50 %. Selected experimental and calculated [BP86‐D3(BJ)/def2‐SVP] bond lengths [Å] and angles [°]: **6**: B1–P1 1.807(2) [1.822], C1–B1 1.579(2) [1.564], C21–Ga1 2.062(1) [2.091]; C1‐B1‐P1‐Ga1 2.3(1) [2.6].

In conclusion, we have established the synthetic route to access a Lewis base‐stabilized phosphaborene adduct with a pendant TMS functionality at the phosphorus atom. This novel adduct exhibits remarkable thermal stability both in the solid and solution phases, and its suitability as synthons for synthesizing a series of phosphaborenes has been demonstrated by reactions with electrophiles from Groups 13–15 of the periodic table. Notably, species **2**–**6** represent the first examples of triel, tetrel, and pnictogen phosphaborenes. X‐ray crystallography, UV/Vis, and DFT calculations revealed the retention of the uncompromised BP double‐bonded unit under these chemical transformations. The preparation of the first B=P transfer reagent **2** opens up the possibility of synthesizing various B=P‐containing compounds.

## Conflict of interest

The authors declare no conflict of interest.

## Supporting information

As a service to our authors and readers, this journal provides supporting information supplied by the authors. Such materials are peer reviewed and may be re‐organized for online delivery, but are not copy‐edited or typeset. Technical support issues arising from supporting information (other than missing files) should be addressed to the authors.

Supporting Information

Supporting Information

Supporting Information

Supporting Information

Supporting Information

Supporting Information

## Data Availability

The data that support the findings of this study are available in the Supporting Information of this article.
