# Supplementary material for: Metathesis Reactions of a NHC‐Stabilized Phosphaborene
Source: Angew Chem Int Ed Engl. 2022 Jun 23;61(31):e202203345. doi: 10.1002/anie.202203345 (PMC9401048; doi:10.1002/anie.202203345)
Supplement: Supplementary file 6 — Supporting Information [file ANIE-61-0-s006.pdf]

Angewandte Chemie
Eine Zeitschrift der Gesellschaft Deutscher Chemiker

Supporting Information

Metathesis Reactions of a NHC-Stabilized Phosphaborene

*A. Koner, B. Morgenstern, D. M. Andrada**

Table of Contents

Synthetic protocol and NMR spectra for compounds 1-6 .... *4*
UV-Visible Spectra *12*
IR Spectra *25*
Crystallographic Details *30*
Computational Details *35*
Cartesian xyz coordinates and Energies in Hartrees *56*
References *74*

Materials and Method

All syntheses and manipulations were carried out under an inert gas atmosphere (Ar or N_2) using standard *Schlenk* techniques or a glove box (MBraun Unilab Plus). The glassware was pre-dried in oven at 125 °C and heated *in vacuo* prior to use. Organic solvents *n*-hexane, benzene, THF and toluene were taken directly from a solvent purification system (Innovative Technology PureSolv MD5). Deuterated solvents were dried over appropriate drying agents, distilled, and stored inside a glove box. NMR spectra were recorded at 300 K on a Bruker Avance IV HD 400 (^{1}H: 400.13 MHz, ^{13}C: 100.61 MHz, ^{11}B: 128.38 MHz, ^{29}Si: 59.6 MHz, ^{31}P: 121.5 MHz), using borosilicate-containing NMR tubes. Chemical shifts (in δ, ppm) are referenced to the residual[1] solvent signal(s): C_6D_6 (^{1}H, 7.16; ^{13}C, 128.06). Fourier-transform IR spectra were acquired on a Bruker Vertex 70 spectrometer in attenuated total reflectance (ATR) mode. Elemental analyses were performed on an elemental analyzer Leco CHN-900 and/or an elementar 161vario Micro Cube. Single crystal X-ray diffraction analysis were carried out at low temperatures on Bruker AXS D8 Venture diffractometer operating with graphite monochromated Mo Kα radiation. Structure solution and refinement were performed using SHELX.[2] These data can be obtained free of charge from The Cambridge Crystallographic Data Centre via http://www.ccdc.cam.ac.uk. UV/Vis spectra were measured using a Shimadzu UV-2600 spectrometer in quartz cells with a path length of 0.1 cm. TMSCl (Sigma Aldrich), ^{n}BuLi (1.6 M in n-hexane) (Alfa aesar), $PhSiCl_3$ (Acros Chemicals), $GaCl_3$ (Sigma Aldrich) and BCl_3 (Acros Chemicals) were purchased from commercial sources and used without further purification. NHC^{iPrMe},[3] $(Me_3Si)_3SiBCl_2$,[4] $KP(SiMe_3)_2$,[5] and $TipPCl_2$[6] were synthesized according to literature procedure.

Synthetic protocol and NMR spectra for compounds 1-6

Compound 1: 3.32 g (10.09 mmol) of $(Me_3Si)_3SiBCl_2$ and 3.82 g (10.59 mmol, 1.05 eq) of $KP(SiMe_3)_2$ were taken together in a 250 mL Schlenk flask and 120 mL of *n*-hexane was added to it at room temperature. The mixture was stirred at room temperature for 2 hours at which point all of $(Me_3Si)_3SiBCl_2$ was consumed and formed $(Me_3Si)_3SiB(Cl)P(SiMe_3)_2$, which is visible in **Figure S1-S3**. Unfortunately, the compound **1** could not be isolated in pure form and was used as a *n*-hexane solution, thereafter.

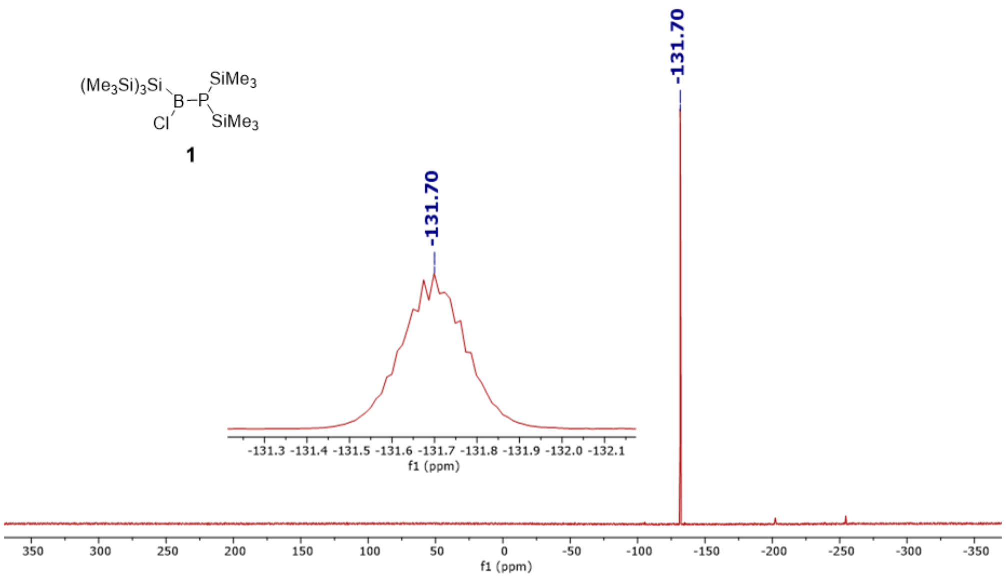

Figure S1. ^{31}P NMR (161.9 MHz, Hexane, 300 K) spectrum of compound **1**.

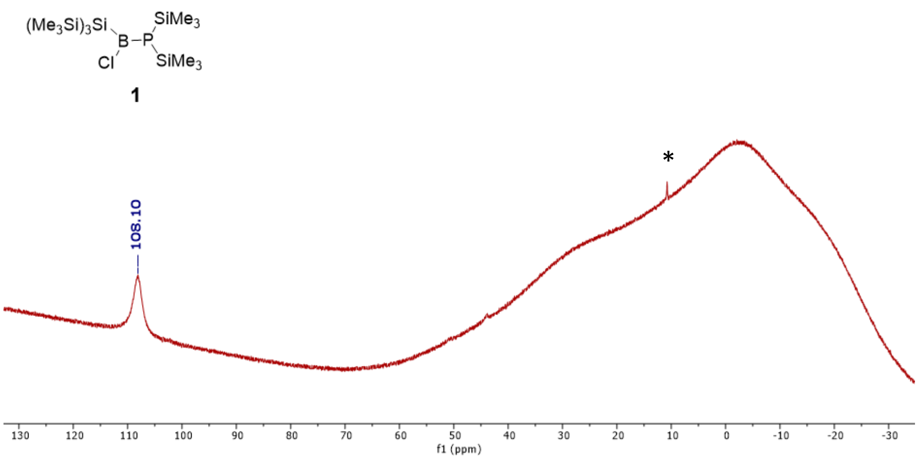

Figure S2. ^{11}B NMR (128.3 MHz, Hexane, 300 K) spectrum of compound **1**. Impurity (*).

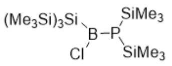

1

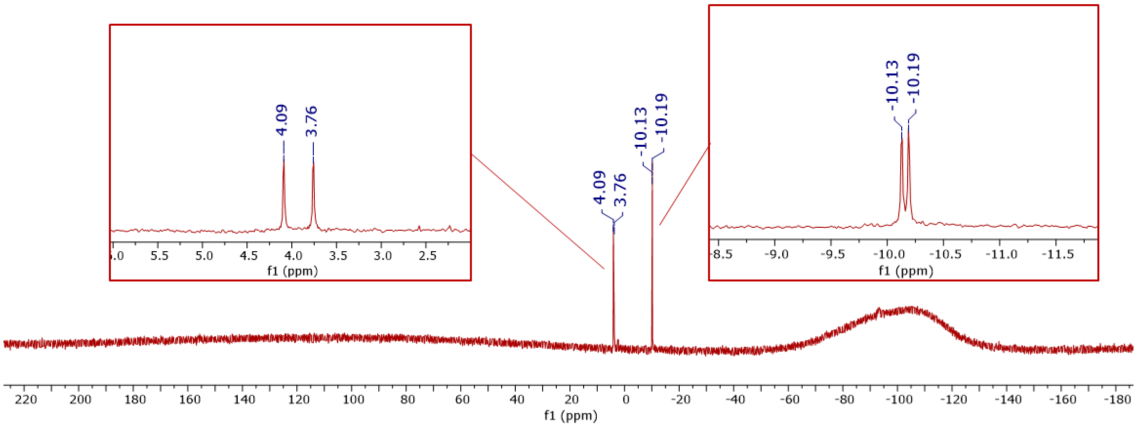

Figure S3: ^{29}Si{^{1}H} NMR (79.4 MHz, Hexane, 300 K) spectrum of compound **1**.

Compound 2: 3.32 g (10.09 mmol) of $(Me_3Si)_3SiBCl_2$ and 3.82 g (10.59 mmol, 1.05 eq) of $KP(SiMe_3)_2$ were taken together in a 250 mL Schlenk flask and 120 mL of *n*-hexane was added to it at room temperature. The mixture was stirred at room temperature for 2 hours at which point all of $(Me_3Si)_3SiBCl_2$ was consumed and formed $(Me_3Si)_3SiB(Cl)P(SiMe_3)_2$. The orange reaction mixture was filtered through a filter cannula and a hexane solution of 1,3-diisopropyl-4,5-dimethylimidazol-2-ylidene (1.82 g. 10.09 mmol) was added to the filtrate at room temperature. The resulting reaction mixture was stirred overnight at room temperature during which light yellow precipitates started forming. The next morning the reaction mixture was dried under vacuum to remove the solvent and any volatiles. The resulting orange sticky paste was washed twice with hexane (2*50 mL) at – 20°C to get compound **2** in pure form as pale yellow solid. **Yield:** 4.4 g (80%). **Melting Point**: Decomposes before melting (> 225°C). **^{1}H NMR** (400.13 MHz, C_6D_6, 300 K): 0.19 (d, $^{4}J_{P,H}$ = 2.7 Hz, 9H, **P-SiMe$_3$**), 0.48 (s, 27H, B-**Si(SiMe$_3$)$_3$**), 1.14 (d, $^{3}J_{H,H}$ = 7.2 Hz, 6H, NCH**Me$_2$**), 1.19 (d, $^{3}J_{H,H}$ = 6.8 Hz, 6H, NCH**Me$_2$**), 1.53 (s, **C^{4,5}Me of NHC**), 4.69 (sept, $^{3}J_{H,H}$ = 7.2 Hz, 2H, N**CH**Me$_2$). **^{13}C{^{1}H} NMR** (100.61 MHz, C_6D_6, 300 K): 3.1 (d, $^{4}J_{C,P}$ = 3 Hz, B-**Si(SiMe$_3$)$_3$**), 3.8 (d, $^{2}J_{C,P}$ = 7.5 Hz, **P-SiMe$_3$**), 9.7 (s, C^{4,5}-**Me**), 20.8 & 22.0 (s, NCH**Me$_2$**), 50.9 (s, N**CH**Me$_2$), 124.2 (s, **C^{4,5} of NHC**), 158.1 (bs, C^{2} **of NHC**). **^{31}P NMR** (400.13 MHz, C_6D_6, 300 K): 158.4 (s). **^{29}Si{^{1}H} NMR** (59.6 MHz, C_6D_6, 300 K): - 12.2 (d, $^{4}J_{Si,P}$ = 15.5 Hz, B-Si**(SiMe$_3$)$_3$**), - 4.6 (d, $^{2}J_{C,P}$ = 92.3 Hz, **P-SiMe$_3$**). **^{11}B{^{1}H} NMR** (128.38 MHz, C_6D_6, 300 K): 57.6. **UV/vis (Toluene):** λ_{max} (ε) = 336 nm (836 ± 61 $Lmol^{-1}cm^{-1}$). **IR (cm^{-1}): ṽ** = 624 (m), 684 (m), 739 (m), 917 (w), 1134 (w), 1166 (w), 1221 (m), 1329 (w), 1369 (s), 1377 (s), 1550 (w), 1633 (m), 2922 (s), 2952 (s). **Elemental analysis (%):** calcd for **2** : C:

50.88, H: 10.40, N: 5.16; found: C: 50.03, H: 9.31, N: 5.01. Compound **2** consistently analysed low for carbon over repeated analyses.

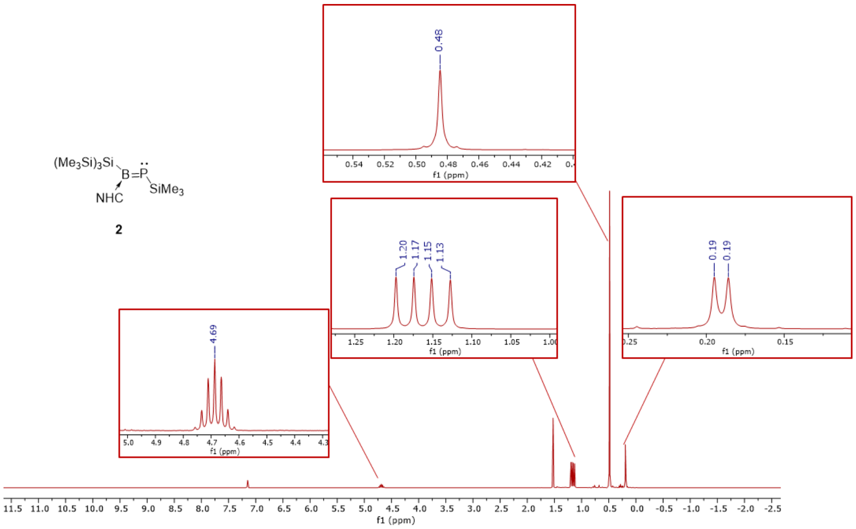

Figure S4. ^{1}H NMR (400.1 MHz, C_6D_6, 300 K) spectrum of compound **2**.

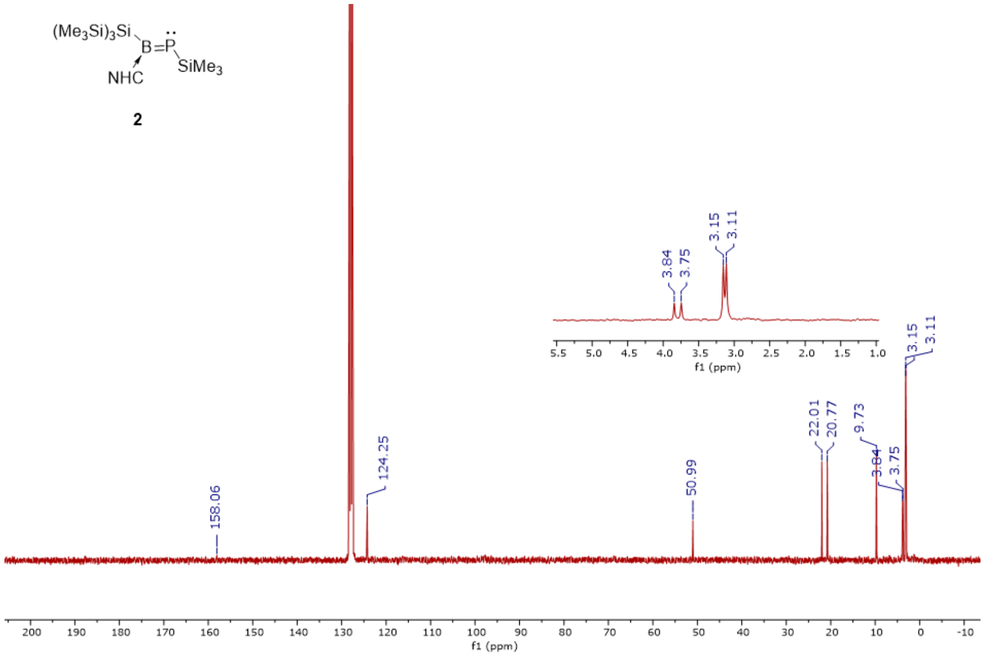

Figure S5. $^{13}C\{^{1}H\}$ NMR (100.6 MHz, C_6D_6, 300 K) spectrum of compound **2**.

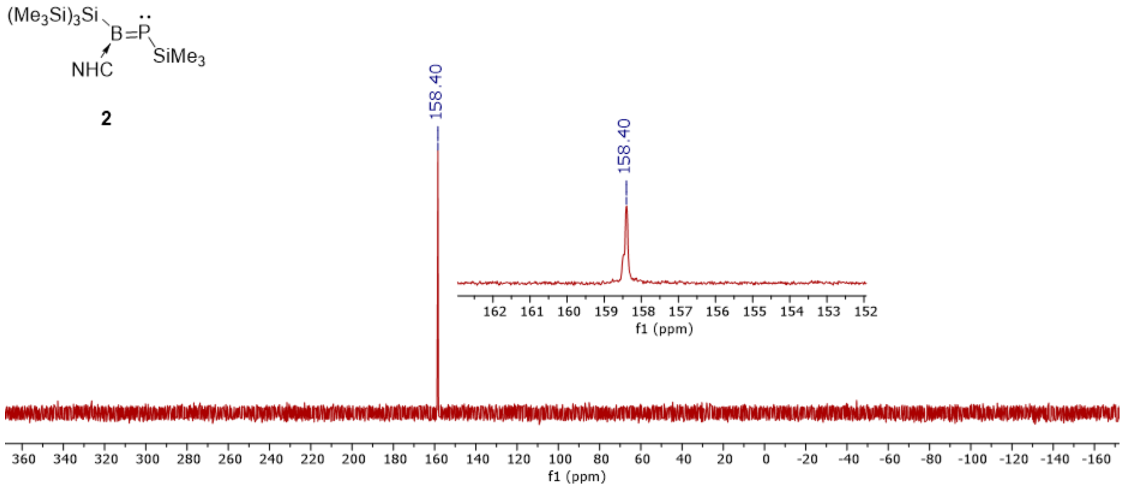

Figure S6. ^{31}P NMR (161.9 MHz, C_6D_6, 300 K) spectrum of compound **2**.

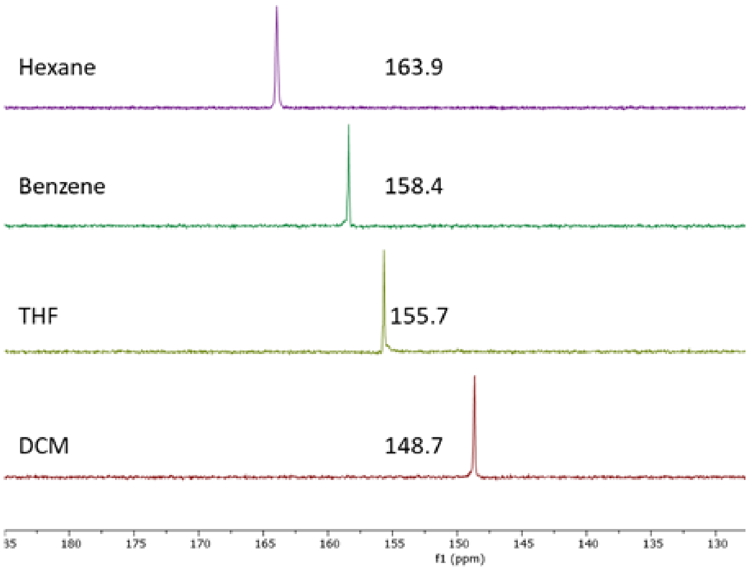

Figure S7. ^{31}P NMR (161.9 MHz, 300 K) spectra of compound **2** in different solvents.

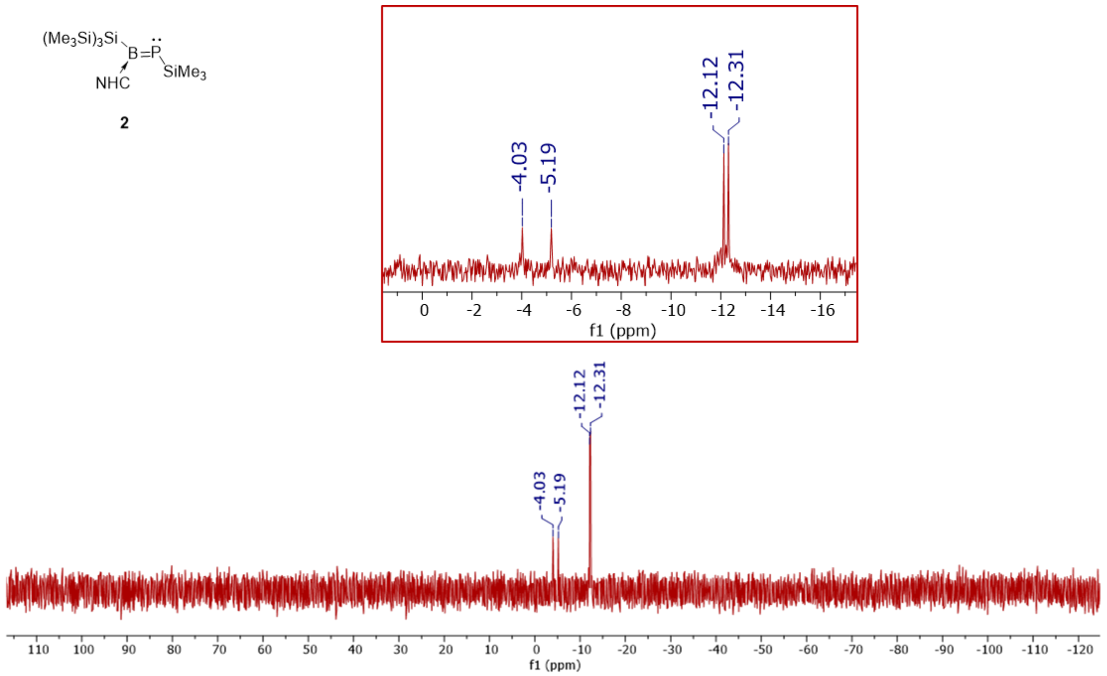

Figure S8: $^{29}Si\{^{1}H\}$ NMR (79.4 MHz, C_6D_6, 300 K) spectrum of compound **2**.

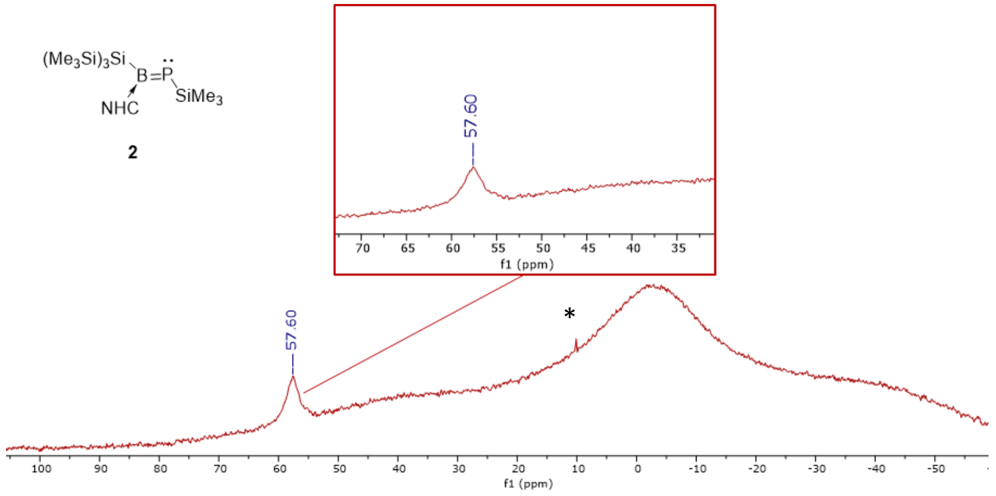

Figure S9: $^{11}B\{^{1}H\}$ NMR (128.3 MHz, C_6D_6, 300 K) spectrum of compound **2**. Impurity (*).

Compound 3: Compound **2** (1.0 g, 1.84 mmol) and $TipPCl_2$ (Tip = 2,4,6-$i$$Pr_3C_6H_2$) (0.56 g, 1.84 mmol) were taken in a Schlenk flask and 15 mL of THF was added to it at room temperature. The resulting mixture was stirred at this condition for 30 min and then dried under vacuum to remove all volatiles. The bright yellow residue was washed with 5 mL hexane at -20°C to get compound **3** as bright yellow solid in pure form. It was then dried under vacuum. **Yield:** 1.02 g (75%). **Melting Point:** 146°C. **^{1}H NMR** (400.13 MHz, C_6D_6, 300 K): 0.42 (s, 27H, B-**Si(SiMe$_3$)$_3$**), 0.93 (d, $^3J_{H,H}$ = 6.9 Hz, 3H, NCH**Me$_2$**), 1.06 (d, $^3J_{H,H}$ = 6.9 Hz, 3H, NCH**Me$_2$**), 1.18 (d, $^3J_{H,H}$ = 6.9 Hz, 3H, NCH**Me$_2$**), 1.18 (d, $^3J_{H,H}$ = 7.0 Hz, 3H, CH**Me$_2$ of Tip**), 1.19 (d, $^3J_{H,H}$ = 6.9 Hz, 3H, NCH**Me$_2$**), 1.39 (br, 6H, CH**Me$_2$ of Tip**), 1.53 (br, 6H, CH**Me$_2$ of Tip**), 1.58 & 1.61 (s, **C^{4,5}Me of NHC**), 1.62 (d, $^3J_{H,H}$ = 7.0 Hz, 3H, CH**Me$_2$ of Tip**), 2.75 (sept, $^3J_{H,H}$ = 7.0 Hz, 1H, **CH**Me$_2$), 4.43 (sept, $^3J_{H,H}$ = 7.0 Hz, 1H, **CH**Me$_2$), 4.55 (br, 2H, **CH**Me$_2$), 4.77 (sept, $^3J_{H,H}$ = 7.0 Hz, 1H, **CH**Me$_2$), 7.17 (s, 2H, **CH** of Tip). **^{13}C{^{1}H} NMR** (100.61 MHz, C_6D_6, 300 K): 3.16 (d, $^4J_{C,P}$ = 3.2 Hz, B-**Si(SiMe$_3$)$_3$**), 9.9 & 10.1 (s, C^{4,5}-**Me**), 21.3 (d, $^4J_{C,P}$ = 6.5 Hz, CH**Me$_2$** of Tip), 21.8 (d, $^4J_{C,P}$ = 7.5 Hz, CH**Me$_2$** of Tip), 22.1 (s, CH**Me$_2$** of NHC), 22.6 (s, CH**Me$_2$** of NHC), 23.9 (s, CH**Me$_2$** of Tip), 24.5 (br s, CH**Me$_2$** of NHC), 26.0 (s, CH**Me$_2$** of NHC), 32.2 (d, $^4J_{C,P}$ = 8.2 Hz, **CH**Me$_2$ of Tip), 32.5 (d, $^4J_{C,P}$ = 8.5 Hz, **CH**Me$_2$ of Tip), 34.6 (s, **CH**Me$_2$ of Tip), 51.7 & 51.9 (s, N**CH**Me$_2$), 124.9 & 125.2 (s, **C^{4,5} of NHC**), 128.1 (s, **CH** of Tip ring), 132.3 (s, C of Tip ring), 137.1 (d, $^1J_{C,P}$ = 57.2 Hz), 141.8 Hz (s, C of Tip ring), 150.6 (s, C^2 **of NHC**). **^{31}P NMR** (400.13 MHz, C_6D_6, 300 K): 123.5 (d, $^1J_{P,P}$ = 331 Hz, sp^3-P) & 191.3 (d, $^1J_{P,P}$ = 331 Hz, sp^2-P). **^{29}Si{^{1}H} NMR** (59.6 MHz, C_6D_6, 300 K): - 11.4 (d, $^4J_{Si,P}$ = 13.5 Hz, B-Si**(SiMe$_3$)$_3$**. **^{11}B{^{1}H} NMR** (128.38 MHz, C_6D_6, 300 K): 64.7. **UV/vis (Toluene):** λmax (ε) = 361 nm (1024 ± 42 Lmol^{-1}cm^{-1}). **IR (cm^{-1}):** $\tilde{\nu}$ = 624 (m), 684 (m), 835 (s), 874 (w), 1106 (w), 1134 (w), 1165 (w), 1225 (w), 1241 (s), 1376 (s), 1408 (w), 1598 (w), 1630 (w), 2847 (s), 2952 (s), 2969 (s). **Elemental analysis (%):** calcd for **3** : C: 56.85, H: 9.54, N: 3.79; found: C: 56.36, H: 8.80, N: 3.90.

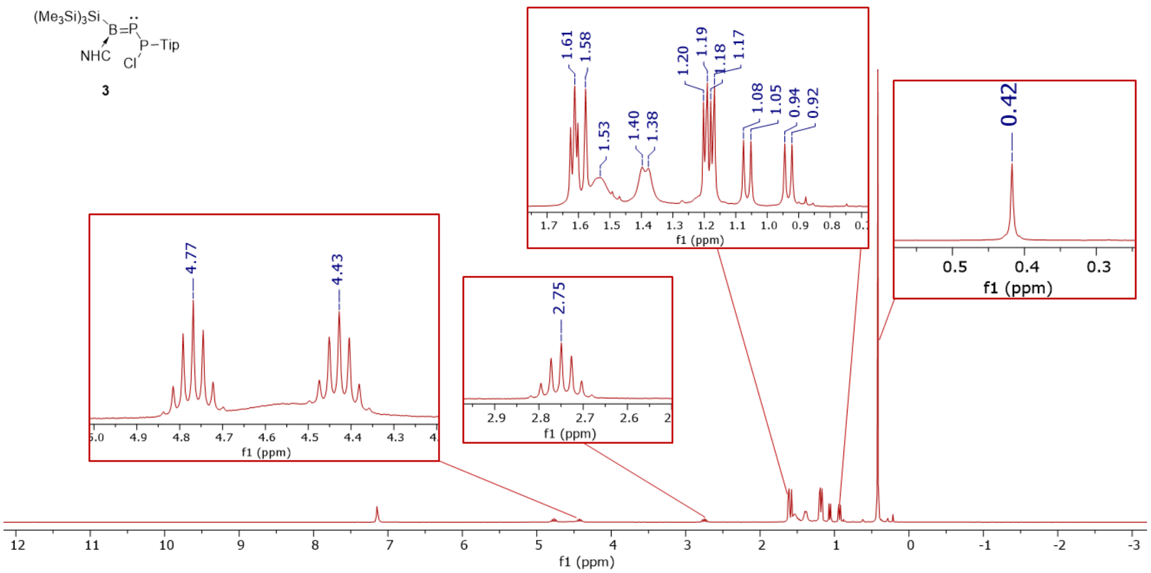

Figure S10. ^{1}H NMR (400.1 MHz, C_6D_6, 300 K) spectrum of compound **3.**

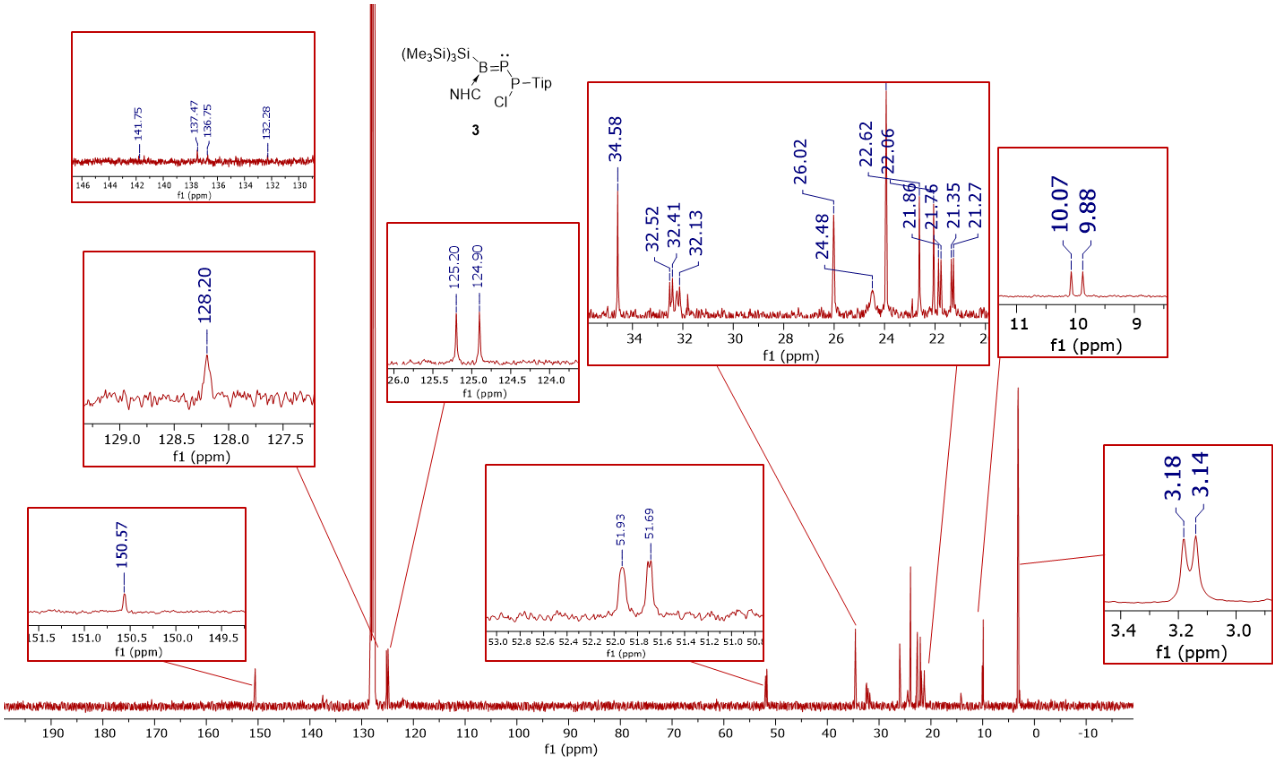

Figure S11. $^{13}C\{^{1}H\}$ NMR (100.6 MHz, C_6D_6, 300 K) spectrum of compound **3**.

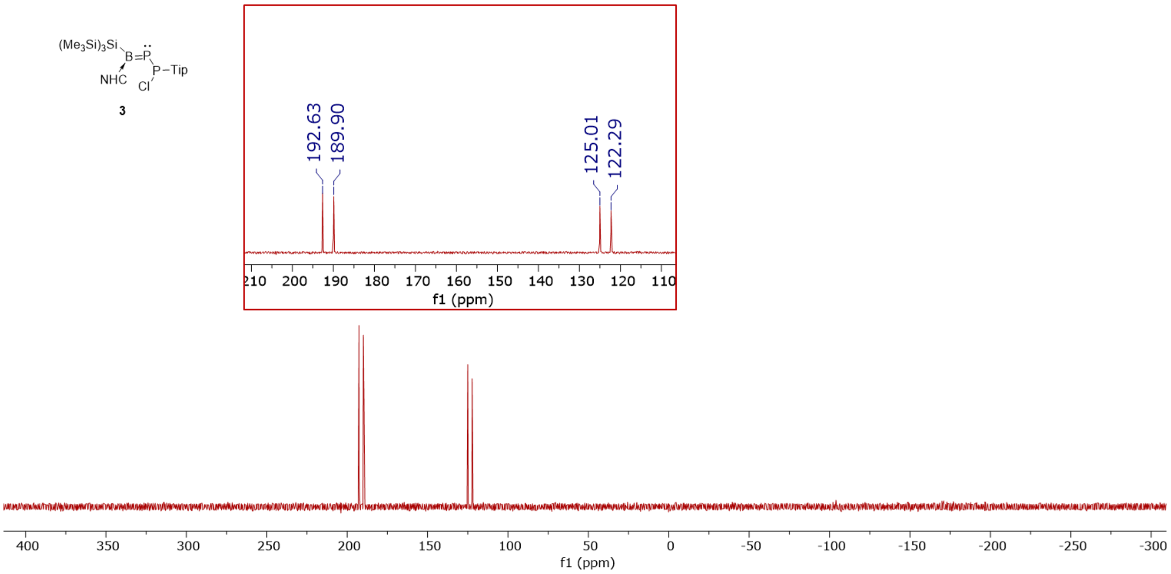

Figure S12. ^{31}P NMR (161.9 MHz, C_6D_6, 300 K) spectrum of compound **3**.

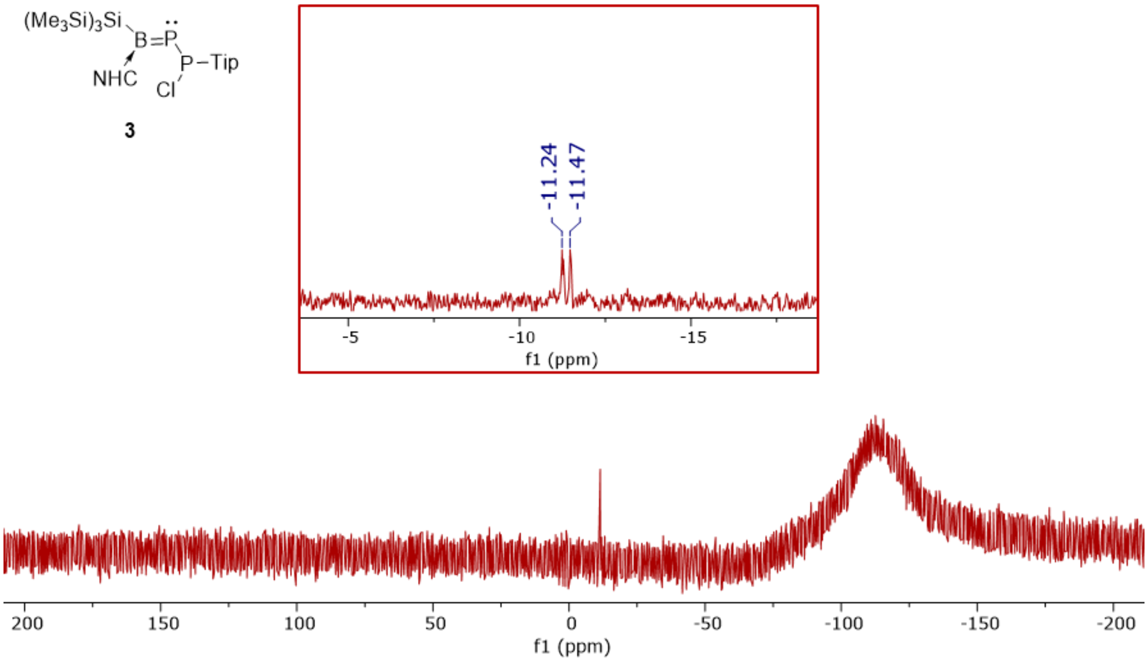

Figure S13: $^{29}Si\{^1H\}$ NMR (79.4 MHz, C_6D_6, 300 K) spectrum of compound **3**.

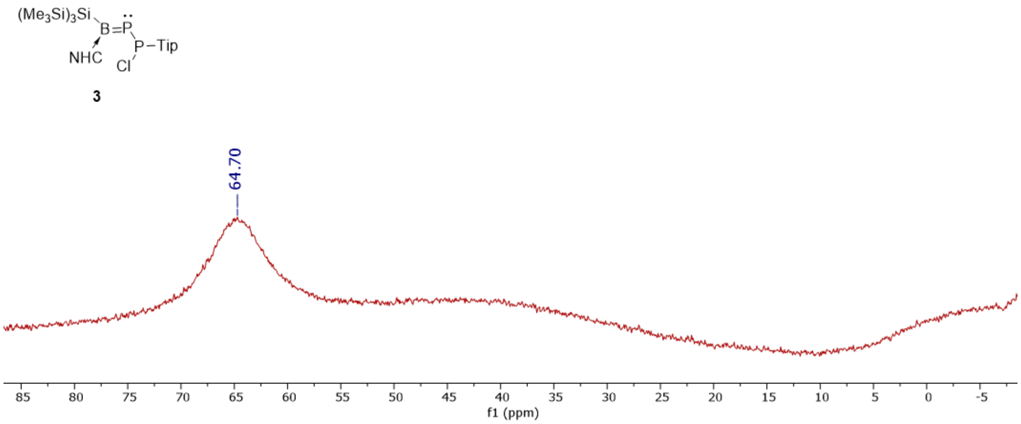

Figure S14: $^{11}B\{^1H\}$ NMR (128.3 MHz, C_6D_6, 300 K) spectrum of compound **3**.

Compound 4: Compound **2** (1.0 g, 1.84 mmol) and $PhSiCl_3$ (1.48 mL, 9.21 mmol) were taken in a Schlenk flask and 15 mL of Toluene was added to it at room temperature. The resulting mixture was stirred at this condition for 3 hours and then dried under vacuum to remove all volatiles. The pale yellow residue was dissolved in 25 mL hexane and kept at -20°C for crystallization to get compound **4** as pale yellow solid in pure form. The pale yellow solid was then collected and dried under vacuum. **Yield:** 0.594 g (50%). **Melting Point:** 192°C. **^{1}H NMR** (400.13 MHz, C_6D_6, 300 K): 0.25 (s, 27H, B-**Si(SiMe$_3$)$_3$**), 0.92 (d, $^3J_{H,H}$ = 7.1 Hz, 6H, NCH**Me$_2$**), 1.05 (d, $^3J_{H,H}$ = 6.9 Hz, 6H, NCH**Me$_2$**), 1.36 (s, 6H, **C^{4,5}Me of NHC**), 4.33 (sept, $^3J_{H,H}$ = 7.1 Hz, 2H, N**CH**Me$_2$), 6.86-6.99 (m, 3H, Ph ring protons), 8.03 (dd, 2H, $^3J_{H,H}$ = 8.2 Hz, $^4J_{H,H}$= 1.5 Hz, ortho H of Ph ring). **^{13}C{^{1}H} NMR** (100.61 MHz, C_6D_6, 300 K): 0.42 (d, $^4J_{C,P}$ = 3.1 Hz, B-**Si(SiMe$_3$)$_3$**), 7.1 (s, C^{4,5}-**Me**), 18.4 & 19.6 (s, NCH**Me$_2$**), 49.0 & 49.1 (s, N**CH**Me$_2$), 122.9 (s, **CH** of Ph ring), 125.6 (s, C^{4,5} of NHC), 127.6 (s, **CH** of Ph ring), 131.7 (d, $^3J_{C,P}$ = 3.6 Hz, *o*-CH of Ph ring), 138.4 (d, $^2J_{C,P}$ = 5.2 Hz, *quart* **C** *of* Ph ring), 155.6 (br s, **C^2** of NHC). **^{31}P NMR** (400.13 MHz, C_6D_6, 300 K): 92.2 **^{29}Si{^{1}H} NMR** (59.6 MHz, C_6D_6, 300 K): -11.5 (d, $^4J_{Si,P}$ = 15.4 Hz, B-Si**(SiMe$_3$)$_3$**, 19.1 (d, $^1J_{Si,P}$ = 189.6 Hz, B-$SiCl_2$Ph. **^{11}B{^{1}H} NMR** (128.38 MHz, C_6D_6, 300 K): 66.8 **UV/vis (Toluene):** λ_{max} (ε) = 338 nm (770 ± 63 $Lmol^{-1}cm^{-1}$). **IR (cm^{-1}): ṽ** = 624 (m), 684 (m), 741 (w), 832 (s), 1107 (m), 1241 (s), 1370 (w), 1389 (w), 1407 (w), 1429 (w), 2920 (s), 2929 (s). **Elemental analysis (%):** calcd for **4** : C: 48.35, H: 8.12, N: 4.34; found: C: 47.99, H: 7.98, N: 4.17.

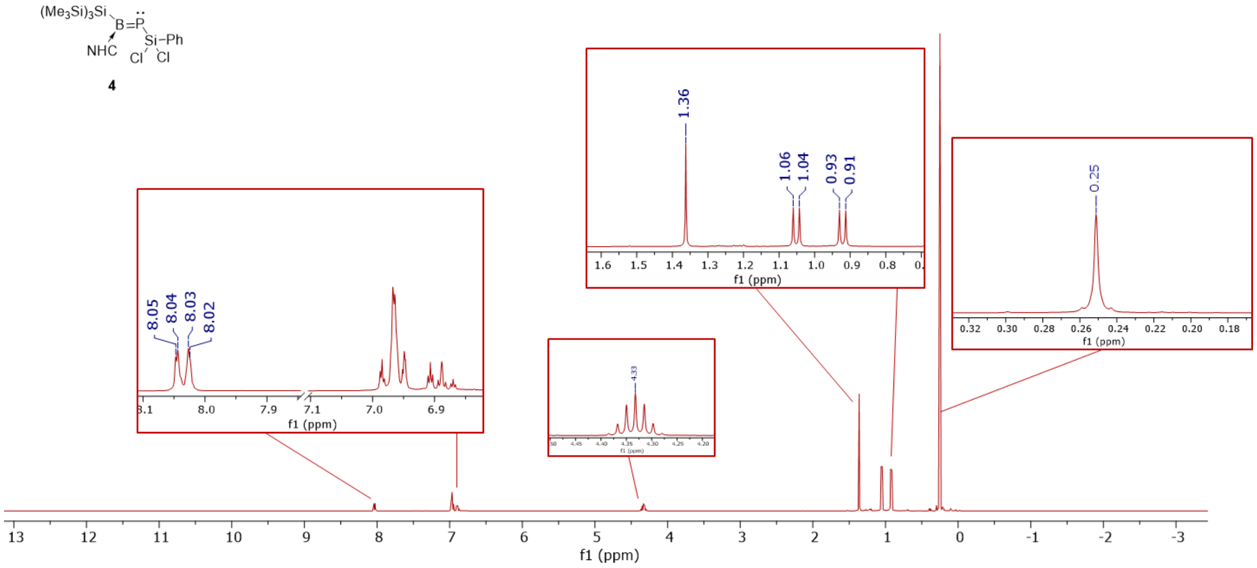

Figure S15. ^{1}H NMR (400.1 MHz, C_6D_6, 300 K) spectrum of compound **4.**

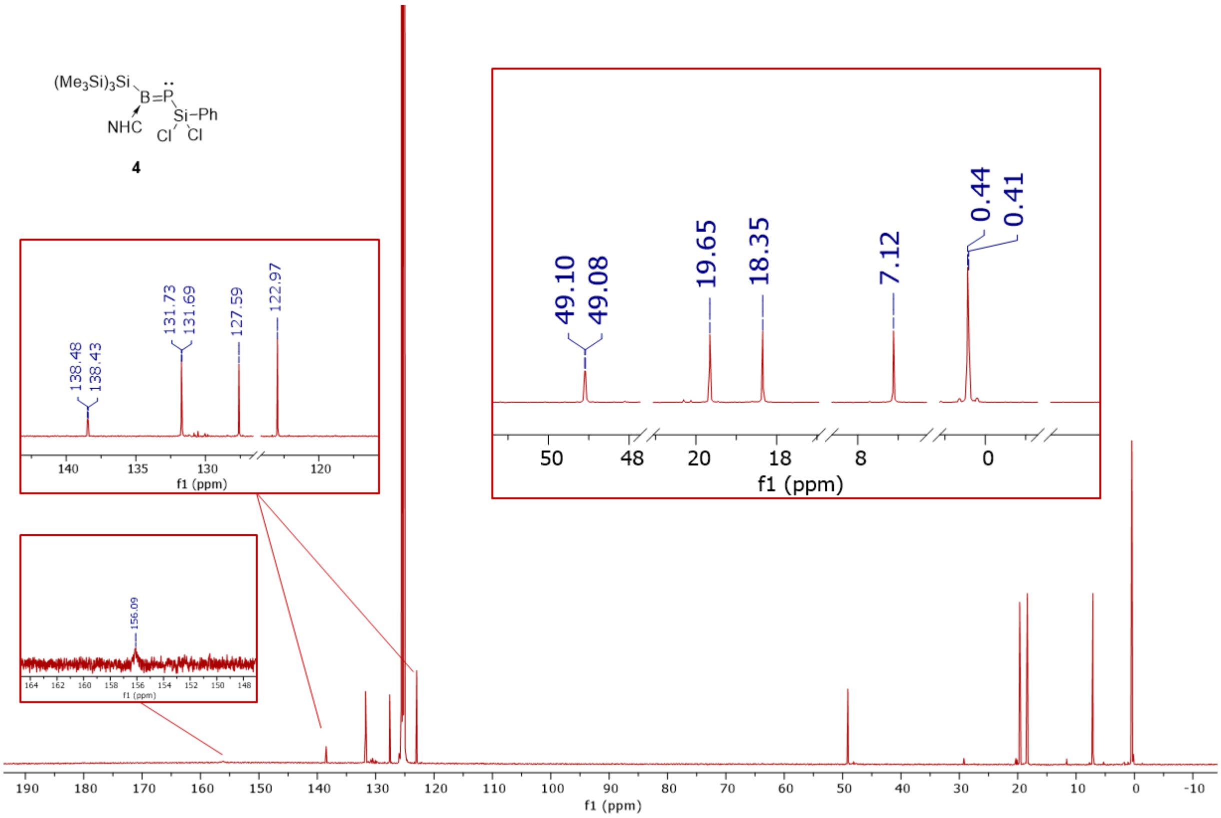

Figure S16. $^{13}C\{^{1}H\}$ NMR (100.6 MHz, C_6D_6, 300 K) spectrum of compound **4**.

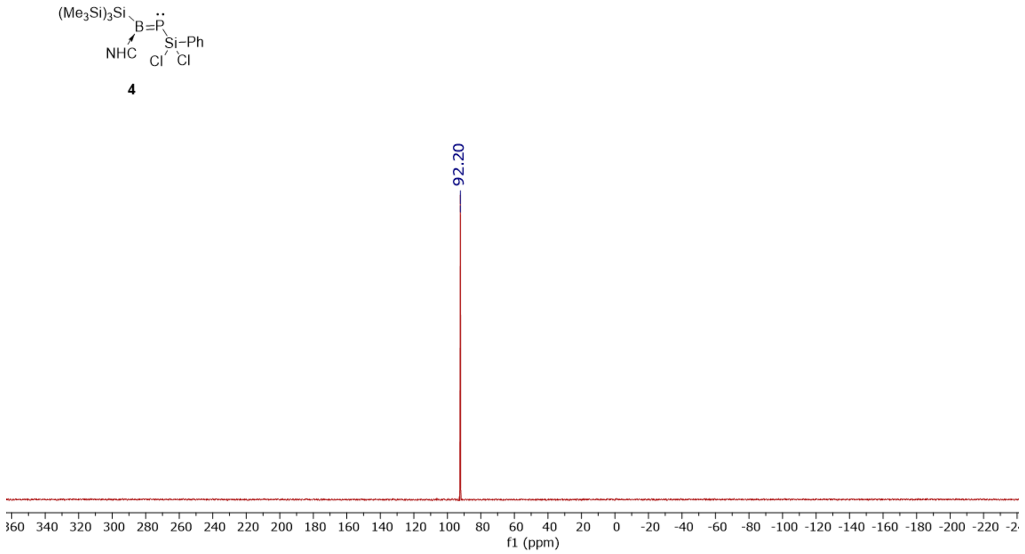

Figure S17. ^{31}P NMR (161.9 MHz, C_6D_6, 300 K) spectrum of compound **4**.

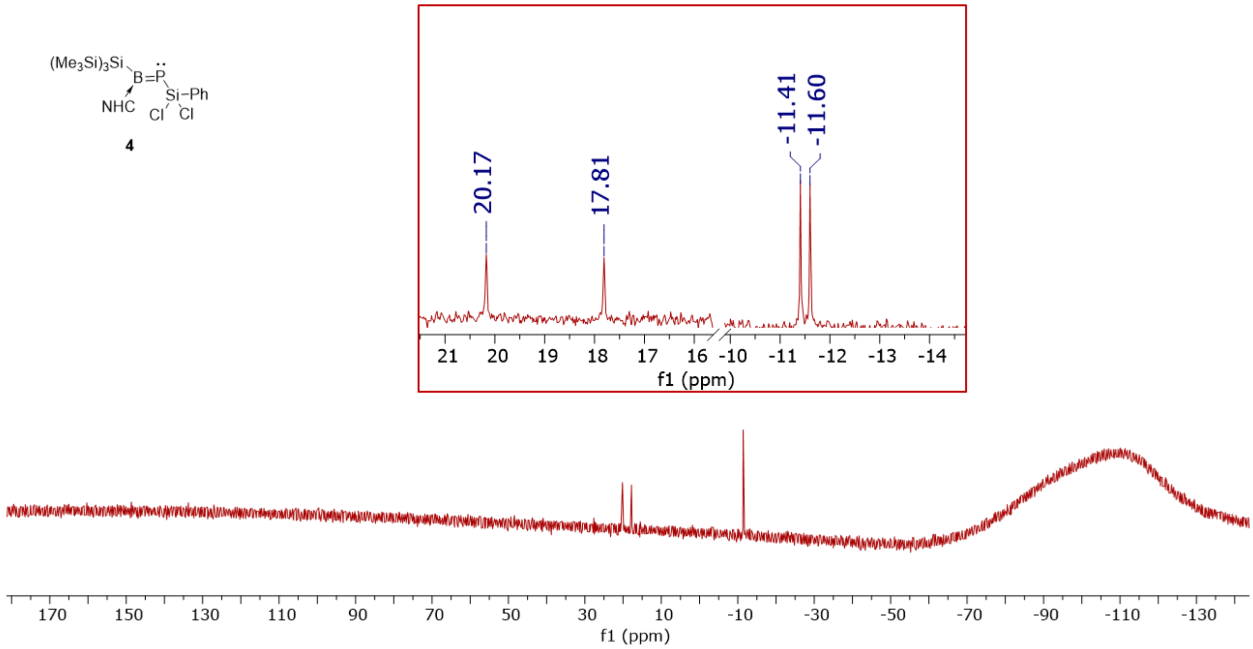

Figure S18: $^{29}Si\{^{1}H\}$ NMR (79.4 MHz, C_6D_6, 300 K) spectrum of compound **4**.

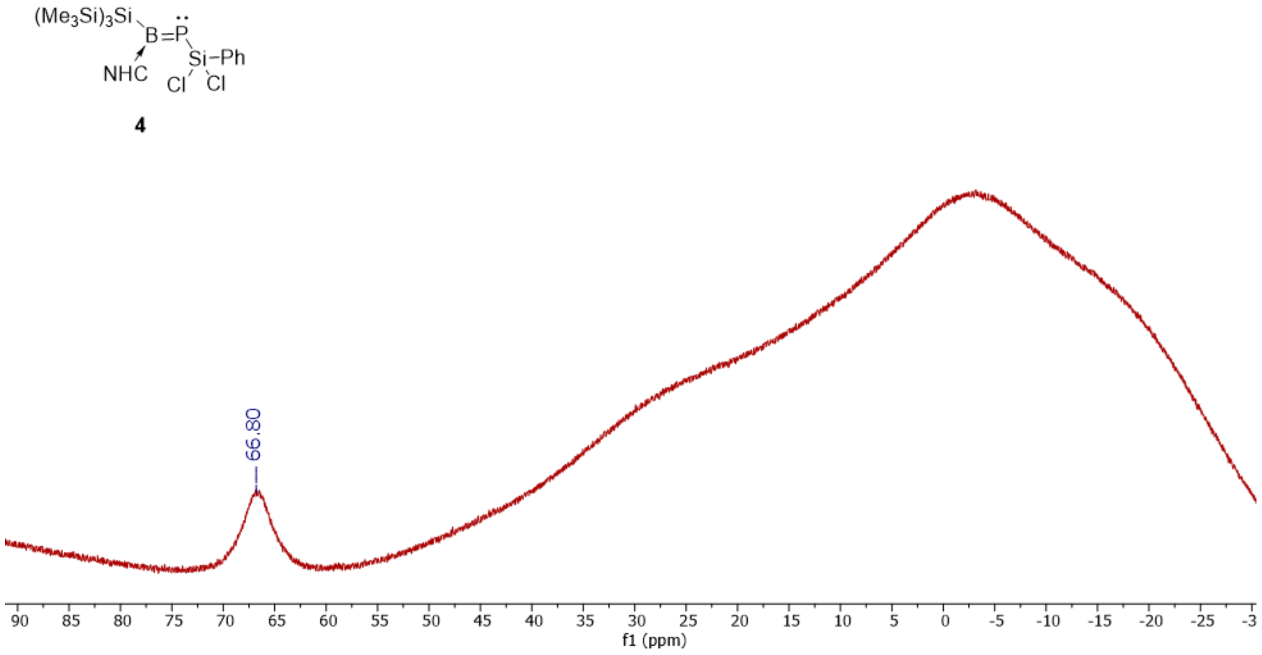

Figure S19: $^{11}B\{^{1}H\}$ NMR (128.3 MHz, C_6D_6, 300 K) spectrum of compound **4**.

Compound 5: Compound **2** (1.0 g, 1.84 mmol) and $GaCl_3$ (0.323 g, 1.84 mmol) were taken in a Schlenk flask and 25 mL of Toluene was added to it at room temperature. The resulting mixture was stirred at this condition for a week during which loads of bright yellow solids formed. Then the reaction mixture was dried under vacuum to remove all volatiles. The bright yellow residue was washed with 10 mL toluene and then with 10 ml hexane at room temperature to get remove any impurities and compound **5** as bright yellow solid in pure form. It was then dried under vacuum. **Yield:** 0.79 g (70%). **Melting Point:** Decomposes before melting (> 190°C). **^{1}H-NMR** (400.13 MHz, $CDCl_3$, 300 K): 0.21 (s, 27H, B-**Si(SiMe$_3$)$_3$**), 1.49 (d, $^{3}J_{H,H}$ = 6.9 Hz, 6H, NCH**Me$_2$**), 1.62 (d, $^{3}J_{H,H}$ = 6.7 Hz, 6H, NCH**Me$_2$**), 2.26 (s, 6H, **C^{4,5}Me of NHC**), 4.36 (sept, $^{3}J_{H,H}$ = 6.9 Hz, 2H, N**CH**Me$_2$). **^{13}C{^{1}H} NMR** (100.61 MHz, $CDCl_3$, 300 K): 3.0 (s, B-**Si(SiMe$_3$)$_3$**), 10.7 (s, C^{4,5}-**Me**), 22.2 & 22.7 (s, NCH**Me$_2$**), 52.4 (s, N**CH**Me$_2$), 126.7 (s, **C^{4,5} of NHC**), signal for C2 of NHC could not be traced. **^{31}P NMR** (400.13 MHz, $CDCl_3$, 300 K): 88.9, **^{31}P NMR** (400.13 MHz, $CDCl_3$, 300 K): 115.1 **^{29}Si{^{1}H} NMR** (59.6 MHz, $CDCl_3$, 300 K): -10.4 **^{11}B{^{1}H} NMR** (128.38 MHz, THF, 300 K): 66.9. **UV/vis (Toluene):** λ_{max} (ε) = 382 nm (1918 ± 115 $Lmol^{-1}cm^{-1}$). **IR (cm^{-1}): ṽ** = 624 (m), 684 (m), 834 (s), 899 (m), 1111 (w), 1233 (m), 1241 (m), 1330 (w), 1375 (w), 1407 (s), 1629 (m), 2929 (s), 2936 (s). **Elemental analysis (%):** calcd for **5**: C: 39.36, H: 7.76, N: 4.59; found: C: 38.68, H: 7.11, N: 4.29. Compound **5** consistently analysed low for carbon over repeated analyses.

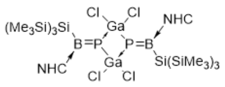

5

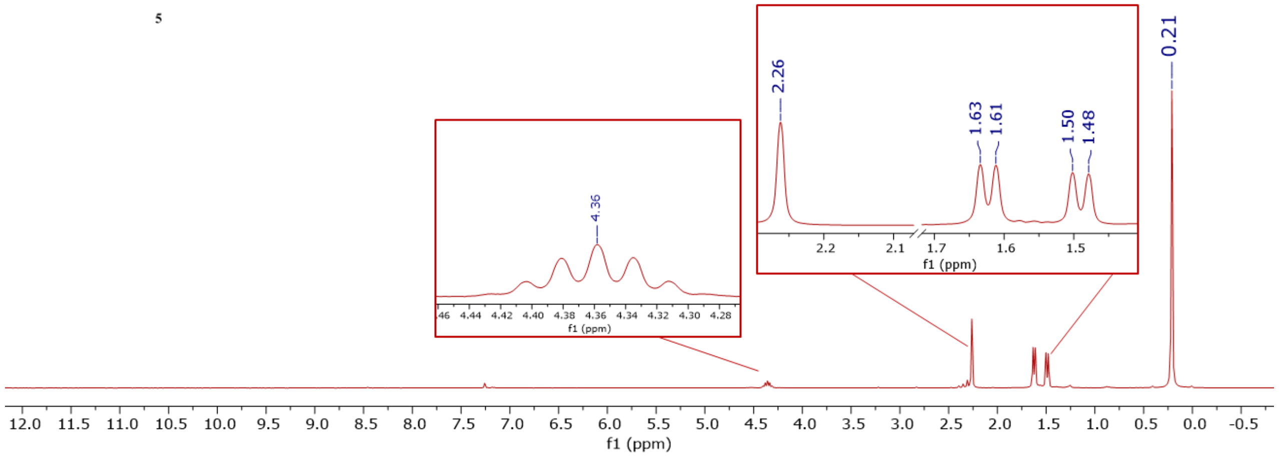

Figure S20. ^{1}H NMR (400.1 MHz, $CDCl_3$, 300 K) spectrum of compound **5.**

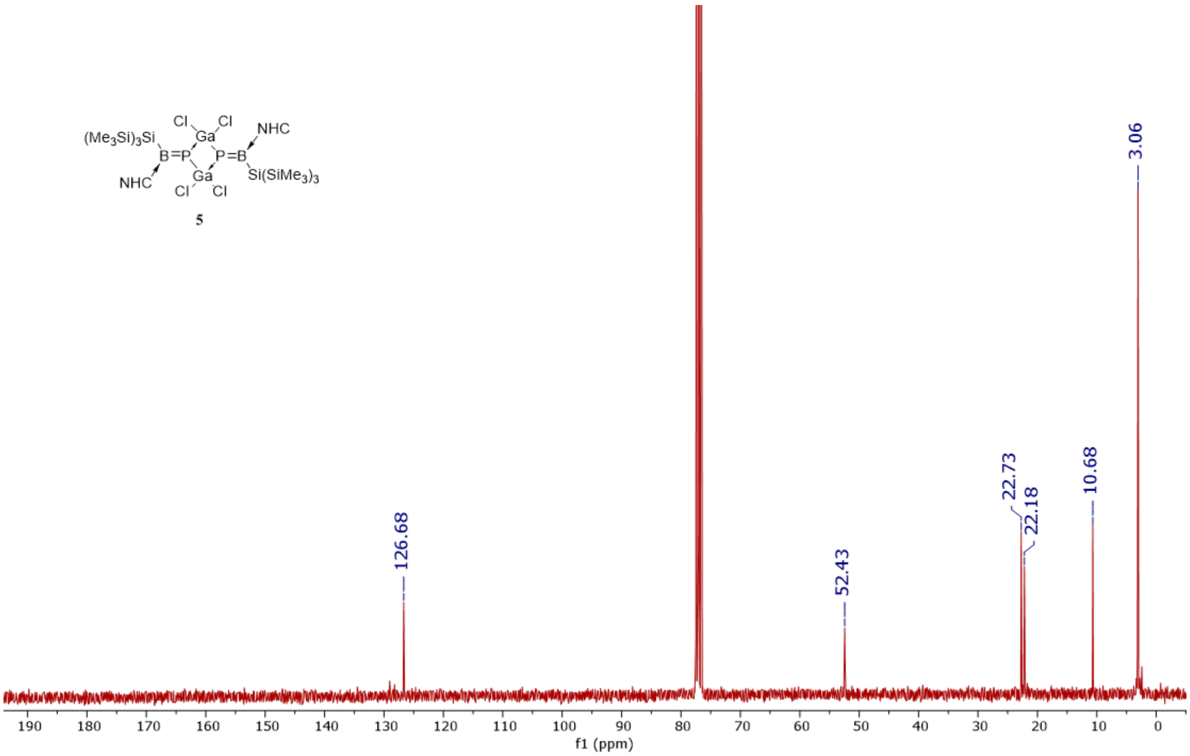

Figure S21. $^{13}C\{^{1}H\}$ NMR (100.6 MHz, $CDCl_3$, 300 K) spectrum of compound **5**.

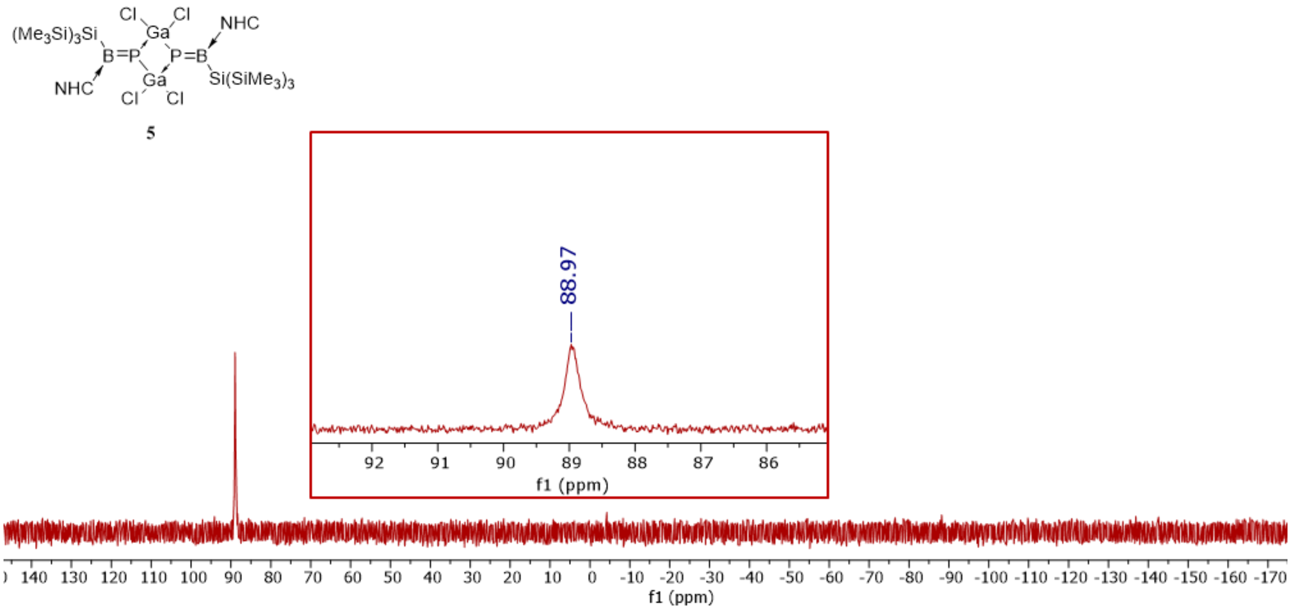

Figure S22. ^{31}P NMR (161.9 MHz, $CDCl_3$, 300 K) spectrum of compound **5**.

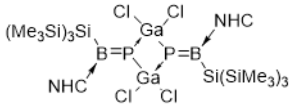

5

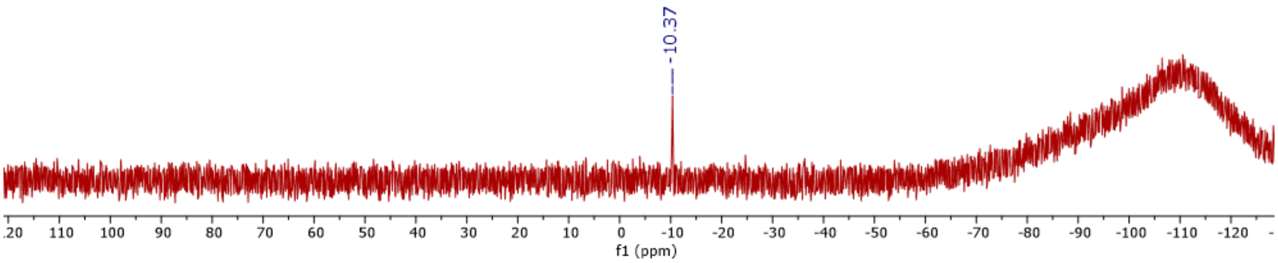

Figure S23: $^{29}Si\{^1H\}$ NMR (79.4 MHz, $CDCl_3$, 300 K) spectrum of compound **5**.

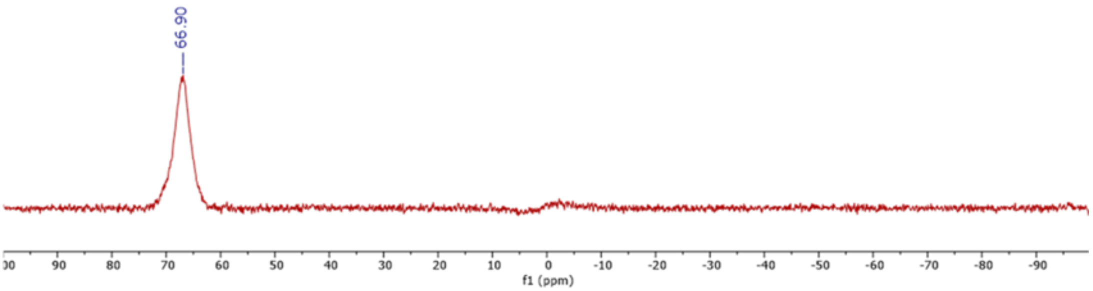

Figure S24: $^{11}B\{^1H\}$ NMR (128.3 MHz, THF, 300 K) spectrum of compound **5**.

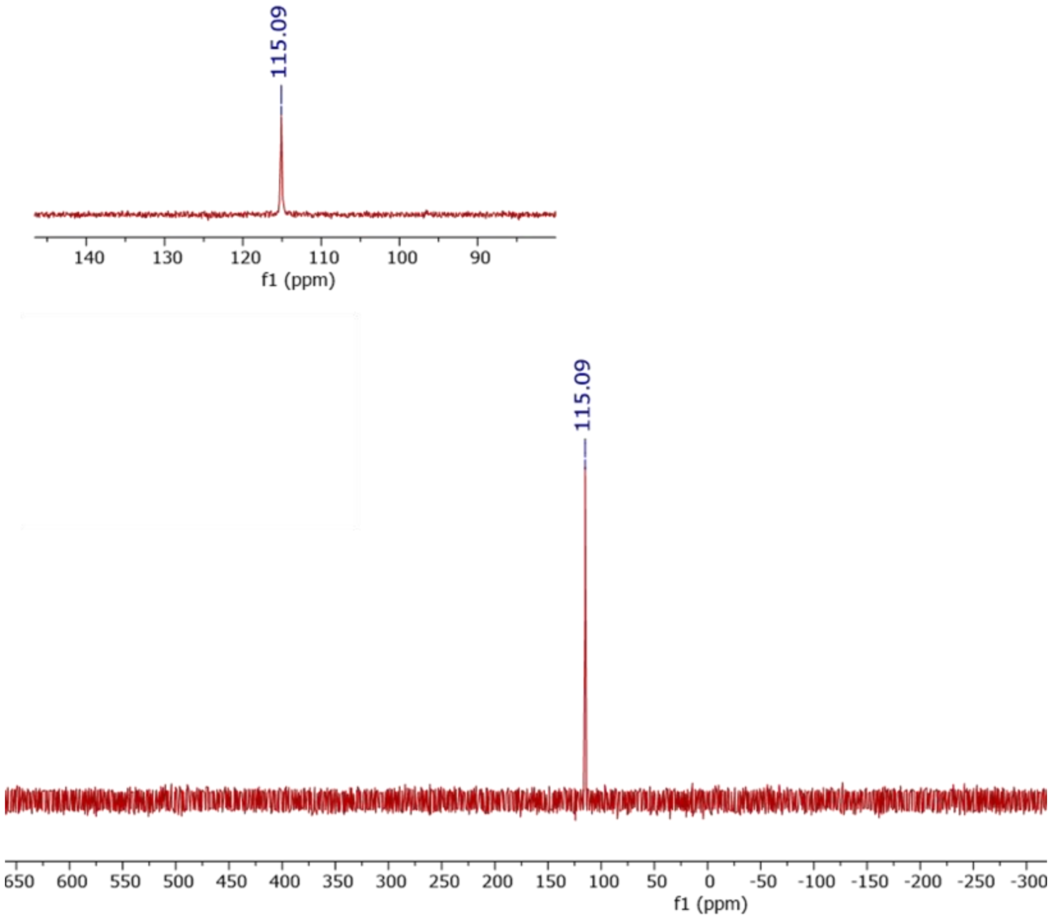

Figure S25. ^{31}P NMR (161.9 MHz, THF, 300 K) spectrum of compound **5**.

Compound 6: Compound **5** (0.15 g, 0.12 mmol) and 1,3-diisopropyl-4,5-dimethylimidazol-2-ylidene (49 mg, 0.27 mmol) were taken in a Schlenk flask and 10 mL of THF was added to it at room temperature. The resulting mixture was stirred at this condition for 30 min and then dried under vacuum to remove all volatiles. The pale yellow residue was washed with 5 mL hexane at room temperature to get compound **6** as pale yellow solid in pure form. It was then dried under vacuum. **Yield:** 0.15 g (80%). **Melting Point:** Decomposes before melting (> 207°C). **^{1}H NMR** (400.13 MHz, $CDCl_3$, 300 K): 0.18 (s, 27H, B-**Si(SiMe$_3$)$_3$**), 1.44 (d, $^{3}J_{H,H}$ = 7.1 Hz, 3H, NCH**Me$_2$**), 1.45 (d, $^{3}J_{H,H}$ = 7.1 Hz, 3H, NCH**Me$_2$**), 1.50 (d, $^{3}J_{H,H}$ = 6.8 Hz, 3H, NCH**Me$_2$**), 1.69 (d, $^{3}J_{H,H}$ = 6.8 Hz, 3H, NCH**Me$_2$**), 2.22 (s, 12H, **C^{4,5}Me of NHC**), 4.48-4.68 (m, 2H, N**CH**Me$_2$), 5.98-6.09 (m, 2H, N**CH**Me$_2$). **^{13}C{^{1}H} NMR** (100.61 MHz, $CDCl_3$, 300 K): 0.42 (d, $^{4}J_{C,P}$ = 2.9 Hz, B-**Si(SiMe$_3$)$_3$**), 8.1 & 8.2 (s, C^{4,5}-**Me**), 19.1 (s, NCH**Me$_2$**), 19.5 (s, NCH**Me$_2$**), 20.1 (s, NCH**Me$_2$**), 20.4 (s, NCH**Me$_2$**), 48.5 (s, N**CH**Me$_2$), 48.8 (s, N**CH**Me$_2$), 49.3 (s, N**CH**Me$_2$), 49.4 (s, N**CH**Me$_2$), 122.5 & 123.9 (s, **C^{4,5} of NHC**), 161.9 (s, C^{2} **of NHC**). **^{31}P NMR** (400.13 MHz, $CDCl_3$, 300 K): 144.6. **^{29}Si{^{1}H} NMR** (59.6 MHz, $CDCl_3$, 300 K): -12.1 (d, $^{4}J_{Si,P}$ = 15.2 Hz, B-Si**(SiMe$_3$)$_3$**. **^{11}B{^{1}H} NMR** (128.38 MHz, $CDCl_3$, 300 K): 60.1 **UV/vis (Toluene):** λ_{max} (ε) = 339 nm (858 ± 54 Lmol^{-1}cm^{-1}). **IR (cm^{-1}): ṽ** = 624 (m), 684 (m), 834 (s), 1076 (w), 1113 (w), 1255 (m), 1239 (m), 1369 (m), 1379 (s), 1408 (m), 1457 (m), 1464 (m), 2854 (s), 2916 (s), 2934 (s). **Elemental analysis (%):** calcd for **6** : C: 47.09, H: 8.54, N: 7.09; found: C: 47.37, H: 8.29, N: 6.91.

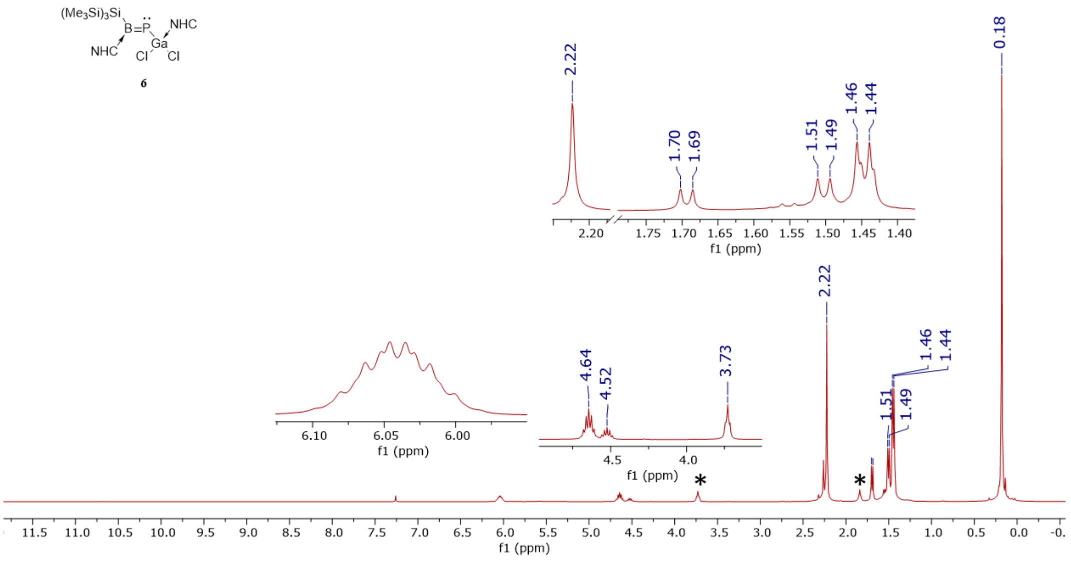

Figure S26. ^{1}H NMR (400.1 MHz, $CDCl_3$, 300 K) spectrum of compound **6. (*** THF solvent protons**)**

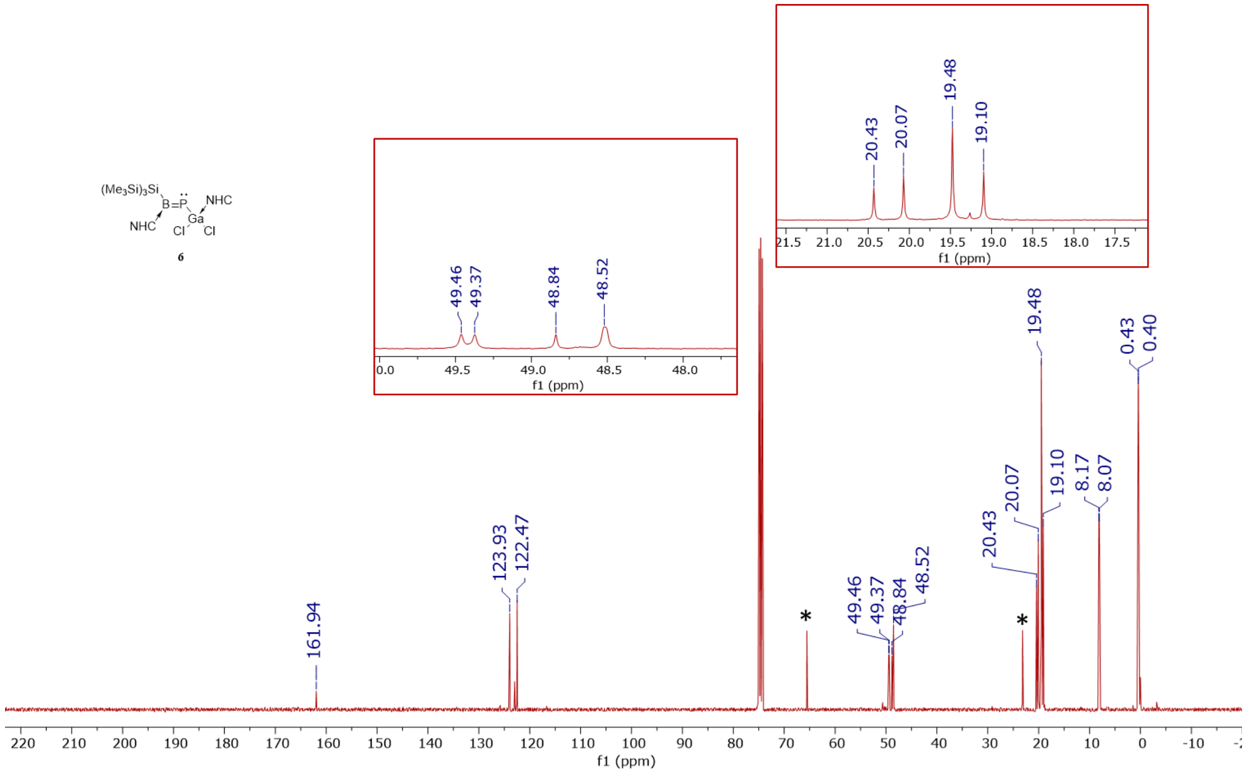

Figure S27. $^{13}C\{^{1}H\}$ NMR (100.6 MHz, $CDCl_3$, 300 K) spectrum of compound **6**. **(*** THF bound to the molecule**)**

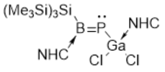

6

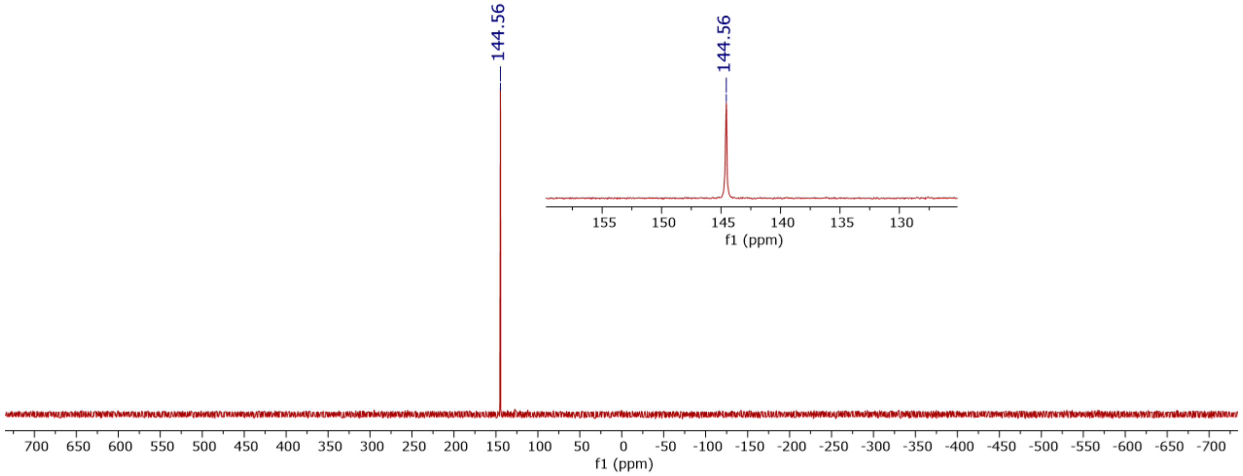

Figure S28. ^{31}P NMR (161.9 MHz, $CDCl_3$, 300 K) spectrum of compound **6**.

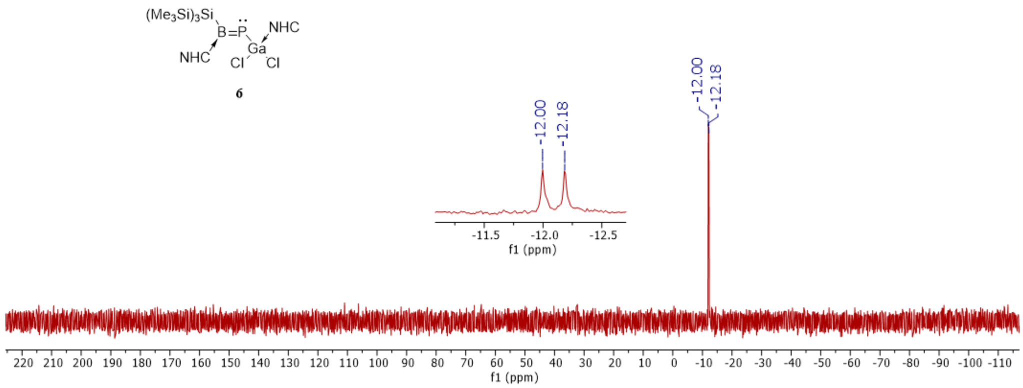

Figure S29: $^{29}Si\{^1H\}$ NMR (79.4 MHz, $CDCl_3$, 300 K) spectrum of compound **6**.

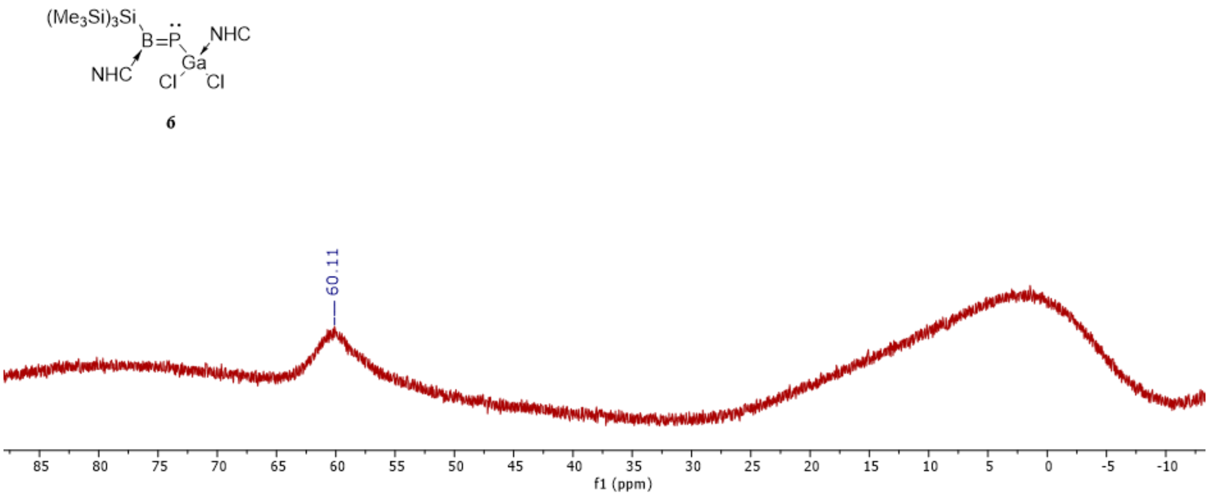

Figure S30: $^{11}B\{^1H\}$ NMR (128.3 MHz, $CDCl_3$, 300 K) spectrum of compound **6**.

UV-Visible Spectra

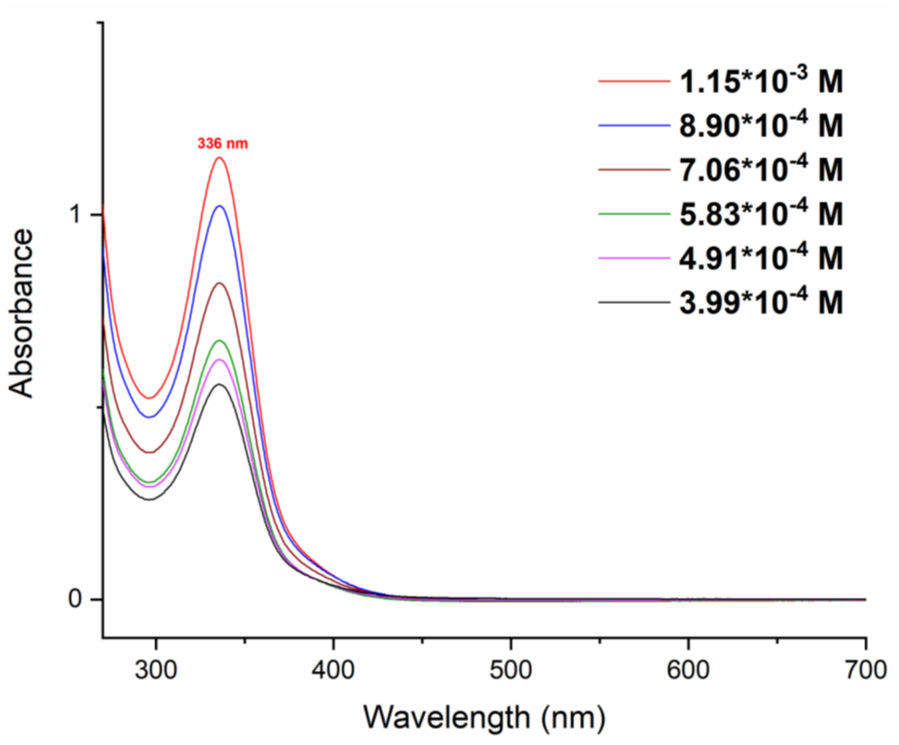

Figure S31: UV/vis spectra of compound **2** in THF at different concentrations.

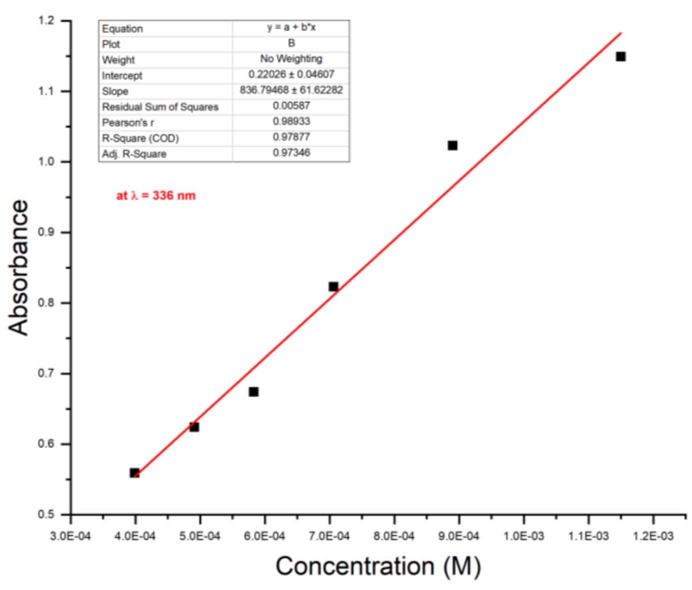

Figure S32: Linear regression of **2** in THF at 336 nm.

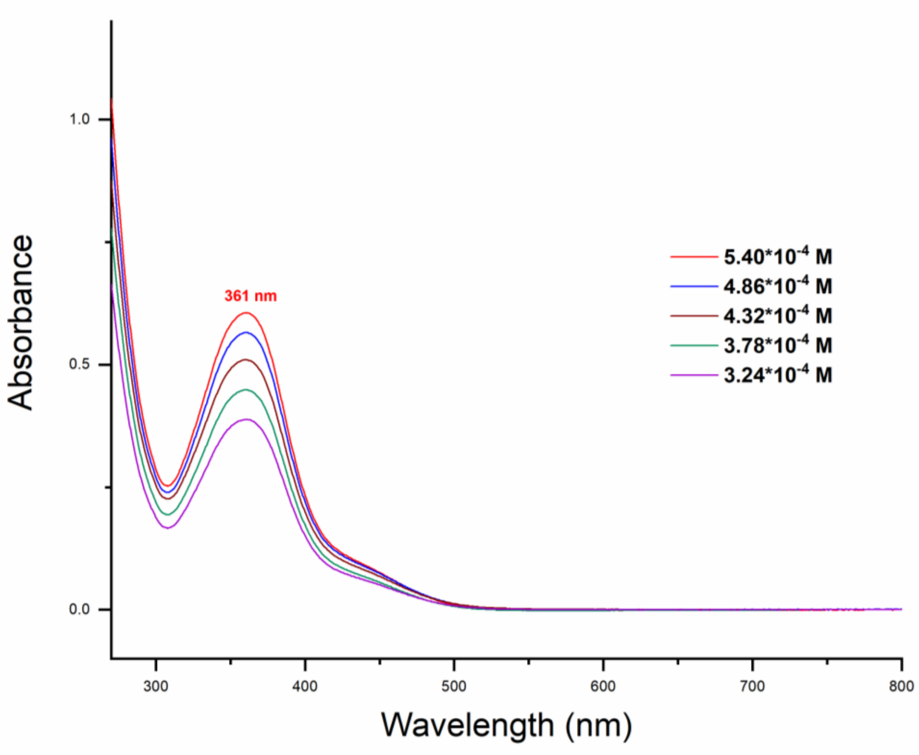

Figure S33: UV/vis spectra of compound **3** in THF at different concentrations.

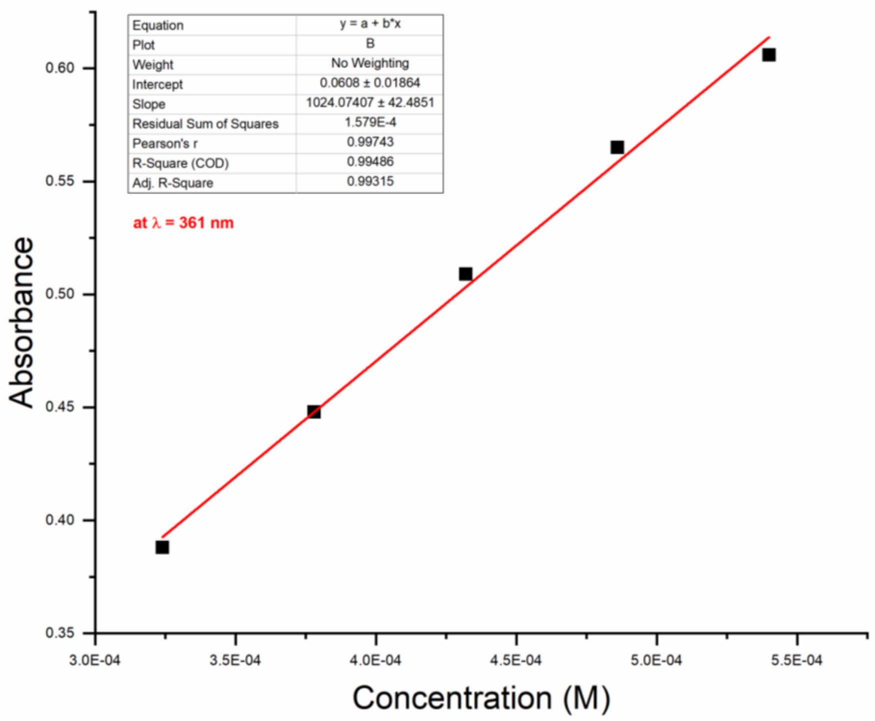

Figure S34: Linear regression of **3** in THF at 361 nm.

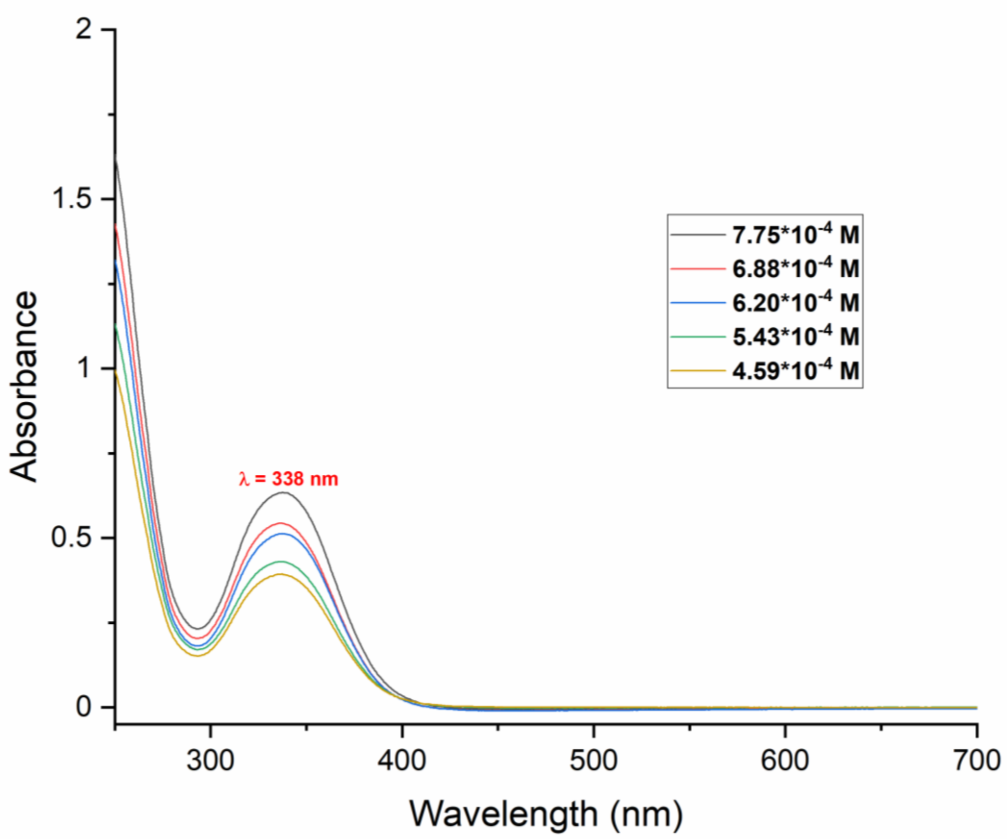

Figure S35: UV/vis spectra of compound **4** in THF at different concentrations.

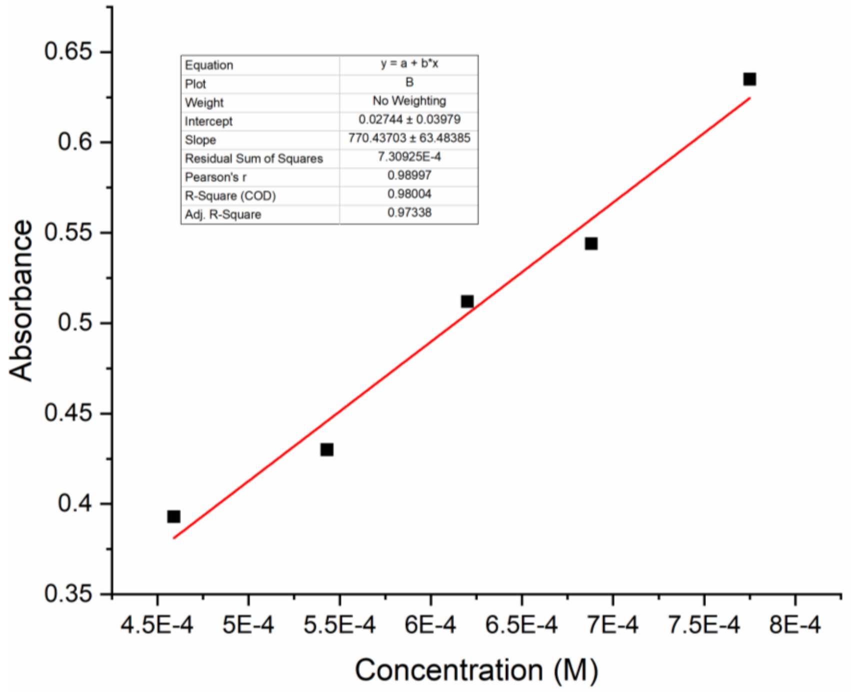

Figure S36: Linear regression of **4** in THF at 338 nm.

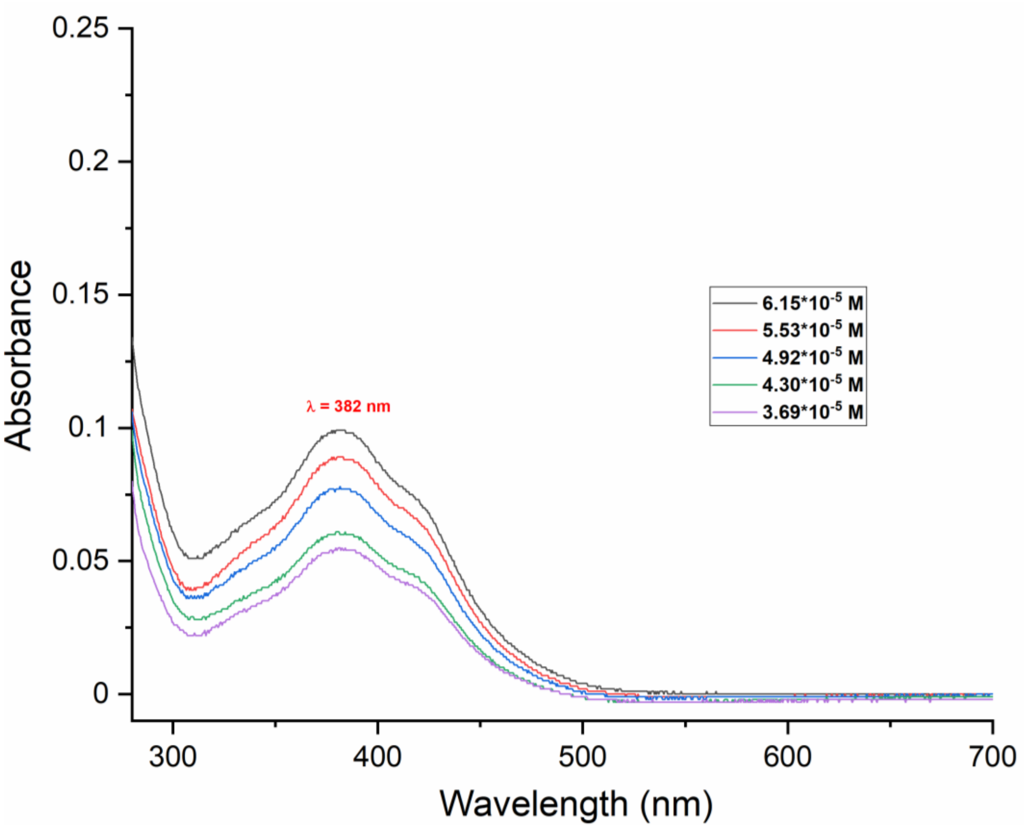

Figure S37: UV/vis spectra of compound **5** in Toluene at different concentrations.

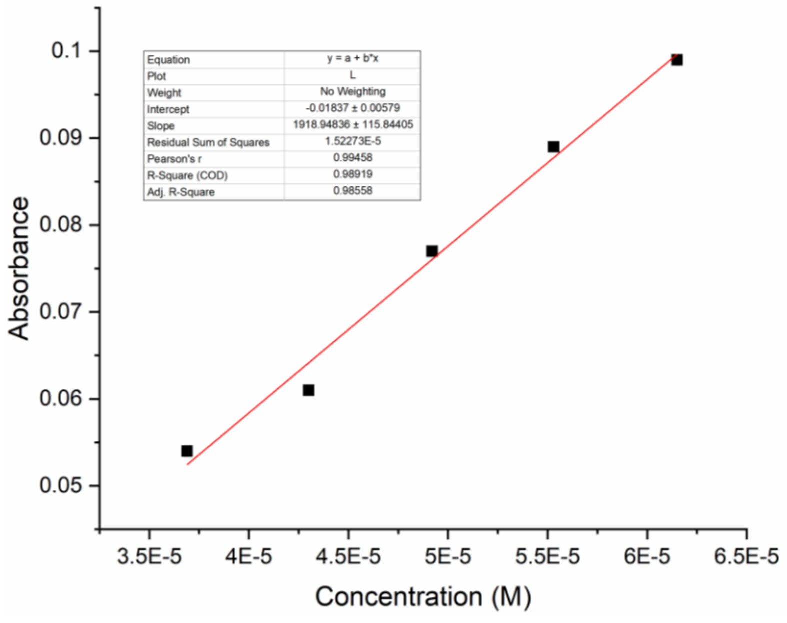

Figure S38: Linear regression of **5** in Toluene at 382 nm.

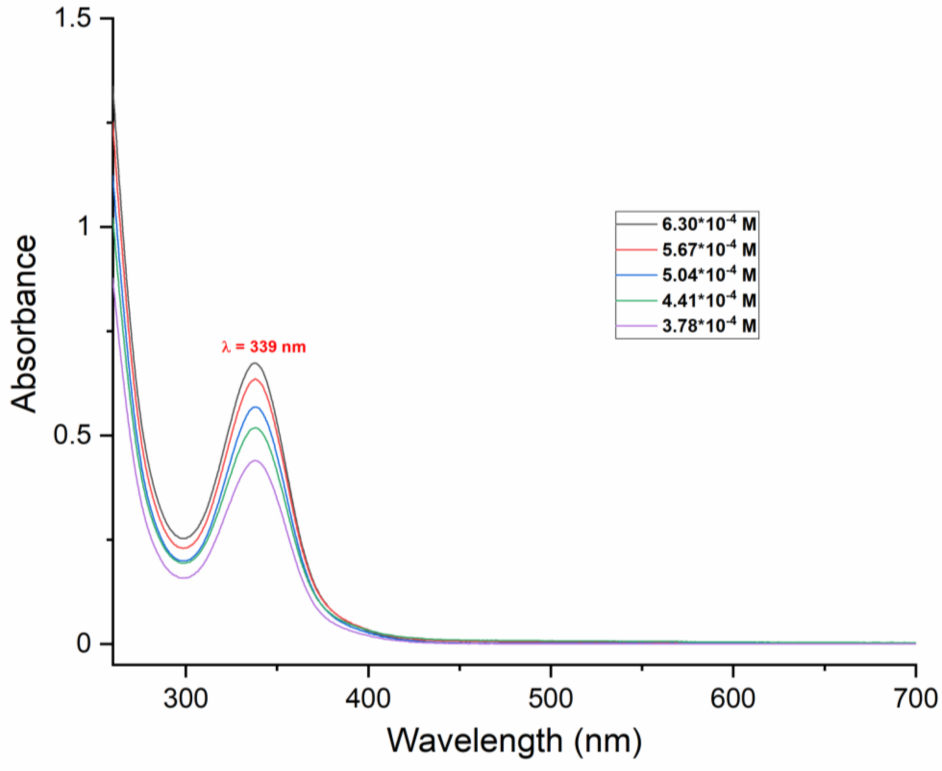

Figure S39: UV/vis spectra of compound **6** in THF at different concentrations.

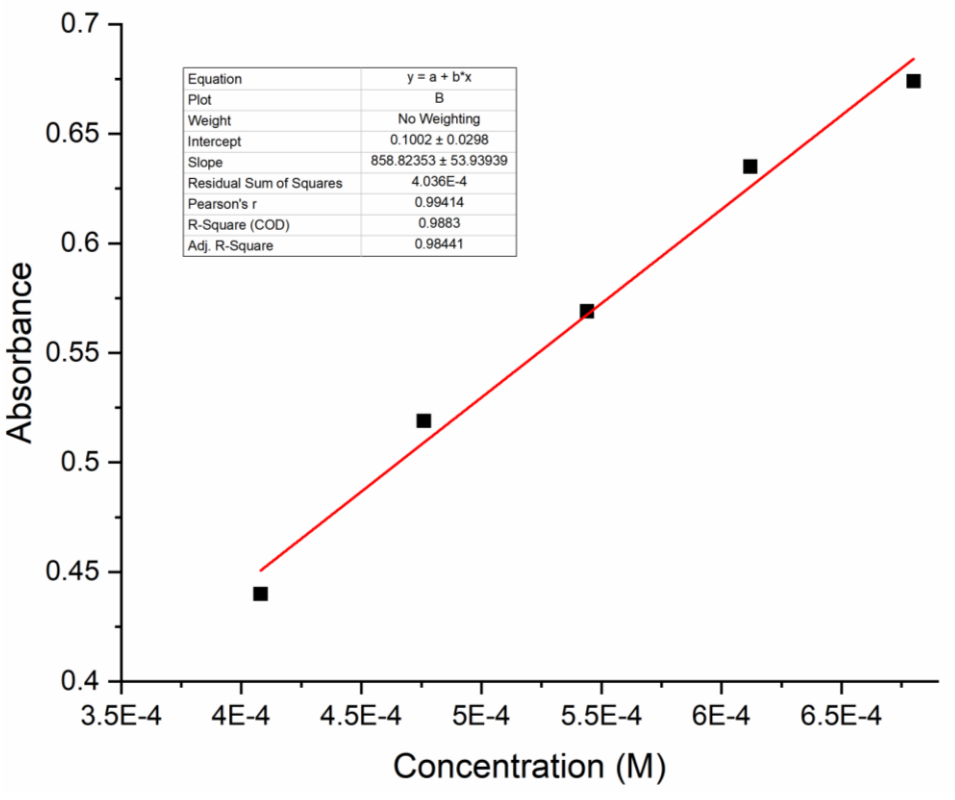

Figure S40: Linear regression of **6** in THF at 339 nm.

IR Spectra

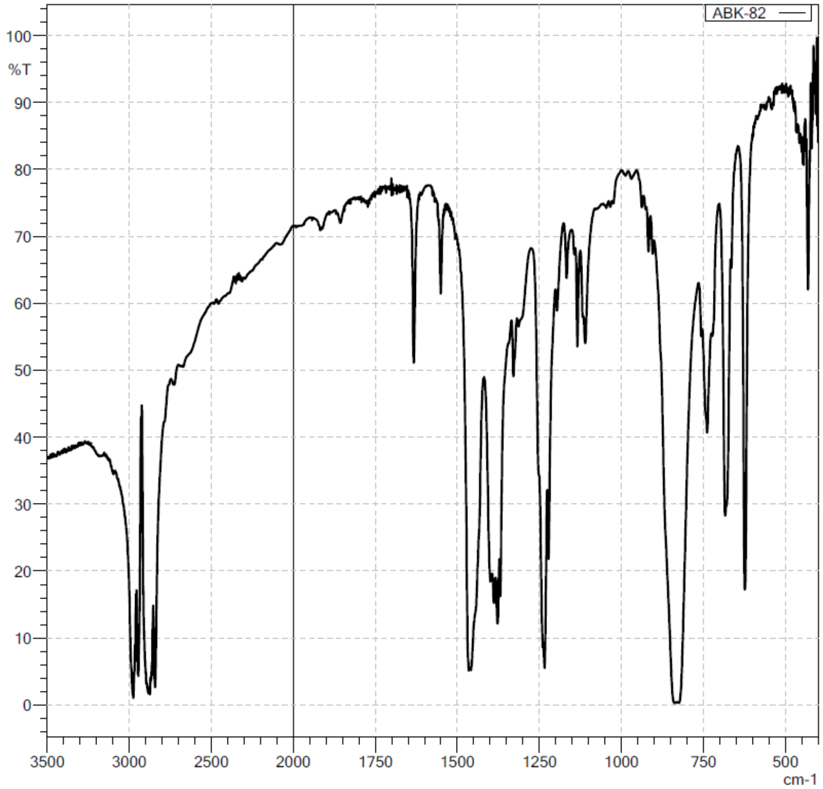

Figure S41: IR spectrum of **2**.

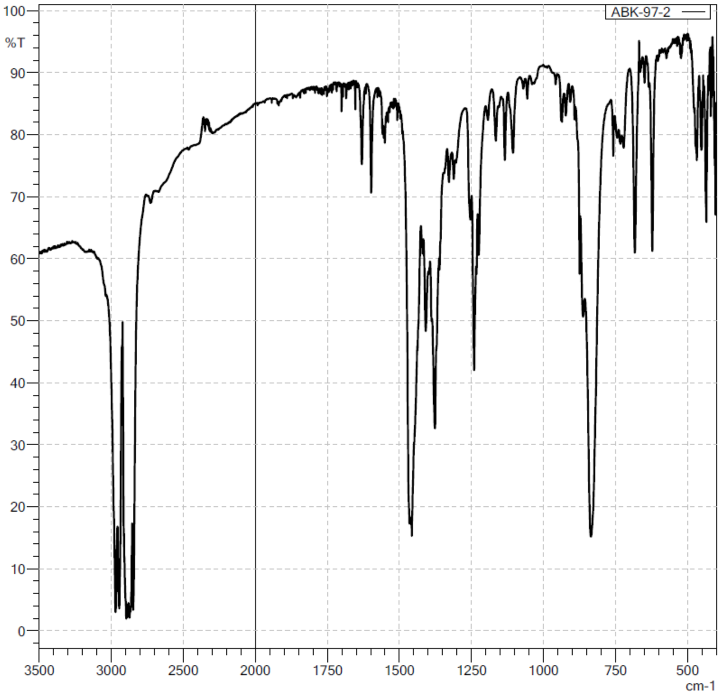

Figure S42: IR spectrum of **3**.

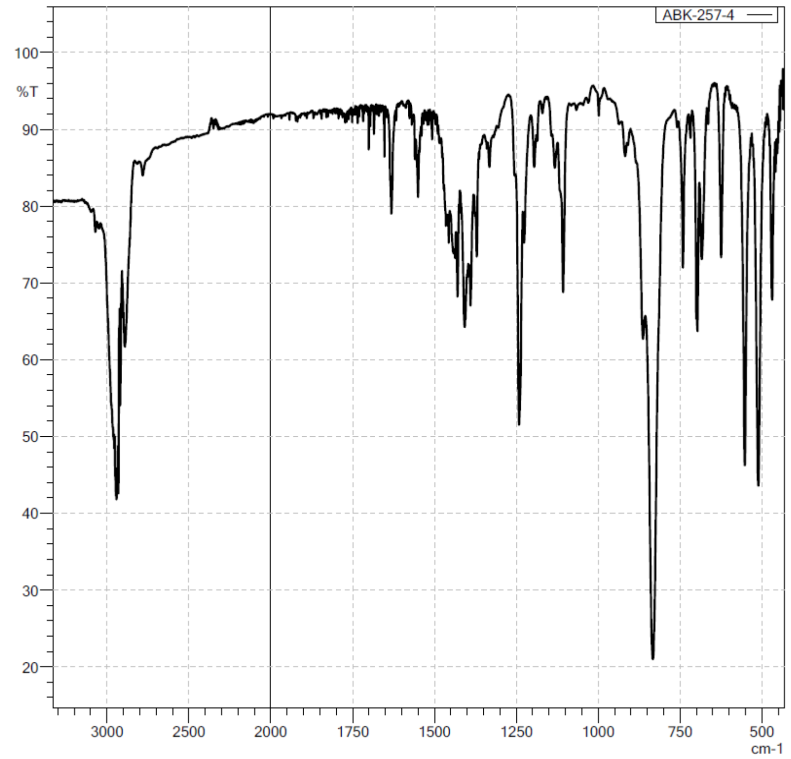

Figure S43: IR spectrum of **4**.

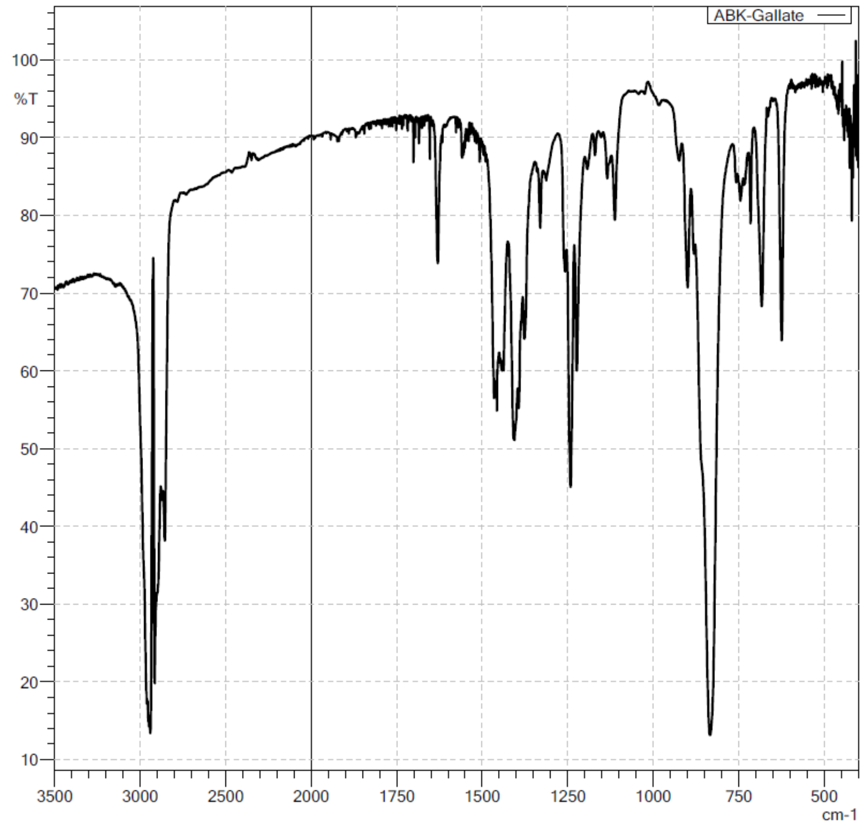

Figure S44: IR spectrum of **5**.

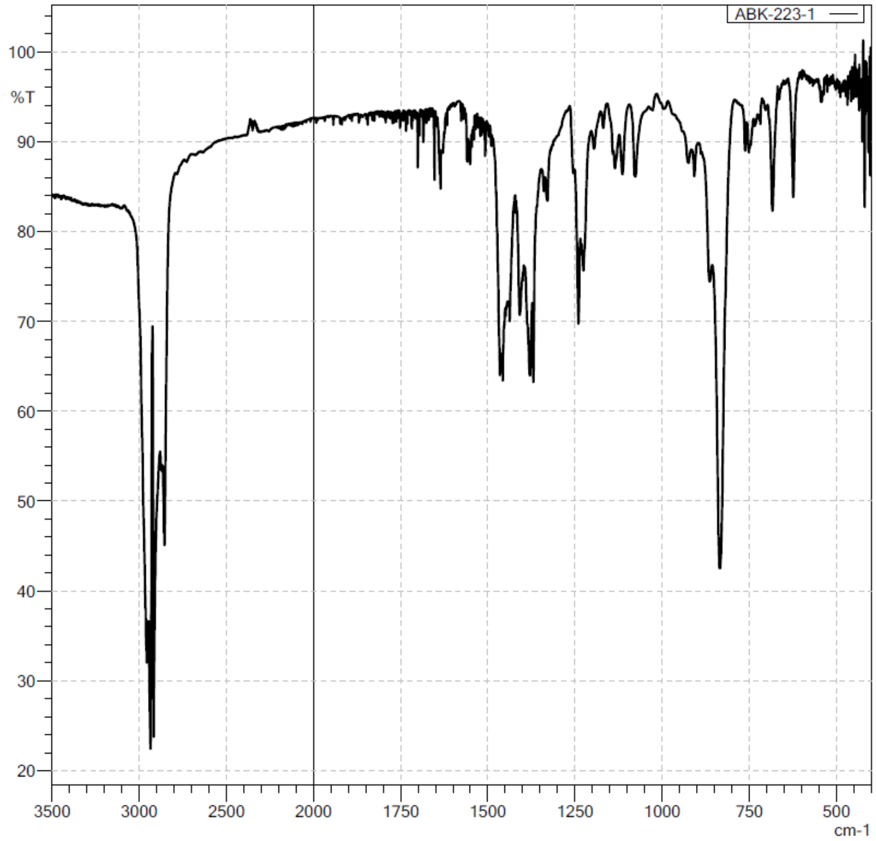

Figure S45: IR spectrum of **6**.

Crystallographic Details

All non H-atoms were located on the electron density maps and refined anisotropically. C-bound H atoms were placed in positions of optimized geometry and treated as riding atoms. Their isotropic displacement parameters were coupled to the corresponding carrier atoms by a factor of 1.2 (CH, CH2) or 1.5 (CH3). *Twinning and Disorder*: The compound **2** was refined as a two-component twin (twinmatrix: -1.000 0.000 0.000, 0.000 -1.000 0.000, -0.107 0.018 1.000) using the hklf5 routine, resulting in a BASF value of 0.209(2). Disorder over two positions was observed for solvent molecules in **4** (benzene), **5** (benzene) and **6** (diethylether) and for the $PSiCl_2Ph$ unit of compound **4**.

Compound 2

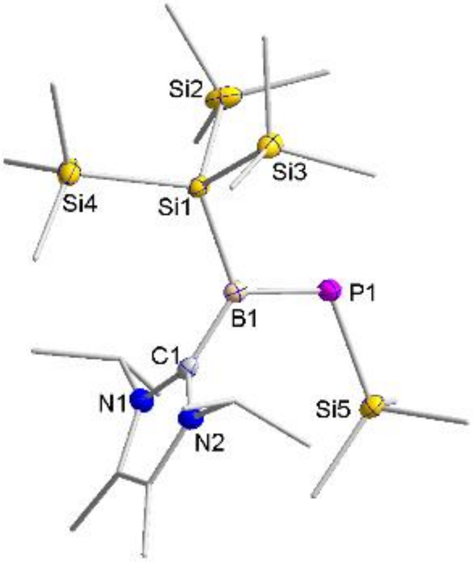

Sum Formula: $C_{23}H_{56}BN_2PSi_5$, Molecular weight = 542.92, space group P -1, Z = 2, a = 9.7123(5) Å, b = 12.0591(6) Å, c = 15.9058(8) Å, α = 89.691(2) °, β = 88.273(2) °, γ = 68.230(2) °, V = 1729.23(15) $Å^3$, d_c = 1.043 g cm^{-3}, μ = 0.267 mm^{-1}, T = 133 K, transmission factors (min./max.) 0.704/0.746, θ_{max}= 26.999°, no. of unique data 7547, R(reflections) = 0.0518(6420), wR2 (reflections) = 0.1505(7547), data completeness= 0.999. CCDC reference number: **2123818**.

Compound 3

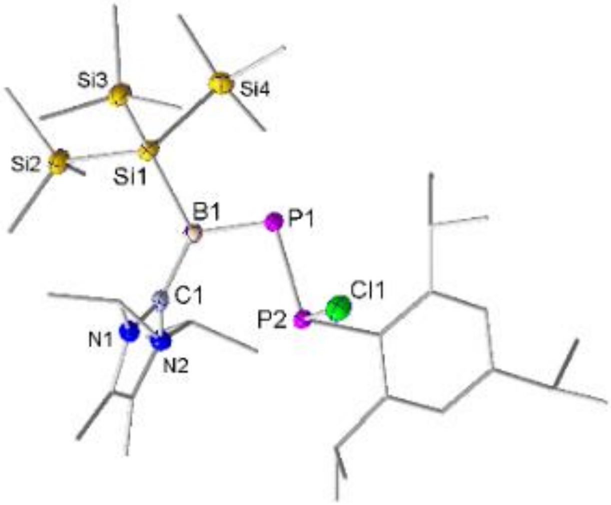

Sum Formula: $C_{35}H_{70}BClN_2P_2Si_4$, Molecular weight = 739.49, space group C 2/c, Z = 8, a = 49.249(4) Å, b = 10.0362(7) Å, c = 18.7530(12) Å, α = 90°, β = 96.529(4) °, γ = 90°, V = 9208.9(11) $Å^3$, d_c = 1.067 g cm^{-3}, μ = 0.281 mm^{-1}, T = 133 K, transmission factors (min./max.) 0.699/0.746, θ_{max}= 27.103°, no. of unique data 10147, R(reflections) = 0.0339(8305), wR2 (reflections) = 0.0827(10147), data completeness= 1.000, CCDC reference number: **2123819**.

Compound 4:

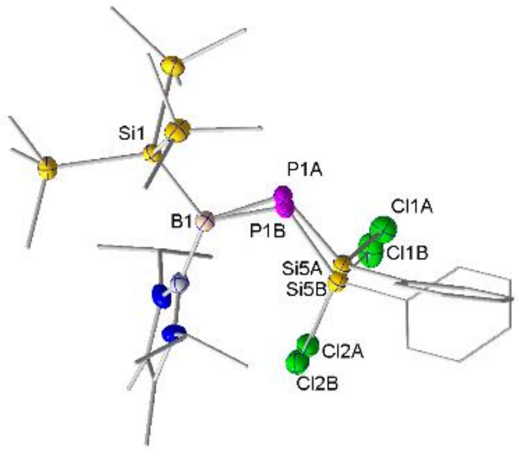

Sum Formula: $C_{32}H_{58}BCl_2N_2PSi_5$, Molecular weight = 723.93, space group P -1, Z = 2, a = 9.5866(6) Å, b = 9.9068(6) Å, c = 22.9521(15) Å, α = 93.713(2) °, β = 96.172(2) °, γ = 103.382(2) °, V = 2099.3(2) Å^3, d_c = 1.145 g cm^{-3}, μ = 0.359 mm^{-1}, T = 146 K, transmission factors (min./max.) 0.715/0.746, θ_{max}= 26.732°, no. of unique data 8923, R(reflections) = 0.0359(7236), wR2 (reflections) = 0.0912(8923), data completeness= 1.000. CCDC reference number: **2123825**.

Compound 5:

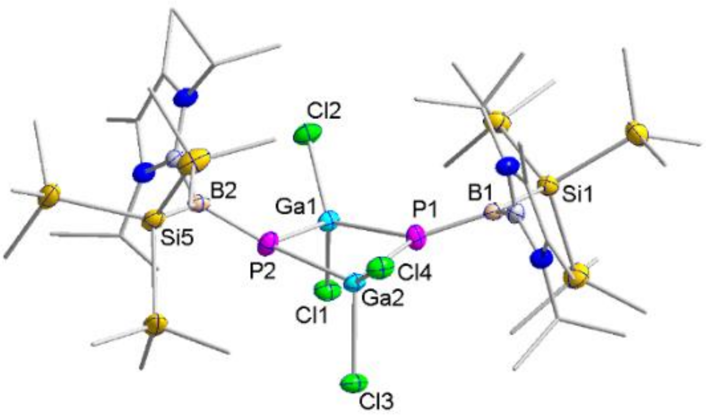

Sum Formula: $C_{58}H_{112}B_2Cl_4Ga_2N_4P_2Si_8$, Molecular weight = 1455.03, space group C 2/c, Z = 8, a = 47.0511(19) Å, b = 9.8045(4) Å, c = 41.3269(17) Å, α = 90 °, β = 121.862(1) °, γ = 90°, V = 16192.0(12) $Å^3$, d_c = 1.194 g cm^{-3}, μ = 0.990 mm^{-1}, T = 133 K, transmission factors (min./max.) 0.667/0.746, θ_{max}= 27.000°, no. of unique data 17711, R(reflections) = 0.0474(12712), wR2 (reflections) = 0.1044(17711), data completeness= 1.000. CCDC reference number: **2123822**.

Compound 6:

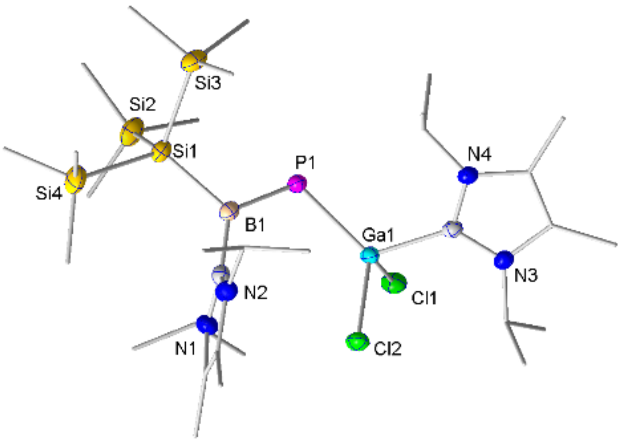

Sum Formula: $C_{35}H_{77}BCl_2GaN_4OPSi_4$, Molecular weight = 864.76, space group P21/c, Z = 4, a = 15.6571(3) Å, b = 10.1693(2) Å, c = 31.9859(7) Å, α = 90 °, β = 90.564(1) °, γ = 90°, V = 5092.60(18) $Å^3$, d_c = 1.128 g cm^{-3}, μ = 0.799 mm^{-1}, T = 133 K, transmission factors (min./max.) 0.726/0.746, θ_{max}= 27.117°, no. of unique data 11210, R(reflections) = 0.0269(10007), wR2 (reflections) = 0.0676(11210), data completeness= 0.999, CCDC reference number: **2123823.**

Computational Details

Geometry optimizations were performed using the Gaussian 16.C01 software.[7] All geometry optimizations were computed using the functional BP86[8] functional with Grimme dispersion corrections D3[9] and the Becke-Jonson damping function[10] in combination with the def2-SVP basis set.[11] The stationary points were located with the Berny algorithm[12] using redundant internal coordinates. Analytical Hessians were computed to determine the nature of stationary points (one and zero imaginary frequencies for transition states and minima, respectively)[13] and to calculate unscaled zero-point energies (ZPEs) as well as thermal corrections and entropy effects using the standard statistical-mechanics relationships for an ideal gas.

The atomic partial charges were estimated with the natural bond orbital (NBO)[14] method using NBO 7.0.[15] The topological quantum theory of atoms in molecules (QTAIM),[16] and Laplacian of the electron density analyses were carried out with AIMAII.[17] The calculations were performed at the BP86-D3(BJ)/def2-TZVPP level of theory.

TD-DFT calculations were performed using the ORCA 4.2.1 software.[18] Single point PCM/TD-B3LYP/def2-TZVPP[8a, 19] calculations were performed to estimate the change in the UV/Vis spectrum of the compounds in the presence of toluene solvent.

The nature of the chemical bonds were investigated by means of the Energy Decomposition Analysis (EDA) method, which was developed by Morokuma[20] and by Ziegler and Rauk.[21] The bonding analysis focuses on the instantaneous interaction energy ΔE_{int} of a bond A–B between two fragments A and B in the particular electronic reference state and in the frozen geometry AB. This energy is divided into four main components (Eq S1).

$$\Delta E_{\mathrm{int}} = \Delta E_{elst} + \Delta E_{Pauli} + \Delta E_{orb} + \Delta E_{disp} \quad \text{(S1)}$$

The term ΔE_{elst} corresponds to the classical electrostatic interaction between the unperturbed charge distributions of the prepared atoms (or fragments) and it is usually attractive. The Pauli repulsion ΔE_{Pauli} is the energy change associated with the transformation from the superposition of the unperturbed wave functions (Slater determinant of the Kohn-Sham orbitals) of the isolated fragments to the wave function Ψ0 = NÂ[ΨAΨB], which properly obeys the Pauli principle through explicit antisymmetrization (Â operator) and renormalization (N = constant) of the product wave function. It comprises the destabilizing interactions between electrons of the same spin on either fragment. The orbital interaction ΔE_{orb} accounts for charge transfer and polarization effects.[22] In the case that the Grimme dispersion corrections[9-10] are computed the term ΔE_{disp} is added to equation 1. Further details on the EDA method can be found in the literature.[23] In the case of the dimers, relaxation of the fragments to their equilibrium geometries at the electronic ground state is termed ΔE_{prep}, because it may be considered as preparation energy for

chemical bonding. The addition of ΔE_{prep} to the intrinsic interaction energy ΔE_{int} gives the total energy ΔE, which is, by definition, the opposite sign of the bond dissociation energy D_e:

$$\Delta E(-D_e) = \Delta E_{\text{int}} + \Delta E_{prep} \qquad \text{(S2)}$$

The EDA–NOCV method combines the EDA with the natural orbitals for chemical valence (NOCV) to decompose the orbital interaction term ΔE_{orb} into pairwise contributions. The NOCVs Ψi are defined as the eigenvector of the valence operator, $\widehat{V}$, given by Equation (S3).

$$\widehat{V}\Psi_i = v_i \Psi_i \qquad \text{(S3)}$$

In the EDA–NOCV scheme the orbital interaction term, ΔE_{orb}, is given by Equation (S4),

$$\Delta \mathrm{E}_{\mathrm{orb}} = \sum_{\mathrm{k}} \Delta \mathrm{E}_{\mathrm{k}} = \sum_{\mathrm{k}=1}^{\mathrm{N}/2} v_k \left[-\mathrm{F}_{\mathrm{-k,k}}^{\mathrm{TS}} + \mathrm{F}_{\mathrm{k,k}}^{\mathrm{TS}} \right] \qquad \text{(S4)}$$

in which $\mathrm{F}_{-\mathrm{k},-\mathrm{k}}^{\mathrm{TS}}$ and $\mathrm{F}_{\mathrm{k},\mathrm{k}}^{\mathrm{TS}}$ are diagonal transition state Kohn–Sham matrix elements corresponding to NOCVs with the eigenvalues –νk and νk, respectively. The ΔE_k^{orb} term for a particular type of bond is assigned by visual inspection of the shape of the deformation density Δρk. The latter term is a measure of the size of the charge deformation and it provides a visual notion of the charge flow that is associated with the pairwise orbital interaction. The EDA–NOCV scheme thus provides both qualitative and quantitative information about the strength of orbital interactions in chemical bonds. The EDA-NOCV calculations were carried out with ADF2019. The basis sets for all elements have triple-ζ quality augmented by two sets of polarizations functions and one set of diffuse function. Core electrons were treated by the frozen-core approximation. This level of theory is denoted BP86+D3(BJ)/TZ2P.[24] Scalar relativistic effects have been incorporated by applying the zeroth-order regular approximation (ZORA).[25]

Optimized Structures

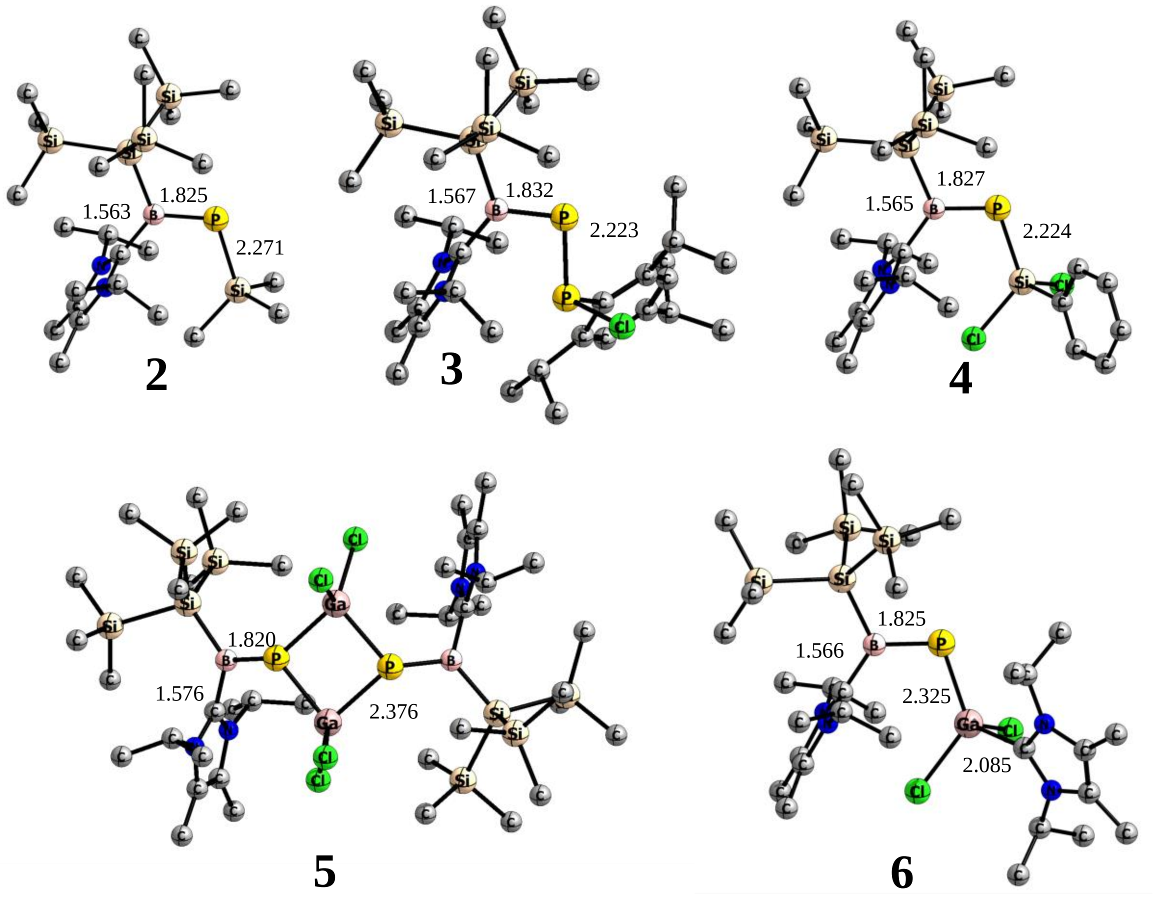

Figure S46. Optimized structures (experimental values in parenthesis) of compounds **2-6** at the BP86-D3(BJ)/def2-SVP level of theory. The bond lengths are in [Å]. The hydrogen atoms are omitted for clarity.

Frontier Molecular Orbitals

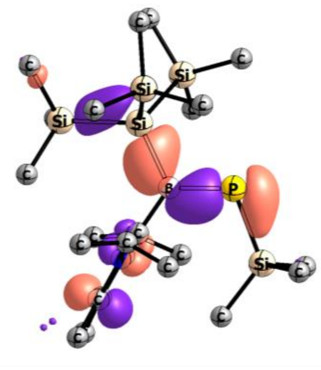

HOMO-2 (ε= -5.20 eV)

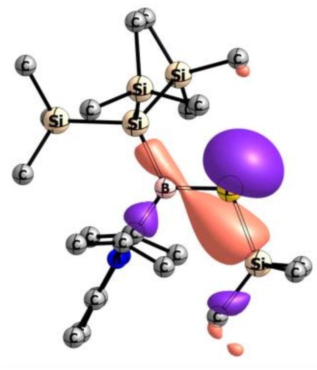

HOMO-1 (ε= -4.62 eV)

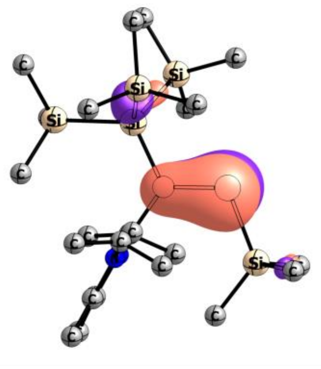

HOMO (ε= -4.47 eV)

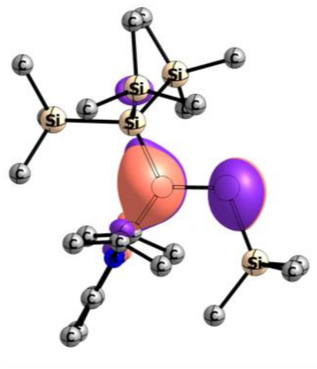

LUMO (ε= -1.65 eV)

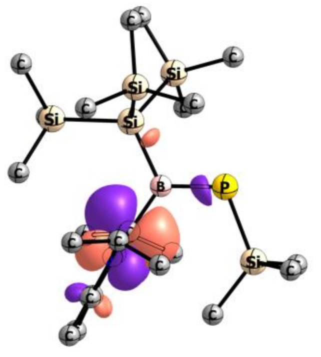

LUMO+1 (ε= -1.24 eV)

Figure S47. Frontier KS molecular orbitals (isovalue 0.05 a.u.) of compound **2** at the BP86-D3(BJ)/def2-TZVPP level of theory.

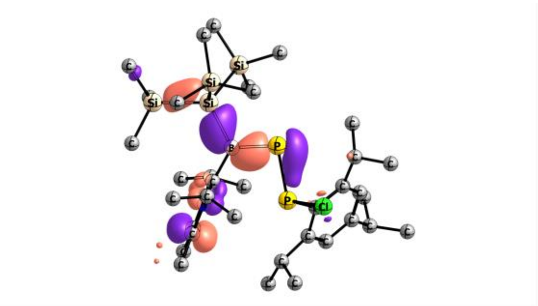

HOMO-2 (ε= -5.28 eV)

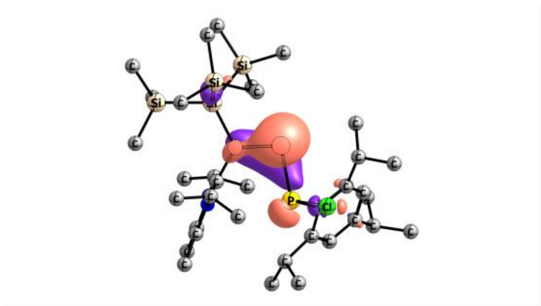

HOMO-1 (ε= -4.82 eV)

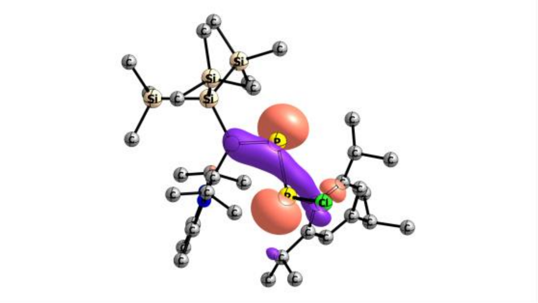

HOMO (ε= -4.56 eV)

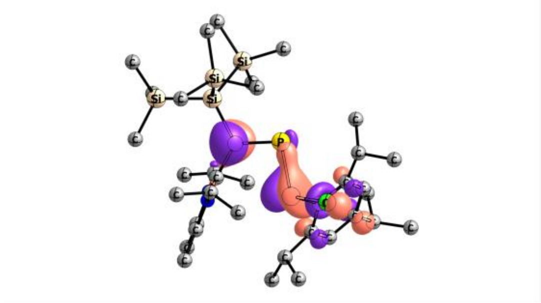

LUMO (ε= -2.13 eV)

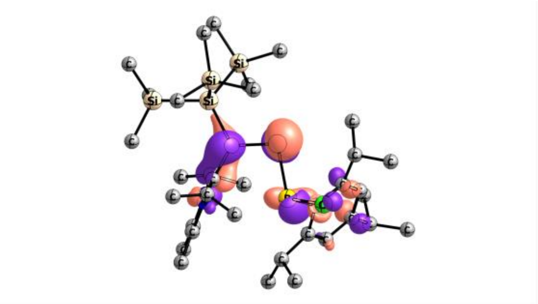

LUMO+1 (ε= -1.63 eV)

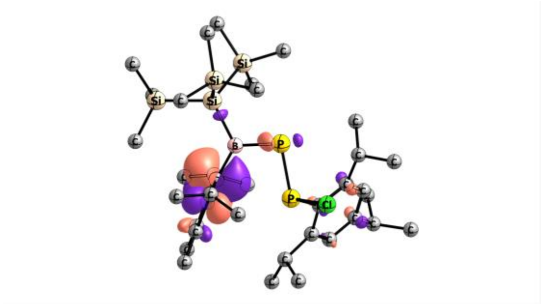

LUMO+2 (ε= -1.31 eV)

Figure S48. Frontier KS molecular orbitals (isovalue 0.05 a.u.) of compound **3** at the BP86-D3(BJ)/def2-TZVPP level of theory.

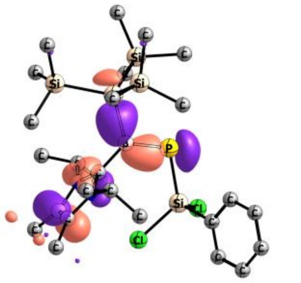

HOMO-3 (ε= -5.42 eV)

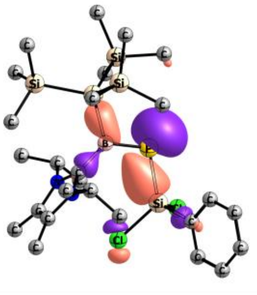

HOMO-2 (ε= -5.31 eV)

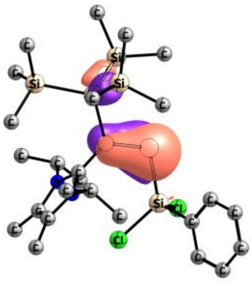

HOMO (ε= -4.91 eV)

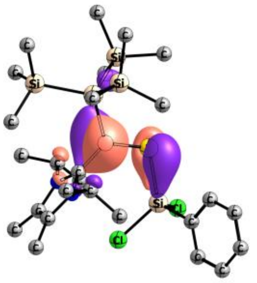

LUMO (ε= -2.06 eV)

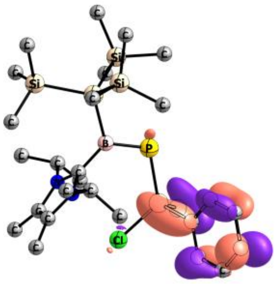

LUMO+1 (ε= -1.70 eV)

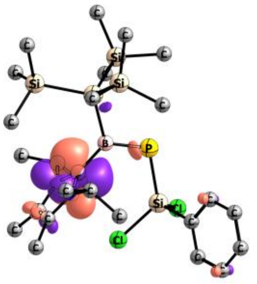

LUMO+2 (ε= -1.34 eV)

Figure S49. Frontier KS molecular orbitals (isovalue 0.05 a.u.) of compound **4** at the BP86-D3(BJ)/def2-TZVPP level of theory.

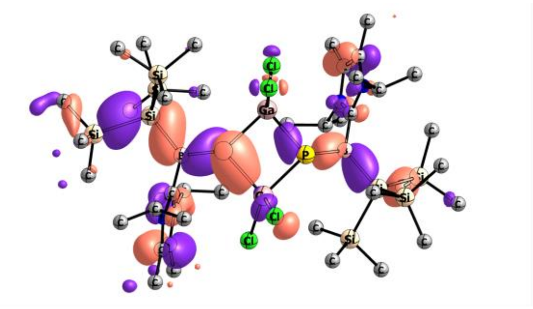

HOMO-3 (ε= -5.37 eV)

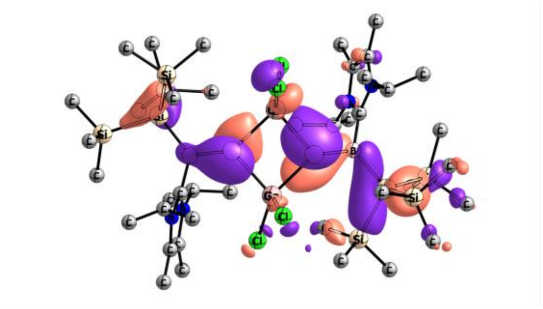

HOMO-2 (ε= -5.30 eV)

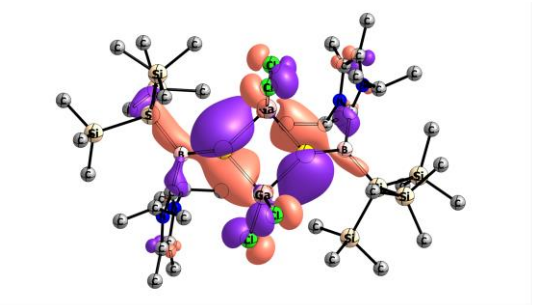

HOMO-1 (ε= -5.05 eV)

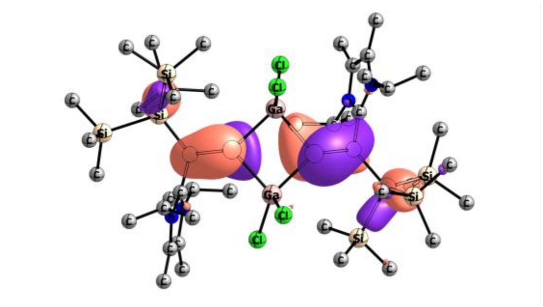

HOMO (ε= -4.88 eV)

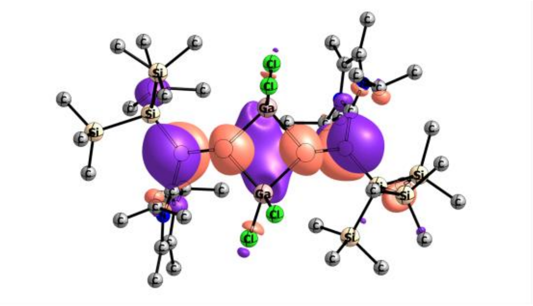

LUMO (ε= -2.63 eV)

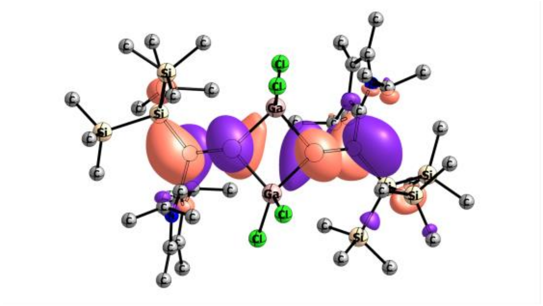

LUMO+1 (ε= -1.93 eV)

Figure S50. Frontier KS molecular orbitals (isovalue 0.05 a.u.) of compound **5** at the BP86-D3(BJ)/def2-TZVPP level of theory.

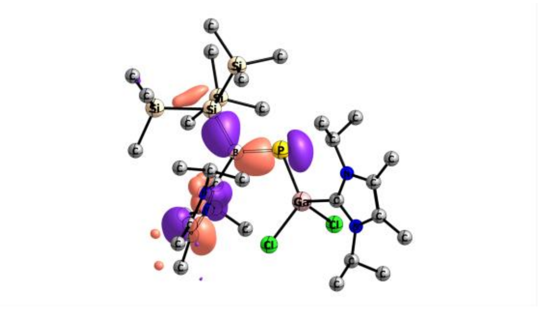
HOMO-2 (ε= -4.97 eV)

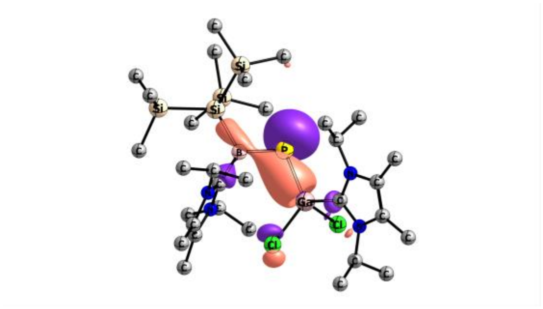
HOMO-1 (ε= -4.54 eV)

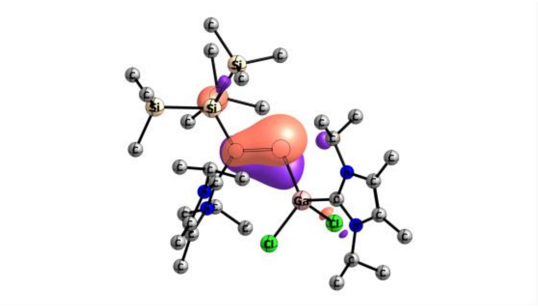
HOMO (ε= -4.39 eV)

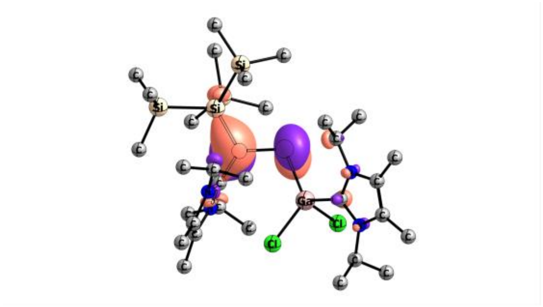
LUMO (ε= -1.59 eV)

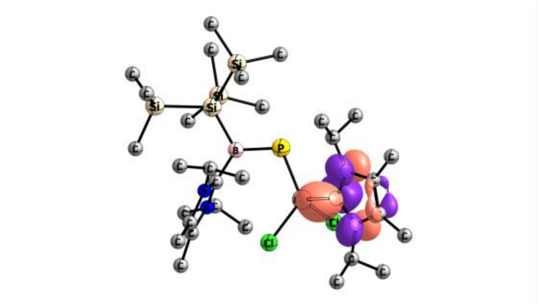
LUMO+1 (ε= -1.29 eV)

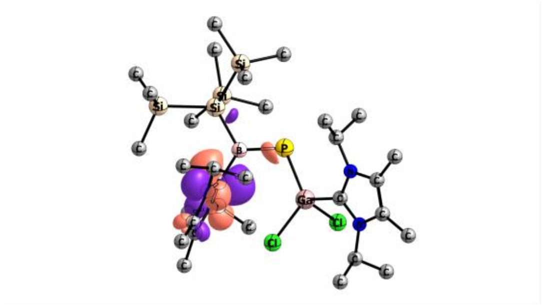
LUMO+2 (ε= -0.93 eV)

Figure S51. Frontier KS molecular orbitals (isovalue 0.05 a.u.) of compound **6** at the BP86-D3(BJ)/def2-TZVPP level of theory.

NBO Results

Table S1. Natural Partial Charges (q in a.u.) and Wiberg bond orders (P in a.u.) of compound **2**, **3**, **4**, **5**, and **6** at the BP86-D3(BJ)/def2-TZVPP//BP86-D3(BJ)/def2-SVP level of theory.

Property	**2**	**3**	**4**	**5**	**6**
Q(P)	-0.28	-0.11	-0.30	-0.44	-0.43
Q(B)	-0.36	-0.33	-0.28	-0.21	-0.32
Q(E)[a]	+0.09	-0.16	-0.03	-0.06	+0.18
Q(C_{carb})	+0.18	+0.18	+0.17	+0.17	+0.18
Q(NHC)	+0.44	+0.47	+0.48	+0.53	+0.48
P(B-P)	1.78	1.68	1.71	1.59	1.75
P(NHC-B)	0.88	0.88	0.87	0.88	0.88

[a] E stands for TMS (**2**), PClTip (**3**), $SiCl_2Ph$ (**4**), $GaCl_2$ (**5**), and $GaCl_2NHC$ (**6**).

Orbital	Occ.	Contribution from atoms to the orb	Atomic orbitals
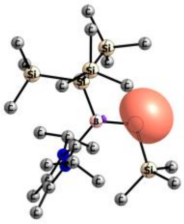	1.89	LP(P)	P: *s*(56.50%), *p*(43.38%), *d*(0.11%)
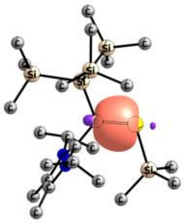	1.93	P (53.83%) - B (46.17%)	P: *s*(29.46%), *p*(69.88%), *d*(0.63%)<br>B: s(36.79%), *p*(63.00%), *d*(0.10%)
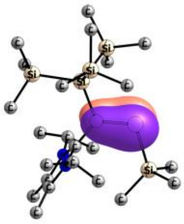	1.88	P (61.51%) - B (38.49%)	P: *s*(0.00%), *p*(99.56%), *d*(0.42%)<br>B: *s*(0.00%), *p*(99.90%),*d*(0.04%)
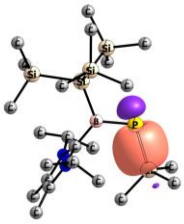	1.94	P (57.93%) - Si (42.07%)	P: *s*(14.06%), *p*(85.05%), *d*(0.85%)<br>Si: *s*(27.56%), *p*(72.03%), *d*(0.39%)
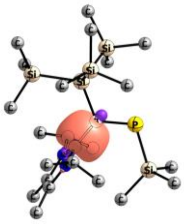	1.96	B (30.31%) - C (69.69%)	B: *s*(44.51%), *p*(55.42%), *d*(0.03%)<br>C: *s*(29.81%), *p*(70.09%), d(0.06%)

Figure S52. NBO results at the BP86-D3(BJ)/def2-TZVPP//BP86-D3(BJ)/def2-SVP of compound **2**.

Orbital	Occ.	Contribution from atoms to the orb	Atomic orbitals
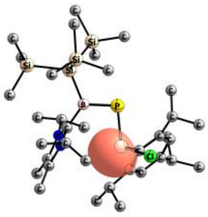	1.95	LP(P)	P: *s*(63.18%), *p*(36.76%), *d*(0.06%)
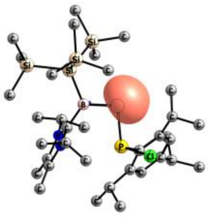	1.91	LP(P)	P: *s*(62.94%), *p*(36.94%), *d*(0.12%)
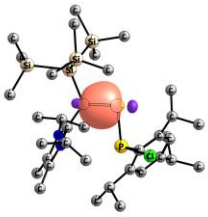	1.93	P (54.10%) - B (45.90%)	P: *s*(25.80%), *p*(73.40%), *d*(0.76%)<br>B: *s*(35.14%), *p*(64.65%), *d*(0.11%)
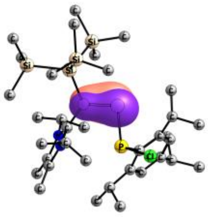	1.83	P (63.18%) - B (36.82%)	P: *s*(0.90%), *p*(98.57%), *d*(0.51%)<br>B: *s*(1.01%), *p*(98.86%), *d*(0.07%)
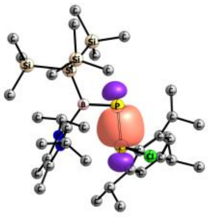	1.95	P (53.70%) - P (46.30%)	P: *s*(15.11%), *p*(84.29%), *d*(0.59%)<br>P: *s*(10.44%), *p*(88.61%), *d*(0.92%)
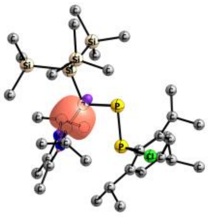	1.96	B (30.46%) - C (69.54%)	B: *s*(30.41%), *p*(69.50%), *d*(0.06%)<br>C: *s*(44.12%), *p*(55.80%), *d*(0.04%)

Figure S53. NBO results at the BP86-D3(BJ)/def2-TZVPP//BP86-D3(BJ)/def2-SVP of compound **3**.

Orbital	Occ.	Contribution from atoms to the orb	Atomic orbitals
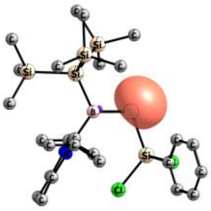	1.87	LP(P)	P: *s*(58.24%), *p*(41.62%), d (0.14%)
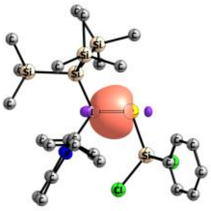	1.94	P (55.01%) - B (44.99%)	P: s(28.97%)p 2.43(70.29%)d 0.02(0.70%)<br>B: s(35.81%)p 1.79(63.98%)d 0.00(0.10%)
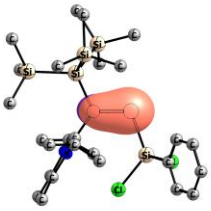	1.86	P (61.51%) - B (35.03%)	P: *s*(0.09%), *p*(99.44%), *d*(0.45%)<br>B: *s*(0.24%), *p*(99.66%), *d*(0.05%)
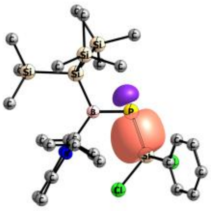	1.94	P (54.72%) - Si (45.28%)	P: *s*(12.67%), *p*(86.39%), *d*(0.91%)<br>Si: *s*(36.56%), *p*(62.87%), *d*(0.54%)
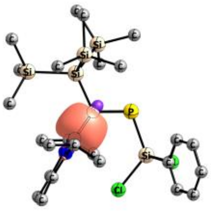	1.96	B (30.28%) - C (69.69%)	B: *s*(30.56%), *p*(69.34%), *d*(0.06%)<br>C: *s*(43.75%), *p*(56.17%), *d*(0.04%)

Figure S54. NBO results at the BP86-D3(BJ)/def2-TZVPP//BP86-D3(BJ)/def2-SVP of compound **4**.

Orbital	Occ.	Contribution from atoms to the orb	Atomic orbitals
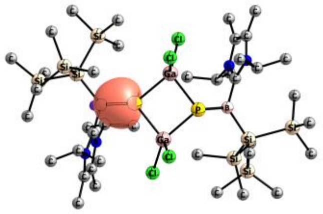	1.95	P (58.43%) - B (41.57%)	P: *s*(45.53%), *p*(54.08%), *d*(0.34%)<br>B: *s*(32.33%), *p*(67.51%), *d*(0.09%)
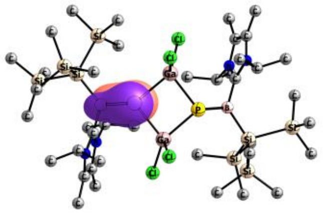	1.88	P (69.53%) - B (30.47%)	P: *s*(0.63%), *p*(99.25%), *d* (0.06%)<br>B: *s*(0.10%), *p*(99.76%), *d*(0.10%)
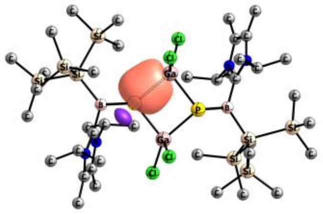	1.91	P (76.62%)- Ga (23.38%)	P: *s*(25.69%), *p*(74.02%), d(0.27%)<br>Ga: *s*(29.52%), *p*(70.26%), *d*(0.20%)
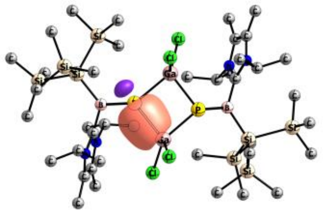	1.89	P (73.67%) - Ga (26.33%)	P: *s*(28.65%), *p*(71.11%), *d*(0.22%)<br>Ga: *s*(24.79%), *p*(74.89%),*d*(0.30%)
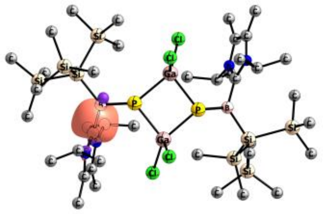	1.96	B (30.84%) - C (69.16%)	B: *s*(29.00%),*p*(70.91%), *d*(0.07%)<br>C: *s*(43.46%), *p*(56.47%), *d*(0.03%)

Figure S55. NBO results at the BP86-D3(BJ)/def2-TZVPP//BP86-D3(BJ)/def2-SVP of compound **5**.

Orbital	Occ.	Contribution from atoms to the orb	Atomic orbitals
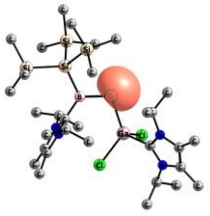	1.86	LP(P)	P: *s*(57.31%), *p*(42.57%), *d*(0.12%)
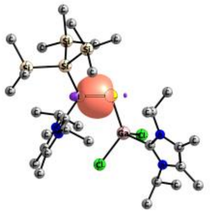	1.94	P (54.05%) - B (45.95%)	P: *s*(37.06%), *p*(62.71%), d(0.12%)<br>B: *s*(29.28%), *p*(70.06%), *d*(0.63%)
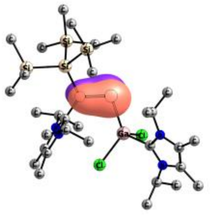	1.87	P (63.47%) - B (36.53%)	P: *s*(0.00%), *p*(99.59%), *d*(0.40%)<br>B: *s*(0.02%), *p*(99.87%), *d*(0.04%)
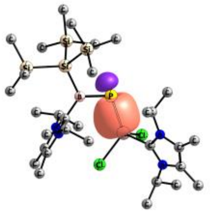	1.89	P (65.33%) - Ga (34.67%)	P: *s*(13.44%), *p*(85.89%), *d*(0.66%)<br>Ga: *s*(42.99%), *p*(56.79%), *d*(0.20%)
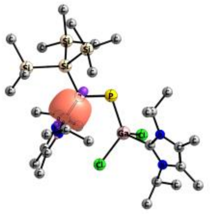	1.96	B (30.31%) - C (69.69%)	B: *s*(30.14%), *p*(69.76%), *d*(0.07%)<br>C: *s*(44.15%), *p*(55.77%), *d*(0.04%)

Figure S56. NBO results at the BP86-D3(BJ)/def2-TZVPP//BP86-D3(BJ)/def2-SVP of compound **6**.

AIM Analysis

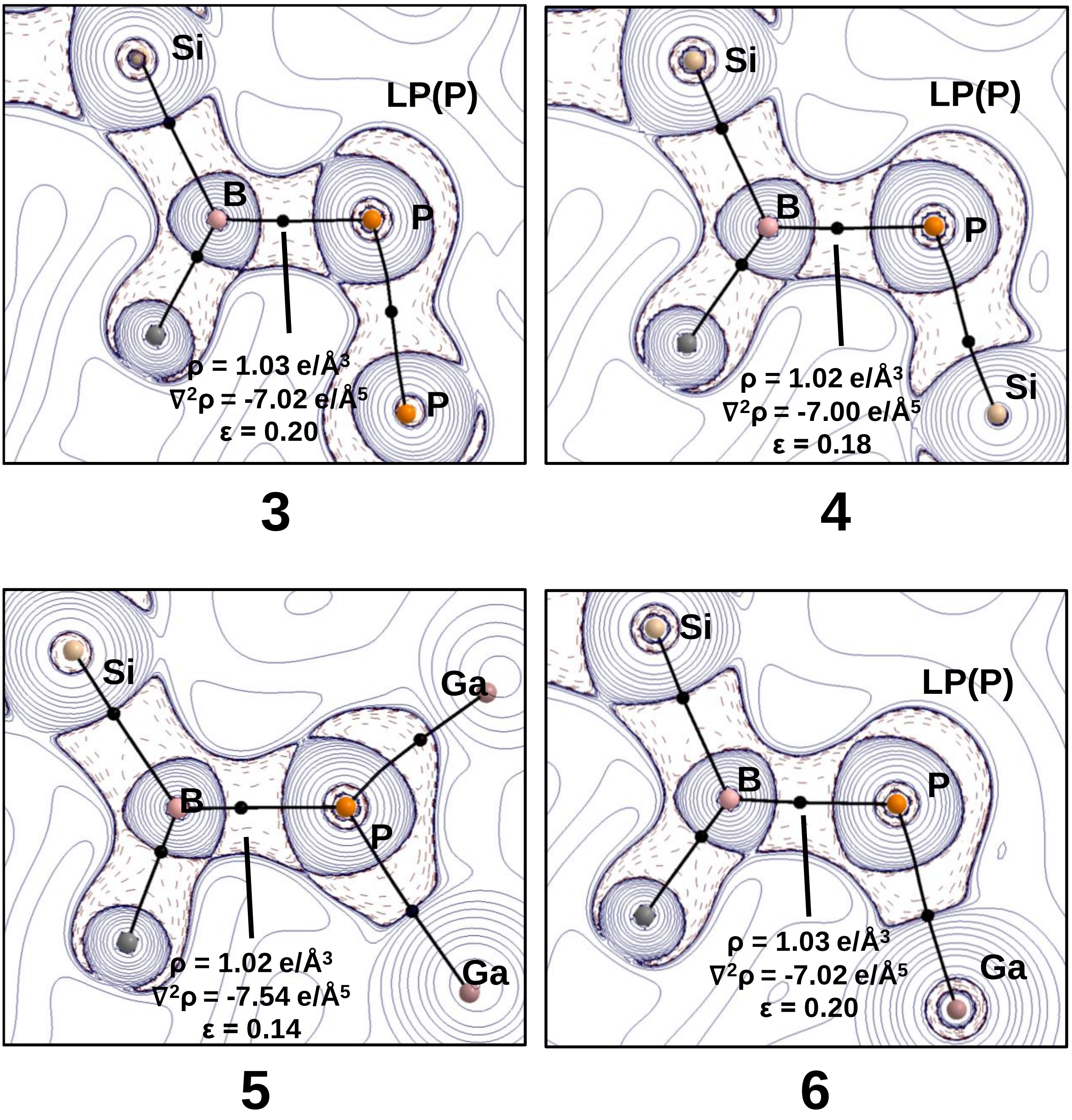

Figure S57. Contour line diagrams of the Laplacian distribution for compounds **3**, **4**, **5**, and **6**. Dashed red lines indicate areas of charge concentration ($\nabla^2\rho(r)<0$) while solid blue lines show areas of charge depletion ($\nabla^2\rho(r)>0$). The thick solid lines connecting the atomic nuclei are the bond paths and the small dots are the bond critical points and ring critical point.

Energy Decomposition Analysis

Table S2. EDA-NOCV results of the B−C_{carb} bond in complexes **VI**, **VII, 2** at BP86-D3(BJ)/TZ2P. All values are in kcal mol^{-1}.[a]

	**2**	**VI**	**VII**
ΔE_{int}	−97.5	−106.9	−86.4
ΔE_{Pauli}	317.7	288.3	328.6
ΔE_{disp}[b]	−32.3 (7.8 %)	−30.4 (7.3 %)	−27.9 (6.7 %)
ΔE_{elstat}[b]	−191.0 (46.0 %)	−184.8 (44.5 %)	−196.4 (47.3 %)
ΔE_{orb}[b]	−191.9 (46.2 %)	−180.1 (43.4 %)	−190.7 (45.9 %)
$\Delta E_{prep\ NHC}$	3.1	1.5	1.5
$\Delta E_{prep\ BP}$	29.4	40.7	33.3
$\Delta E_{prep\ tot}$	32.5	42.2	34.7
$-D_e$	−65.0	−64.7	−51.7

[a] Calculations were performed on the BP86-D3(BJ)/def2-SVP optimized structures. [b] The value in parenthesis gives the percentage contribution to the total attractive interactions $\Delta E_{\mathrm{elstat}} + \Delta E_{\mathrm{orb}} + \Delta E_{\mathrm{disp}}$.

TD-DFT calculation of UV-visible spectra

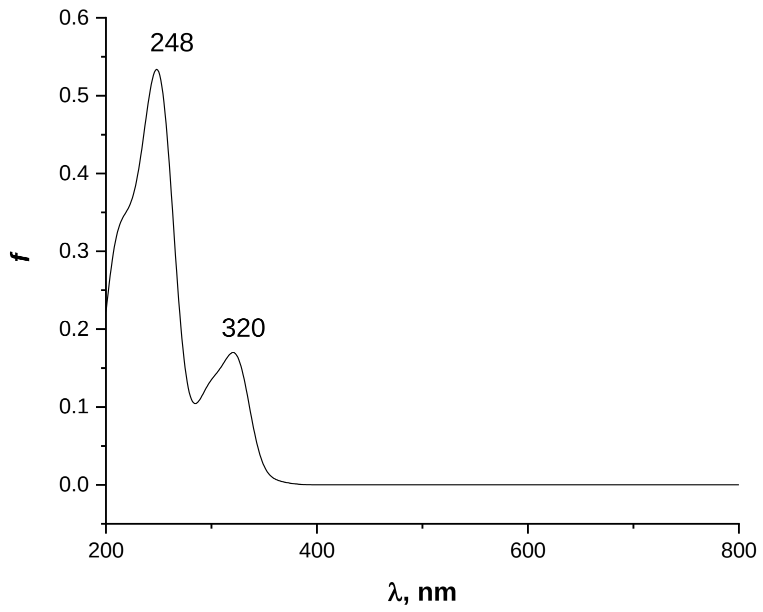

Figure S58. UV-visible spectrum of **2** calculated at the TD-PCM(toluene)/B3LYP/def2-TZVPP//BP86-D3(BJ)/def2-SVP level of theory. The middle bandwidth was arbitrary chosen as 30 nm.

Table S3. TD-PCM(toluene)/B3LYP/def2-TZVPP//BP86-D3(BJ)/def2-SVP results for compound **2**. Wavelength (λ), oscillator strength (f) and main assignment.[a]

	**λ(nm)**	**f**	**Assignment[b]**
1	324.0	0.150722108	H → L 0.697645 (c= 0.83525174)
2	299.1	0.089555027	H-1 → L+1 0.740415 (c= 0.86047362)
3	253.8	0.078354228	H→L+4 0.457588 (c= -0.67645265)
4	250.8	0.129682365	H-4 → L 0.233428 (c= 0.48314362)
5	251.2	0.098541164	H-3 → L+1 0.822011 (c= 0.90664824)
6	263.5	0.041469095	H-1 → L+3 0.882063 (c= 0.93918235)
7	250.6	0.044311400	H → L+6 0.541910 (c= -0.73614542)
8	238.6	0.062908831	H-1 → L+4 0.293152 (c= -0.54143540)
9	230.9	0.050131676	H-1 → L+7 0.614667 (c= -0.78400720)
	208.3	0.068838898	H-2 → L+6 0.422207 (c= -0.64977455)

[a]H means HOMO and L means LUMO. [b]corresponds to the coefficient of the excitation to the CI wave function.

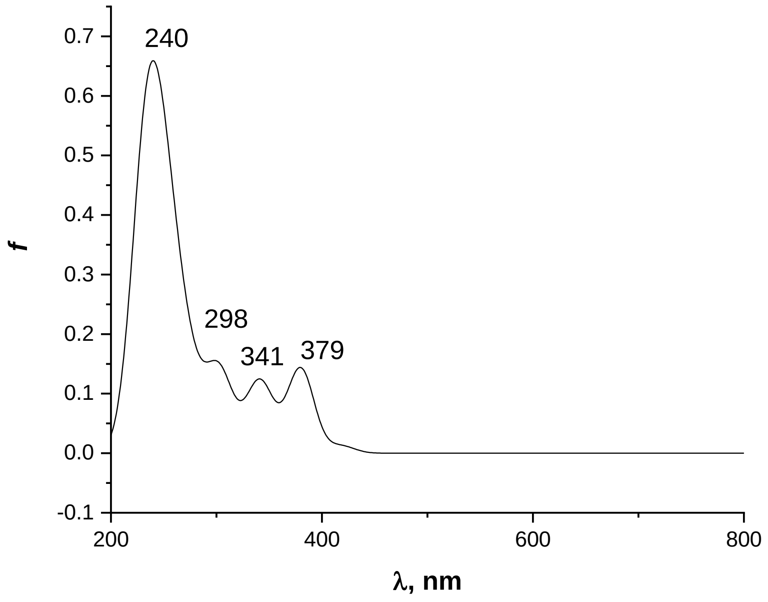

Figure S59. UV-visible spectrum of **3** calculated at the TD-PCM(toluene)/B3LYP/def2-TZVPP//BP86-D3(BJ)/def2-SVP level of theory. The middle bandwidth was arbitrary chosen as 30 nm.

Table S4. TD-PCM(toluene)/B3LYP/def2-TZVPP//BP86-D3(BJ)/def2-SVP results for compound **3**. Wavelength (λ), oscillator strength (f) and main assignment.[a]

	**λ(nm)**	**f**	**Assignment**[b]
1	379.7	0.142538227	H-1→L 0.605054 (c= -0.77785216)<br>H→L 0.205369 (c= 0.45317628)
2	343.0	0.080152482	H→L+1 0.360154 (c= -0.60012792)
3	337.4	0.042504111	H-2→L 0.576482 (c= 0.75926403)
4	302.3	0.062195385	H-1→L+1 0.325801 (c= 0.57078962)
5	268.9	0.042647027	H-6→L 0.621912 (c= -0.78861426)
6	264.3	0.031373489	H-1→L+4 0.725263 (c= -0.85162361)
7	254.4	0.117453234	H-2 →L+2 0.513854 (c= -0.71683620)
8	247.8	0.066808122	H-5→L+1 0.296168 (c= 0.54421300)
9	241.4	0.059844407	H-1 →L+6 0.301872 (c= -0.54942883)
10	238.6	0.044391529	H→L+8 0.300980 (c= 0.54861681)
11	234.0	0.054549899	H-7 → L+1 0.225765 (c= 0.47514712)<br>H-2 →L+4 0.220261 (c= 0.46931933)
12	233.1	0.062311696	H-1→L+7 0.599423 (c= -0.77422419)
13	234.0	0.085391632	H-1→L+8 0.463635 (c= 0.68090715)

[a]H means HOMO and L means LUMO. [b] corresponds to the coefficient of the excitation to the CI wave function.

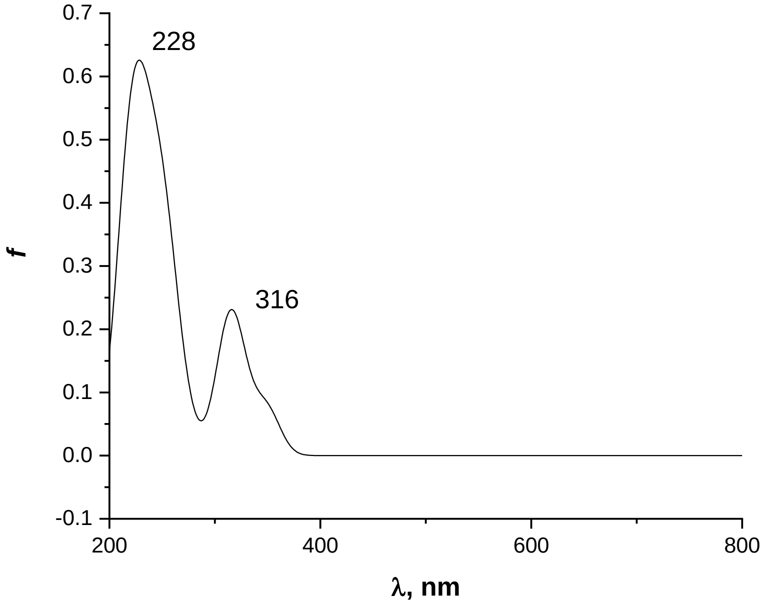

Figure S60. UV-visible spectrum of **4** calculated at the TD-PCM(toluene)/B3LYP/def2-TZVPP//BP86-D3(BJ)/def2-SVP level of theory. The middle bandwidth was arbitrary chosen as 30 nm.

Table S5. TD-PCM(toluene)/B3LYP/def2-TZVPP//BP86-D3(BJ)/def2-SVP results for compound **4**. Wavelength (λ), oscillator strength (f) and main assignment.[a]

	**λ(nm)**	**f**	**Assignment[b]**
1	349.3	0.070424825	H→L 0.296298 (c= 0.54433264)<br>H→L+1 0.495847 (c= 0.70416410)
2	315.3	0.153041489	H-2→L 0.250658 (c= -0.50065771)<br>H→L 0.404145 (c= 0.63572381)<br>H→L+1 0.210311 (c= -0.45859664)
3	264.9	0.040512174	H-2→L+3 0.430257 (c= -0.65594012)
4	249.4	0.147048290	H-1→L+2 0.234289 (c= -0.48403414)<br>H-1→L+3 0.214834 (c= 0.46350208)
5	250.6	0.040157515	H-3→L+2 0.382945 (c= 0.61882583)<br>H-3→L+3 0.508430 (c= -0.71304261)
6	248.2	0.064706204	H→L+5 0.396334 (c= 0.62955049)
7	240.5	0.051734872	H-4→L 0.262570 (c= 0.51241542)<br>H-4→L+1 0.170338 (c= -0.41271995)
8	231.6	0.081967541	H-2→L+4 0.356394 (c= -0.59698784)
9	228.3	0.169561418	H-2→L+4 0.321065 (c= 0.56662616)<br>H→L+8 0.483597 (c= 0.69541117)
10	220.3	0.074270871	H-5→L+1 0.222142 (c= 0.47131981)

[a]H means HOMO and L means LUMO. [b] corresponds to the coefficient of the excitation to the CI wave function.

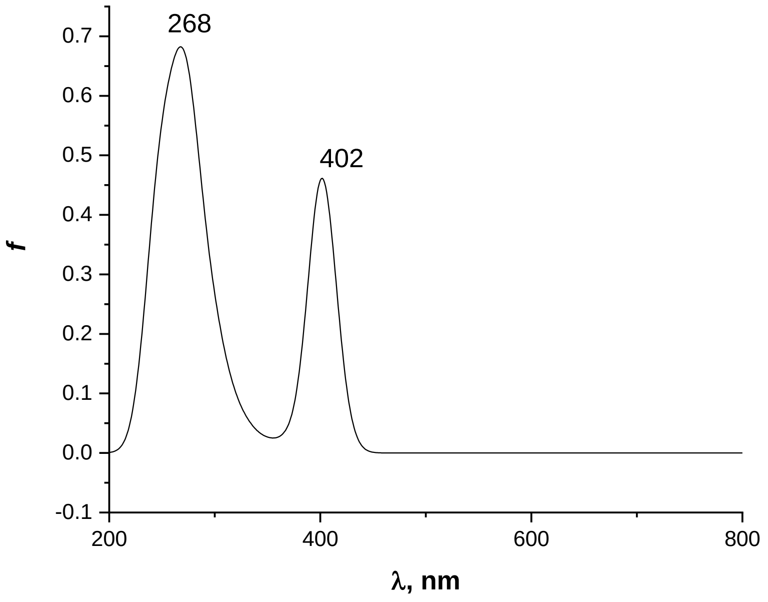

Figure S61. UV-visible spectrum of **5** calculated at the TD-PCM(toluene)/B3LYP/def2-TZVPP//BP86-D3(BJ)/def2-SVP level of theory. The middle bandwidth was arbitrary chosen as 30 nm.

Table S6. TD-PCM(toluene)/B3LYP/def2-TZVPP//BP86-D3(BJ)/def2-SVP results for compound **5**. Wavelength (λ), oscillator strength (f) and main assignment.[a]

	**λ(nm)**	**f**	**Assignment[b]**
1	412.5	0.078197348	H-1 → L 0.672160 (c= -0.81985376)<br>H → L 0.220881 (c= -0.46997996)
2	400.3	0.407768095	H-1 → L 0.213958 (c= 0.46255603)<br>H → L 0.708296 (c= -0.84160328)
3	303.2	0.050149995	H-7 → L 0.527033 (c= 0.72597043)
4	292.8	0.081651454	H-3 → L+1 0.269689 (c= 0.51931544)<br>H-2 → L+1 0.360071 (c= 0.60005938)
5	274.7	0.184215502	H-1 → L+3 0.516324 (c= 0.71855670)
6	270.7	0.228006411	H-3 → L+2 0.595363 (c= 0.77159787)
7	260.9	0.083088539	H-2 → L+3 0.548608 (c= 0.74068108)
8	255.7	0.056010415	H-5 → L+2 0.204584 (c= 0.45230916)<br>H-4 → L+2 0.249414 (c= 0.49941362)
9	247.4	0.054433146	H-8 → L+1 0.555489 (c= -0.74531161)
10	242.5	0.060551181	H-9 → L+1 0.317450 (c= 0.56342721)
11	242.2	0.059159281	H-9 → L+1 0.267184 (c= 0.51689823)
12	249.1	0.057125367	H → L+7 0.669338 (c= 0.81813085)

[a]H means HOMO and L means LUMO. [b] corresponds to the coefficient of the excitation to the CI wave function.

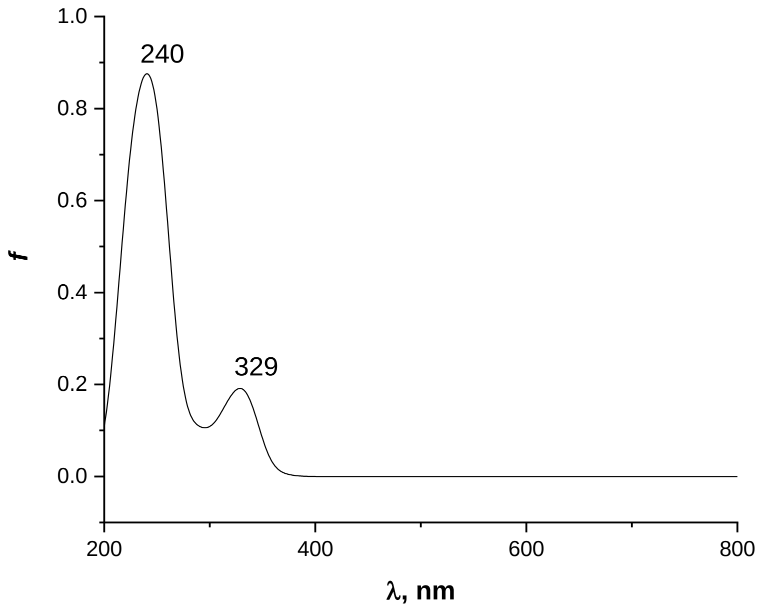

Figure S61. UV-visible spectrum of **6** calculated at the TD-PCM(toluene)/B3LYP/def2-TZVPP//BP86-D3(BJ)/def2-SVP level of theory. The middle bandwidth was arbitrary chosen as 30 nm.

Table S6. TD-PCM(toluene)/B3LYP/def2-TZVPP//BP86-D3(BJ)/def2-SVP results for compound **6**. Wavelength (λ), oscillator strength (f) and main assignment.[a]

	λ(nm)	f	Assignment[b]
1	336.6	0.125188397	H → L 0.529115 (c= 0.72740269)<br>H → L+1 0.259589 (c= 0.50949890)
2	315.4	0.087063601	H → L+2 0.552185 (c= 0.74309155)
3	252.6	0.165815299	H-2 → L+2 0.168166 (c= 0.41008035)<br>H-1 → L+3 0.139982 (c= -0.37414133)
4	253.6	0.130268443	H-5 → L 0.523999 (c= 0.72387772)
5	240.2	0.063110901	H-3 → L+1 0.198355 (c= -0.44537035)
6	238.9	0.080365279	H-3 → L+2 0.481955 (c= 0.69422982)
7	235.4	0.091991146	H → L+10 0.206154 (c= -0.45404162)<br>H → L+11 0.458145 (c= -0.67686413)
8	228.0	0.061976481	H → L+12 0.656118 (c= 0.81001085)
9	222.3	0.156407717	H-5 → L+1 0.666654 (c= -0.81648880)

[a]H means HOMO and L means LUMO. [b] corresponds to the coefficient of the excitation to the CI wave function.

Cartesian xyz coordinates and Energies in Hartrees

Compound 2

Energy (BP86-D3(BJ)/def2-SVP) = -2832.56741030 Hartree

P	0.651064000	2.287902000	-0.017831000
C	1.274502000	-0.638350000	0.018436000
N	1.831859000	-1.240032000	-1.072403000
Si	-1.758036000	0.088742000	0.013581000
B	0.220436000	0.515142000	0.010065000
Si	-2.701253000	0.600962000	2.107679000
C	2.856574000	-2.108915000	0.713520000
Si	-2.804851000	1.469005000	-1.577990000
C	2.835163000	-2.134814000	-0.671344000
N	1.869188000	-1.196406000	1.111999000
Si	-2.322377000	-2.131037000	-0.506248000
C	3.719010000	-2.901758000	-1.599730000
H	3.200637000	-3.758605000	-2.078133000
H	4.581679000	-3.309861000	-1.038897000
H	4.122498000	-2.258224000	-2.407530000
C	4.114483000	1.130273000	-0.066553000
H	4.016318000	0.491887000	0.832567000
H	5.154975000	1.521145000	-0.092210000
H	3.974637000	0.485421000	-0.955560000
C	3.347847000	3.689856000	1.454395000
H	3.218574000	3.151055000	2.415871000
H	2.692840000	4.585443000	1.486455000
H	4.403348000	4.033953000	1.394577000
C	3.287626000	3.664267000	-1.588829000
H	4.344754000	4.008365000	-1.577663000
H	2.631996000	4.559695000	-1.611284000
H	3.119684000	3.106846000	-2.533601000
Si	2.899964000	2.604913000	-0.049914000

C	3.759111000	-2.851312000	1.643794000
H	3.248751000	-3.696438000	2.151410000
H	4.175987000	-2.189018000	2.429044000
H	4.612006000	-3.273372000	1.078337000
C	1.344027000	-0.918482000	-2.436482000
H	0.344094000	-0.472770000	-2.238761000
C	1.430367000	-0.824263000	2.478512000
H	0.454181000	-0.325005000	2.290397000
C	1.152811000	-2.167981000	-3.301043000
H	0.644655000	-2.976291000	-2.739391000
H	2.107817000	-2.557244000	-3.703892000
H	0.515474000	-1.906512000	-4.168902000
C	2.195585000	0.160702000	-3.105840000
H	3.246201000	-0.166248000	-3.249747000
H	2.182936000	1.082943000	-2.493812000
H	1.770559000	0.403923000	-4.100320000
C	2.355822000	0.215878000	3.110969000
H	1.970466000	0.496303000	4.111888000
H	2.379251000	1.128624000	2.485693000
H	3.391047000	-0.163358000	3.236131000
C	1.179140000	-2.048524000	3.363240000
H	0.614477000	-1.732334000	4.262691000
H	2.116060000	-2.521081000	3.716196000
H	0.573628000	-2.808034000	2.830402000
C	-1.773904000	1.409116000	-3.178021000
H	-1.720518000	0.376209000	-3.581406000
H	-0.743258000	1.758565000	-2.957968000
H	-2.205260000	2.064268000	-3.965186000
C	-2.886130000	3.264634000	-0.968608000
H	-3.519509000	3.353627000	-0.061526000
H	-3.308025000	3.932547000	-1.750699000
H	-1.863144000	3.611530000	-0.710937000
C	-4.574530000	0.864409000	-1.949089000

H	-5.188652000	0.848459000	-1.025272000
H	-4.578145000	-0.160244000	-2.374904000
H	-5.074097000	1.536977000	-2.679684000
C	-2.395942000	-2.419265000	-2.390037000
H	-2.531893000	-3.496871000	-2.626229000
H	-1.480828000	-2.067806000	-2.903819000
H	-3.251961000	-1.865354000	-2.827488000
C	-1.051025000	-3.340281000	0.246985000
H	-1.041471000	-3.239375000	1.352650000
H	-0.022412000	-3.138716000	-0.118599000
H	-1.302869000	-4.395232000	0.003753000
C	-4.045382000	-2.580272000	0.170263000
H	-4.084910000	-2.507255000	1.276393000
H	-4.328235000	-3.616579000	-0.115377000
H	-4.812340000	-1.891011000	-0.240293000
C	-2.445107000	-0.814349000	3.361906000
H	-2.877061000	-0.545028000	4.350065000
H	-1.369715000	-1.034614000	3.513012000
H	-2.932666000	-1.751051000	3.021163000
C	-1.886881000	2.169998000	2.796646000
H	-0.788165000	2.040534000	2.882684000
H	-2.299173000	2.431399000	3.795311000
H	-2.047116000	3.026901000	2.110782000
C	-4.576489000	0.887056000	1.944751000
H	-5.030636000	1.103748000	2.935851000
H	-5.082410000	-0.005229000	1.522088000
H	-4.788850000	1.747285000	1.277105000

Compound 3

Energy (BP86-D3(BJ)/def2-SVP) = -3809.98138123 Hartree

Cl	-2.074994000	0.340002000	3.547421000
P	-1.549648000	0.587131000	1.443865000
P	0.021239000	-0.964098000	1.181064000

B	1.240929000	0.053770000	0.267887000
C	-3.105254000	-0.086053000	0.672754000
Si	2.944065000	-0.913089000	-0.219912000
Si	4.273341000	0.143485000	-1.840799000
Si	2.385383000	-3.064231000	-0.993352000
Si	4.223453000	-1.169092000	1.738780000
C	6.097119000	-0.373176000	-1.666490000
C	3.740330000	-0.331381000	-3.607819000
C	4.156844000	2.036582000	-1.639202000
C	3.090349000	-1.609892000	3.194096000
C	5.148498000	0.444781000	2.155158000
C	5.510431000	-2.552582000	1.507742000
C	0.960045000	-2.897289000	-2.243860000
C	1.821352000	-4.175588000	0.438535000
C	3.884404000	-3.875405000	-1.844758000
N	1.041471000	2.654070000	0.596192000
C	0.927034000	1.528039000	-0.161100000
C	0.471160000	3.744696000	-0.073972000
C	1.714365000	2.625325000	1.918361000
N	0.340361000	1.904748000	-1.334372000
C	0.022808000	3.269792000	-1.296219000
C	0.102783000	0.915476000	-2.415510000
C	0.388906000	5.134449000	0.466590000
C	-0.668345000	4.017932000	-2.388389000
C	0.749115000	2.836107000	3.086880000
C	2.921420000	3.569637000	1.942920000
C	-1.330822000	0.385776000	-2.402199000
C	0.546376000	1.435694000	-3.786295000
C	-3.542982000	-1.439640000	0.752949000
C	-3.852656000	0.853828000	-0.108979000
C	-4.967146000	0.400332000	-0.836957000
C	-3.491357000	2.343441000	-0.120629000
C	-5.382692000	-0.942861000	-0.809280000

C	-4.666234000	-1.836651000	0.002683000
C	-6.576028000	-1.407920000	-1.633255000
C	-2.890292000	-2.483138000	1.654366000
C	-2.344135000	-3.675572000	0.850263000
C	-3.863178000	-2.922488000	2.765565000
C	-4.073395000	3.129478000	-1.303522000
C	-3.901611000	2.997611000	1.215578000
C	-6.145709000	-2.417755000	-2.715557000
C	-7.696403000	-1.979821000	-0.743441000
H	-1.547455000	3.465432000	-2.772981000
H	0.001222000	4.234360000	-3.247010000
H	-1.033441000	4.988040000	-2.000611000
H	-0.300636000	5.736056000	-0.155688000
H	1.372006000	5.650731000	0.463156000
H	0.002963000	5.151583000	1.504700000
H	2.086069000	1.578924000	1.978417000
H	3.570154000	3.409200000	1.059370000
H	3.522787000	3.373388000	2.852692000
H	2.620046000	4.635526000	1.972467000
H	-0.072357000	2.095391000	3.075611000
H	0.304548000	3.851812000	3.088425000
H	1.305210000	2.713252000	4.038138000
H	0.772637000	0.074911000	-2.128713000
H	0.612643000	0.580816000	-4.488041000
H	1.544708000	1.913386000	-3.737480000
H	-0.174467000	2.155484000	-4.219352000
H	-1.445971000	-0.390705000	-3.184551000
H	-2.073111000	1.185204000	-2.598966000
H	-1.570355000	-0.077602000	-1.426566000
H	-5.543660000	1.109589000	-1.450265000
H	-4.992821000	-2.887745000	0.063391000
H	-2.028857000	-2.009387000	2.162043000
H	-4.766302000	-3.421788000	2.353766000

H	-4.192709000	-2.046991000	3.360309000
H	-3.365068000	-3.637586000	3.453510000
H	-3.152793000	-4.232369000	0.330117000
H	-1.820441000	-4.388181000	1.521043000
H	-1.613948000	-3.332189000	0.089236000
H	-2.382373000	2.409037000	-0.181859000
H	-5.003423000	2.953215000	1.348000000
H	-3.590466000	4.063697000	1.245544000
H	-3.437069000	2.478278000	2.077833000
H	-3.823715000	2.662822000	-2.279703000
H	-3.675231000	4.164985000	-1.305223000
H	-5.179494000	3.210615000	-1.247147000
H	-6.981100000	-0.509533000	-2.151031000
H	-8.582875000	-2.257803000	-1.351601000
H	-8.017335000	-1.243560000	0.021395000
H	-7.358332000	-2.892527000	-0.208787000
H	-7.007279000	-2.711710000	-3.351572000
H	-5.734324000	-3.342033000	-2.257443000
H	-5.359358000	-1.991935000	-3.371923000
H	2.655622000	-0.186254000	-3.772816000
H	4.286974000	0.269766000	-4.366183000
H	3.963111000	-1.401415000	-3.798361000
H	6.513402000	-0.082384000	-0.680303000
H	6.197772000	-1.474220000	-1.764103000
H	6.721761000	0.098916000	-2.455334000
H	3.108922000	2.392958000	-1.721697000
H	4.533917000	2.333430000	-0.637911000
H	4.764812000	2.566152000	-2.404124000
H	2.555219000	-2.562500000	3.003327000
H	3.675895000	-1.717410000	4.132808000
H	2.311716000	-0.834105000	3.345105000
H	6.176378000	-2.344815000	0.645415000
H	6.143741000	-2.657904000	2.415067000

H	5.012090000	-3.527320000	1.326262000
H	5.846564000	0.737435000	1.343721000
H	4.438517000	1.282601000	2.304271000
H	5.739570000	0.330669000	3.089504000
H	1.262029000	-2.282102000	-3.117331000
H	0.632462000	-3.890358000	-2.619550000
H	0.093217000	-2.408831000	-1.751314000
H	4.742314000	-3.953125000	-1.145381000
H	3.633122000	-4.900758000	-2.192553000
H	4.219415000	-3.290901000	-2.726355000
H	2.642710000	-4.336351000	1.167405000
H	0.973234000	-3.699988000	0.973911000
H	1.492878000	-5.168991000	0.063523000

Compound 4

Energy (BP86-D3(BJ)/def2-SVP) = -3864.72246187 Hartree

B	0.338078000	-0.039004000	-0.250065000
Si	2.232217000	-0.715380000	-0.036477000
Si	3.809063000	0.891926000	0.628166000
Si	2.940369000	-1.595731000	-2.102953000
Si	2.249357000	-2.478359000	1.525440000
N	-0.613181000	1.576603000	1.590281000
N	-0.057020000	2.536637000	-0.292273000
C	-0.092980000	1.338912000	0.354783000
C	-0.967807000	2.926339000	1.709292000
C	-0.617152000	3.534501000	0.514688000
C	-0.830657000	4.951505000	0.095479000
H	0.110622000	5.538542000	0.066468000
H	-1.510119000	5.451915000	0.811487000
H	-1.300798000	5.012426000	-0.906520000
C	-1.640613000	3.532124000	2.896561000
H	-0.951576000	3.670975000	3.755690000
H	-2.494792000	2.915799000	3.241353000

H	-2.038789000	4.529382000	2.628784000
C	-0.778248000	0.480080000	2.574620000
H	-0.148706000	-0.330799000	2.146692000
C	-0.209618000	0.849504000	3.948256000
H	0.801994000	1.291777000	3.857539000
H	-0.129740000	-0.067484000	4.564898000
H	-0.859504000	1.556233000	4.499130000
C	-2.219298000	-0.031052000	2.630380000
H	-2.551184000	-0.400684000	1.643487000
H	-2.929462000	0.754503000	2.957998000
H	-2.283678000	-0.874901000	3.346227000
C	0.480916000	2.639182000	-1.671652000
H	1.075636000	1.704321000	-1.773851000
C	-0.627925000	2.601257000	-2.725530000
H	-0.176710000	2.697902000	-3.733634000
H	-1.357545000	3.424807000	-2.590388000
H	-1.180071000	1.643237000	-2.692393000
C	1.430688000	3.830782000	-1.835194000
H	2.121519000	3.920936000	-0.973926000
H	0.890197000	4.788971000	-1.960051000
H	2.041327000	3.679194000	-2.747271000
C	3.044747000	2.097179000	1.893329000
H	3.783650000	2.861002000	2.218642000
H	2.710095000	1.541914000	2.794464000
H	2.162733000	2.626712000	1.476286000
C	5.320739000	0.052348000	1.425016000
H	5.779575000	-0.670519000	0.718881000
H	5.043707000	-0.503804000	2.343899000
H	6.094477000	0.801852000	1.698642000
C	4.458202000	1.893171000	-0.857595000
H	5.133690000	2.709250000	-0.521183000
H	3.638041000	2.348447000	-1.445450000
H	5.033334000	1.237950000	-1.543703000

C	2.492130000	-0.340022000	-3.460222000
H	2.988445000	0.636372000	-3.280378000
H	1.393320000	-0.181709000	-3.470008000
H	2.799623000	-0.702762000	-4.464539000
C	4.825487000	-1.870287000	-2.094226000
H	5.162822000	-2.298863000	-3.062712000
H	5.120847000	-2.573340000	-1.288626000
H	5.376275000	-0.920741000	-1.933731000
C	2.082454000	-3.247048000	-2.478071000
H	2.384807000	-3.631444000	-3.476229000
H	0.981094000	-3.109409000	-2.469028000
H	2.339584000	-4.016560000	-1.720924000
C	2.373849000	-1.837961000	3.316221000
H	2.356920000	-2.685073000	4.035627000
H	1.533136000	-1.163016000	3.569316000
H	3.315228000	-1.273368000	3.477748000
C	0.649761000	-3.486320000	1.350054000
H	0.530063000	-3.853728000	0.310101000
H	-0.240838000	-2.862220000	1.570611000
H	0.651063000	-4.359407000	2.037899000
C	3.747239000	-3.611690000	1.219933000
H	4.696813000	-3.042277000	1.291372000
H	3.701763000	-4.073562000	0.212438000
H	3.780127000	-4.430780000	1.970492000
P	-0.748113000	-1.137363000	-1.226157000
Si	-2.809510000	-0.302015000	-1.206425000
Cl	-3.505240000	-0.145569000	-3.196743000
Cl	-3.228230000	1.640365000	-0.415954000
C	-4.012988000	-1.428831000	-0.289262000
C	-3.591551000	-2.716206000	0.116907000
H	-2.565076000	-3.047039000	-0.114898000
C	-4.461170000	-3.566425000	0.820999000
H	-4.118233000	-4.566235000	1.131661000

C	-5.764039000	-3.139476000	1.129590000
H	-6.446396000	-3.803838000	1.683640000
C	-6.195558000	-1.860515000	0.731592000
H	-7.216240000	-1.523307000	0.973785000
C	-5.326211000	-1.012017000	0.028642000
H	-5.664687000	-0.006652000	-0.269419000

Compound 5

Energy (BP86-D3(BJ)/def2-SVP) = -10537.4205434 Hartree

Ga	0.045057000	1.775473000	-1.086603000
Cl	0.408036000	2.703227000	-3.077502000
Cl	-0.312545000	3.478170000	0.324578000
Ga	-0.030669000	-1.617255000	-1.196034000
Cl	-0.167396000	-2.303946000	-3.316575000
Cl	0.133191000	-3.458744000	0.068698000
Si	4.657154000	1.078284000	0.229009000
Si	3.733190000	3.092212000	1.012892000
Si	5.592424000	1.515831000	-1.892926000
Si	6.366454000	0.394219000	1.685453000
Si	-4.534483000	-1.165928000	0.164920000
Si	-6.719737000	-0.294573000	0.253005000
Si	-4.195652000	-2.368928000	2.163624000
Si	-4.462825000	-2.653193000	-1.669846000
B	3.239147000	-0.314050000	-0.028581000
B	-3.221744000	0.334179000	-0.087777000
N	3.764959000	-2.876281000	-0.421721000
N	3.037968000	-2.352990000	1.570040000
N	-3.245527000	2.364400000	1.554660000
N	-3.822606000	2.869313000	-0.490604000
P	1.637437000	0.027409000	-0.826070000
P	-1.637566000	0.103353000	-0.953491000
C	3.400302000	-1.830418000	0.366324000
C	3.579338000	-4.081951000	0.266854000

C	3.113178000	-3.749207000	1.528572000
C	3.782888000	-5.439763000	-0.318803000
H	4.854424000	-5.715929000	-0.405988000
H	3.324789000	-5.519349000	-1.324256000
H	3.296689000	-6.196641000	0.325459000
C	2.663587000	-4.658954000	2.622649000
H	3.358571000	-4.674894000	3.487423000
H	2.591495000	-5.692851000	2.235431000
H	1.657285000	-4.374152000	2.989781000
C	4.305148000	-2.661317000	-1.787992000
H	4.474649000	-1.563041000	-1.815499000
C	2.593590000	-1.470369000	2.676591000
H	2.972983000	-0.471478000	2.364271000
C	3.287190000	-3.000932000	-2.874400000
H	3.725313000	-2.781128000	-3.868746000
H	2.364870000	-2.399123000	-2.775097000
H	2.995453000	-4.070112000	-2.860607000
C	5.659047000	-3.355237000	-1.971649000
H	6.132344000	-2.983993000	-2.902102000
H	5.558311000	-4.453195000	-2.072713000
H	6.341384000	-3.135737000	-1.126725000
C	1.070955000	-1.380951000	2.759384000
H	0.793543000	-0.684158000	3.574919000
H	0.602748000	-2.363286000	2.960191000
H	0.653894000	-0.994443000	1.809571000
C	3.255533000	-1.836869000	4.008541000
H	4.342567000	-2.013435000	3.887696000
H	2.797866000	-2.731494000	4.472821000
H	3.123489000	-0.996330000	4.718418000
C	6.737708000	-1.459404000	1.441017000
H	7.568654000	-1.794136000	2.098746000
H	7.035613000	-1.651232000	0.389147000
H	5.850197000	-2.088591000	1.660333000

C	5.889498000	0.696837000	3.505436000
H	6.649082000	0.269115000	4.194936000
H	4.908237000	0.250789000	3.758083000
H	5.820269000	1.785134000	3.708866000
C	7.958573000	1.381815000	1.352119000
H	8.765082000	1.087036000	2.057725000
H	7.775804000	2.469274000	1.476372000
H	8.330081000	1.217510000	0.319885000
C	6.473252000	3.201787000	-1.855787000
H	7.249733000	3.225712000	-1.063681000
H	5.758576000	4.026213000	-1.660209000
H	6.968583000	3.402813000	-2.830187000
C	4.224602000	1.507772000	-3.201598000
H	4.638059000	1.752866000	-4.203805000
H	3.421456000	2.237623000	-2.970999000
H	3.733471000	0.514448000	-3.260801000
C	6.908272000	0.213013000	-2.346543000
H	7.320950000	0.416668000	-3.358310000
H	6.491774000	-0.812947000	-2.353118000
H	7.752438000	0.227003000	-1.626350000
C	2.457581000	2.633962000	2.345555000
H	2.966917000	2.143063000	3.202084000
H	1.705501000	1.933663000	1.930151000
H	1.915797000	3.528169000	2.718323000
C	2.989994000	4.099887000	-0.408703000
H	3.804727000	4.552477000	-1.011022000
H	2.344966000	4.915230000	-0.021702000
H	2.369408000	3.490532000	-1.093976000
C	5.102634000	4.157836000	1.801163000
H	5.573250000	3.657985000	2.672191000
H	4.684584000	5.127117000	2.149786000
H	5.900300000	4.379815000	1.062148000
C	-2.347598000	-2.427687000	2.575683000

H	-1.770668000	-2.923700000	1.768381000
H	-2.169513000	-2.975552000	3.526445000
H	-1.930480000	-1.405857000	2.682203000
C	-5.120169000	-1.582768000	3.634418000
H	-4.886687000	-2.133372000	4.571539000
H	-6.218297000	-1.619284000	3.484833000
H	-4.841041000	-0.522374000	3.787476000
C	-4.870679000	-4.133447000	1.952158000
H	-4.355549000	-4.664423000	1.126141000
H	-5.956195000	-4.113274000	1.722521000
H	-4.727675000	-4.722591000	2.883706000
C	-4.249152000	-1.633764000	-3.257047000
H	-3.257918000	-1.134405000	-3.250541000
H	-5.036496000	-0.857938000	-3.355731000
H	-4.292141000	-2.290595000	-4.152310000
C	-3.075808000	-3.942783000	-1.571117000
H	-2.163700000	-3.566839000	-2.076888000
H	-3.390619000	-4.868736000	-2.099046000
H	-2.795793000	-4.214954000	-0.534799000
C	-6.126532000	-3.584206000	-1.733332000
H	-6.128389000	-4.289482000	-2.592571000
H	-6.989148000	-2.898444000	-1.856734000
H	-6.288779000	-4.176545000	-0.809197000
C	-7.332742000	0.155256000	-1.494281000
H	-6.598183000	0.774790000	-2.044247000
H	-8.293351000	0.712645000	-1.453385000
H	-7.498299000	-0.764490000	-2.091990000
C	-6.766646000	1.264028000	1.351863000
H	-6.432967000	1.016637000	2.381190000
H	-7.797970000	1.673302000	1.417089000
H	-6.103560000	2.064959000	0.965273000
C	-7.951669000	-1.558532000	0.966486000
H	-7.934890000	-2.499109000	0.379349000

H	-8.986937000	-1.154977000	0.937814000
H	-7.715487000	-1.813518000	2.019547000
C	-3.479682000	1.833415000	0.323299000
C	-3.750455000	4.076557000	0.215494000
C	-3.377134000	3.755870000	1.510481000
C	-3.967322000	5.428537000	-0.379017000
H	-3.568521000	6.201138000	0.305415000
H	-5.039693000	5.657777000	-0.550215000
H	-3.435094000	5.536019000	-1.344544000
C	-3.058083000	4.679813000	2.638121000
H	-2.079709000	4.429915000	3.094961000
H	-3.827752000	4.672874000	3.437295000
H	-2.986157000	5.715446000	2.255369000
C	-4.247404000	2.647133000	-1.897172000
H	-4.369705000	1.542967000	-1.948537000
C	-5.607582000	3.295688000	-2.180272000
H	-6.337841000	3.074701000	-1.377014000
H	-6.010763000	2.889130000	-3.128833000
H	-5.531348000	4.393613000	-2.301661000
C	-3.162464000	3.038541000	-2.898742000
H	-2.892136000	4.111108000	-2.832644000
H	-3.523485000	2.839442000	-3.927693000
H	-2.238914000	2.449689000	-2.748377000
C	-2.857624000	1.499318000	2.693975000
H	-3.190675000	0.492209000	2.360962000
C	-3.625221000	1.845398000	3.972953000
H	-3.245646000	2.764345000	4.458905000
H	-3.502459000	1.017609000	4.698787000
H	-4.708106000	1.968036000	3.771699000
C	-1.341680000	1.444854000	2.887779000
H	-0.847294000	1.067888000	1.972494000
H	-1.110173000	0.757673000	3.726146000
H	-0.911224000	2.438505000	3.117897000

Compound 6

Energy (BP86-D3(BJ)/def2-SVP) = -5809.09468557

Ga	-2.016642000	0.399279000	-0.869585000
Cl	-2.881249000	0.971379000	-2.891754000
Cl	-2.195823000	2.306328000	0.388384000
B	1.277471000	0.089308000	-0.217581000
P	-0.044406000	-0.810351000	-1.096951000
Si	3.090399000	-0.810192000	-0.219494000
Si	4.008530000	-0.764791000	-2.384947000
Si	2.777290000	-3.061317000	0.372005000
Si	4.680096000	0.092129000	1.255780000
N	1.131140000	2.702304000	-0.081151000
N	0.765127000	1.668152000	1.806531000
N	-4.938095000	-0.249682000	-0.107631000
N	-3.668719000	-1.966761000	0.311662000
C	1.077178000	1.469594000	0.494129000
C	0.797218000	3.686928000	0.855984000
C	0.558045000	3.031398000	2.051644000
C	0.064150000	3.601454000	3.339410000
H	-0.223585000	4.659154000	3.187802000
H	0.823119000	3.576690000	4.148451000
H	-0.837754000	3.063561000	3.696451000
C	0.657668000	5.141095000	0.550764000
H	0.153105000	5.650810000	1.393507000
H	0.039521000	5.306356000	-0.354212000
H	1.634605000	5.644493000	0.395287000
C	1.467299000	2.861047000	-1.516109000
H	1.864792000	1.857634000	-1.786071000
C	0.224388000	3.109044000	-2.372744000
H	-0.326425000	4.018574000	-2.060778000
H	-0.476992000	2.254721000	-2.325801000
H	0.527998000	3.235585000	-3.431670000

C	2.582626000	3.889403000	-1.731764000
H	2.227245000	4.931840000	-1.615754000
H	2.971660000	3.790238000	-2.764632000
H	3.423763000	3.720164000	-1.030469000
C	0.615521000	0.515989000	2.722742000
H	1.202889000	-0.280765000	2.214921000
C	-0.837609000	0.040616000	2.798195000
H	-1.514678000	0.833386000	3.172789000
H	-0.903782000	-0.837207000	3.472527000
H	-1.188024000	-0.256901000	1.791223000
C	1.239812000	0.765894000	4.099018000
H	2.230790000	1.253143000	4.012298000
H	1.382877000	-0.207407000	4.609413000
H	0.594021000	1.385770000	4.750498000
C	1.658882000	-3.115895000	1.915749000
H	2.145957000	-2.626395000	2.784616000
H	1.408761000	-4.159742000	2.204648000
H	0.716417000	-2.573576000	1.692548000
C	4.430434000	-3.919818000	0.775164000
H	5.115272000	-3.882390000	-0.096945000
H	4.270987000	-4.987661000	1.039796000
H	4.945495000	-3.431443000	1.628261000
C	1.910739000	-4.016015000	-1.023098000
H	0.986218000	-3.472860000	-1.314606000
H	1.646725000	-5.047865000	-0.703783000
H	2.558903000	-4.088642000	-1.921107000
C	2.647026000	-1.060561000	-3.671111000
H	1.830463000	-0.316732000	-3.565876000
H	2.183614000	-2.058409000	-3.529798000
H	3.055935000	-1.003609000	-4.703305000
C	4.819421000	0.925494000	-2.733478000
H	4.080279000	1.746640000	-2.645523000
H	5.241165000	0.957456000	-3.761378000

H	5.642152000	1.138150000	-2.019801000
C	5.341972000	-2.111720000	-2.576728000
H	5.810824000	-2.062253000	-3.583426000
H	4.899752000	-3.122743000	-2.457907000
H	6.143119000	-1.998991000	-1.817982000
C	4.498736000	-0.623879000	3.014571000
H	4.750770000	-1.704530000	3.018365000
H	3.466310000	-0.523508000	3.401101000
H	5.182949000	-0.112929000	3.726214000
C	4.497849000	1.988329000	1.347390000
H	5.251019000	2.434437000	2.032350000
H	3.489002000	2.285705000	1.701993000
H	4.644723000	2.434593000	0.341492000
C	6.453466000	-0.317844000	0.693192000
H	6.675783000	0.109798000	-0.305864000
H	6.594606000	-1.416935000	0.628222000
H	7.201793000	0.081824000	1.411463000
C	-3.642405000	-0.674622000	-0.126184000
C	-5.787315000	-1.272058000	0.329180000
C	-4.980632000	-2.368156000	0.589012000
C	-5.385775000	-3.724576000	1.070730000
H	-5.105312000	-3.899456000	2.130344000
H	-4.928046000	-4.533519000	0.467211000
H	-6.483932000	-3.836364000	0.993482000
C	-7.274699000	-1.172827000	0.447797000
H	-7.592187000	-0.268236000	1.002546000
H	-7.667390000	-2.051420000	0.993816000
H	-7.771691000	-1.144855000	-0.544305000
C	-5.326961000	1.137183000	-0.476710000
H	-4.384012000	1.590245000	-0.834979000
C	-6.301755000	1.165712000	-1.655623000
H	-6.400512000	2.212229000	-2.007175000
H	-7.316561000	0.801728000	-1.399373000

H	-5.892171000	0.572315000	-2.496421000
C	-5.764861000	1.934937000	0.754751000
H	-4.963275000	1.914984000	1.518849000
H	-6.710599000	1.566529000	1.202575000
H	-5.920417000	2.992684000	0.461883000
C	-2.446086000	-2.805315000	0.386583000
H	-1.613043000	-2.078154000	0.259231000
C	-2.265230000	-3.471670000	1.752707000
H	-1.217709000	-3.821475000	1.837739000
H	-2.921807000	-4.351117000	1.902088000
H	-2.445460000	-2.749578000	2.574227000
C	-2.357614000	-3.766149000	-0.801159000
H	-2.415330000	-3.202852000	-1.753659000
H	-3.156352000	-4.536876000	-0.788092000
H	-1.376958000	-4.281767000	-0.779624000

References

[1] G. R. Fulmer, A. J. M. Miller, N. H. Sherden, H. E. Gottlieb, A. Nudelman, B. M. Stoltz, J. E. Bercaw, K. I. Goldberg, *Organometallics* **2010**, *29*, 2176-2179.

[2] (a) G. M. Sheldrick, *Acta Crystallogr. A* **2008**, *64*, 112-122; (b) G. M. Sheldrick, *Acta Cryst. A* **2015**, *71*, 3-8; (c) G. M. Sheldrick, *Acta Cryst. C* **2015**, *71*, 3-8.

[3] N. Kuhn, T. Kratz, *Synthesis* **1993**, *1993*, 561-562.

[4] H. Braunschweig, M. Colling, C. Kollann, U. Englert, *J. Chem. Soc., Dalton Trans.* **2002**, 2289-2296.

[5] U. Englich, K. Hassler, K. Ruhlandt-Senge, F. Uhlig, *Inorg. Chem.* **1998**, *37*, 3532-3537.

[6] C. Kirst, J. Tietze, M. Ebeling, L. Horndasch, K. Karaghiosoff, *J Org. Chem.* **2021**, *86*, 17337-17343.

[7] M. J. Frisch, G. W. Trucks, H. B. Schlegel, G. E. Scuseria, M. A. Robb, J. R. Cheeseman, G. Scalmani, V. Barone, B. Mennucci, G. A. Petersson, H. Nakatsuji, M. Caricato, X. Li, H. P. Hratchian, A. F. Izmaylov, J. Bloino, G. Zheng, J. L. Sonnenberg, M. Hada, M. Ehara, K. Toyota, R. Fukuda, J. Hasegawa, M. Ishida, T. Nakajima, Y. Honda, O. Kitao, H. Nakai, T. Vreven, J. J. A. Montgomery, J. E. Peralta, F. Ogliaro, M. Bearpark, J. J. Heyd, E. Brothers, K. N. Kudin, V. N. Staroverov, R. Kobayashi, J. Normand, K. Raghavachari, A. Rendell, J. C. Burant, S. S. Iyengar, J. Tomasi, M. Cossi, N. Rega, J. M. Millam, M. Klene, J. E. Knox, J. B. Cross, V. Bakken, C. Adamo, J. Jaramillo, R. Gomperts, R. E. Stratmann, O. Yazyev, A. J. Austin, R. Cammi, C. Pomelli, J. W. Ochterski, R. L. Martin, K. Morokuma, V. G. Zakrzewski, G. A. Voth, P. Salvador, J. J. Dannenberg, S. Dapprich, A. D. Daniels, O. Farkas, J. B. Foresman, J. V. Ortiz, J. Cioslowski, D. J. Fox, Gaussian 16, Revision B.01. Gaussian, Inc., Wallingford CT, **2016**.

[8] (a) A. D. Becke, *Phys. Rev. A* **1988**, *38*, 3098-3100; (b) J. P. Perdew, *Phys. Rev. B* **1986**, *33*, 8822-8824.

[9] S. Grimme, J. Antony, S. Ehrlich, H. Krieg, *J. Chem. Phys.* **2010**, *132*.

[10] S. Grimme, S. Ehrlich, L. Goerigk, *J. Comp. Chem.* **2011**, *32*, 1456-1465.

[11] F. Weigend, R. Ahlrichs, *Phys. Chem. Chem. Phys.* **2005**, *7*, 3297-3305.

[12] C. Y. Peng, P. Y. Ayala, H. B. Schlegel, M. J. Frisch, *J. Comp. Chem.* **1996**, *17*, 49-56.

[13] J. W. McIver, Komornic.A, *J. Am. Chem. Soc.* **1972**, *94*, 2625-2633.

[14] (a) A. E. Reed, R. B. Weinstock, F. Weinhold, *J. Chem. Phys.* **1985**, *83*, 735-746; (b) A. E. Reed, L. A. Curtiss, F. Weinhold, *Chem. Rev.* **1988**, *88*, 899-926.

[15] E. D. Glendening, C. R. Landis, F. Weinhold, *J. Comp. Chem.* **2019**, *40*, 2234-2241.

[16] R. F. W. Bader, *Atoms in Molecules: A Quantum Theory*, , Clarendon, Oxford, **1990**.

[17] T. A. Keith, T. K. Gristmill, 19.02.13 ed., Overland Park KS, USA (aim.tkgristmill.com), **2019**.

[18] F. Neese, 4.2.1 ed., Max-Planck-Institut für Kohlenforschung; Mulheim an der Ruhr, Germany, **2019**.

[19] C. Lee, W. Yang, R. G. Parr, *Phys. Rev. B* **1988**, *37*, 785-789.

[20] K. Morokuma, *J. Chem. Phys.* **1971**, *55*, 1236-1244.

[21] (a) T. Ziegler, A. Rauk, *Inorg. Chem.* **1979**, *18*, 1558-1565; (b) T. Ziegler, A. Rauk, *Inorg. Chem.* **1979**, *18*, 1755-1759.

[22] F. M. Bickelhaupt, N. M. M. Nibbering, E. M. Van Wezenbeek, E. J. Baerends, *J. Phys. Chem.* **1992**, *96*, 4864-4873.

[23] (a) F. M. Bickelhaupt, E. J. Baerends, in *Reviews in Computational Chemistry, Vol. 15* (Eds.: K. B. Lipkowitz, D. B. Boyd), **2000**, pp. 1-86; (b) G. te Velde, F. M. Bickelhaupt, E. J. Baerends, C. Fonseca Guerra, S. J. A. van Gisbergen, J. G. Snijders, T. Ziegler, *J. Comp. Chem.* **2001**, *22*, 931-967.

[24] J. Krijn, E. J. Baerends, *Fit Functions in the HFS-Method* **1984**.

[25] E. Van Lenthe, E. J. Baerends, J. G. Snijders, *J. Chem. Phys.* **1993**, *99*, 4597-4610.
